# Socioeconomic Inequalities in Neglected Tropical Diseases: A Systematic Review

**DOI:** 10.1371/journal.pntd.0004546

**Published:** 2016-05-12

**Authors:** Tanja A. J. Houweling, Henrike E. Karim-Kos, Margarete C. Kulik, Wilma A. Stolk, Juanita A. Haagsma, Edeltraud J. Lenk, Jan Hendrik Richardus, Sake J. de Vlas

**Affiliations:** 1 Department of Public Health, Erasmus MC University Medical Center Rotterdam, Rotterdam, The Netherlands; 2 Center for Tobacco Control Research and Education, University of California at San Francisco, San Francisco, California, United States of America; 3 Institute of Health Policy & Management, Erasmus University Rotterdam, Rotterdam, The Netherlands; Swiss Tropical and Public Health Institute, SWITZERLAND

## Abstract

**Background:**

Neglected tropical diseases (NTDs) are generally assumed to be concentrated in poor populations, but evidence on this remains scattered. We describe within-country socioeconomic inequalities in nine NTDs listed in the London Declaration for intensified control and/or elimination: lymphatic filariasis (LF), onchocerciasis, schistosomiasis, soil-transmitted helminthiasis (STH), trachoma, Chagas’ disease, human African trypanosomiasis (HAT), leprosy, and visceral leishmaniasis (VL).

**Methodology:**

We conducted a systematic literature review, including publications between 2004–2013 found in Embase, Medline (OvidSP), Cochrane Central, Web of Science, Popline, Lilacs, and Scielo. We included publications in international peer-reviewed journals on studies concerning the top 20 countries in terms of the burden of the NTD under study.

**Principal findings:**

We identified 5,516 publications, of which 93 met the inclusion criteria. Of these, 59 papers reported substantial and statistically significant socioeconomic inequalities in NTD distribution, with higher odds of infection or disease among poor and less-educated people compared with better-off groups. The findings were mixed in 23 studies, and 11 studies showed no substantial or statistically significant inequality. Most information was available for STH, VL, schistosomiasis, and, to a lesser extent, for trachoma. For the other NTDs, evidence on their socioeconomic distribution was scarce.

The magnitude of inequality varied, but often, the odds of infection or disease were twice as high among socioeconomically disadvantaged groups compared with better-off strata. Inequalities often took the form of a gradient, with higher odds of infection or disease each step down the socioeconomic hierarchy. Notwithstanding these inequalities, the prevalence of some NTDs was sometimes also high among better-off groups in some highly endemic areas.

**Conclusions:**

While recent evidence on socioeconomic inequalities is scarce for most individual NTDs, for some, there is considerable evidence of substantially higher odds of infection or disease among socioeconomically disadvantaged groups. NTD control activities as proposed in the London Declaration, when set up in a way that they reach the most in need, will benefit the poorest populations in poor countries.

## Introduction

The burden of Neglected Tropical Diseases (NTDs) is heavily concentrated in low- and middle-income countries [1]. Not only between countries but also within countries, NTDs are often assumed to be concentrated in the poorest populations [2]. Poverty is usually seen as a root cause of NTDs because of its association with living and working conditions and access to preventive and curative health services [3]. In turn, NTDs have strong impoverishing effects because of the absence of social protection systems (including health insurance to protect people against catastrophic health expenditures and sickness and disability insurance to protect people against loss of income in the case of sickness or disability) in most developing countries [4].

The almost omnipresent assumption about the unequal distribution of NTD prevalence across socioeconomic strata contrasts with the scattered nature of the literature on this subject. Empirical evidence on the socioeconomic distribution of NTDs comes from studies conducted from a variety of disciplinary and methodological perspectives (cf. [5–8]). Sometimes, socioeconomic inequalities in NTD prevalence are the main study focus, but more often, socioeconomic position (SEP) is examined as one of a broad range of determinants or merely as a potential confounder of other relationships of interest. So far, there has been no effort to bring together this dispersed literature and describe, across a broad range of NTDs, the extent of socioeconomic inequalities in infection or disease prevalence.

By contrast, the literature on socioeconomic inequalities in health more generally is extensive. From this literature, we know that most health outcomes are unequally distributed, with people at the lower end of the socioeconomic ladder having lower chances of leading a long and healthy life compared with better-off groups within the same country [1,9,10]. This is the case in high-income countries, where the literature on socioeconomic inequalities in health is extensive [10], but especially also in low- and middle-income countries, where the body of literature is growing, in particular with regard to inequalities in child health outcomes [9]. From this body of work, we know that socioeconomic inequalities in health are often substantial. It is generally assumed that socioeconomic inequalities in NTDs are also large and that control strategies would benefit poor populations most.

We used our combined expertise in health inequalities research and NTD research to bring together the recent evidence on the distribution of NTD prevalence—and/or prevalence of the underlying infection—across socioeconomic strata within countries and to summarize the magnitude and pattern of these inequalities. With this aim, we conducted a systematic literature review for nine NTDs listed in the London Declaration for intensified control and/or elimination, including those that are controlled through preventive chemotherapy (PCT) (i.e., lymphatic filariasis [LF], onchocerciasis, schistosomiasis, soil-transmitted helminths [STH], and trachoma) and those controlled through intensified disease management (IDM) (i.e., Chagas’ disease, human African trypanosomiasis [HAT], leprosy, and visceral leishmaniasis [VL]).

## Methods

We conducted a systematic literature review on the socioeconomic distribution within endemic countries of LF, onchocerciasis, schistosomiasis, STH, trachoma, Chagas’ disease, HAT, leprosy, and VL. Our search protocol is provided in S1 Supporting Information.

The search included all publications between 2004–2013 in the following databases: Embase, Medline (OvidSP), Cochrane Central, Web of Science, Popline, Lilacs, and Scielo. Also, the most relevant results from Google Scholar were screened, and we searched for articles that were present in PubMed but not yet available in Medline. The search terms used included the NTD names, types of epidemiological data, and indicators of SEP (S1 Supporting Information). The search was completed on 18 December 2013.

Papers were included based on the following predefined criteria: published in an international peer-reviewed journal (i.e., journals with an impact factor) of any language between 2004 and 2013; study period between 2000 and 2013; and reporting estimates of the association between SEP and NTD prevalence, or prevalence of the underlying infection, with a measure of statistical significance (e.g., *p*-value or 95% confidence interval). We only included studies about the top 20 countries in terms of burden for the NTD under study (Global Burden of Disease [GBD] 2010) in order to focus our review on the most significant endemic countries. The included countries comprised almost 90% of the global burden for the studied NTDs. We excluded papers that did not report the study period (seven papers).

First-stage screening on the basis of title and abstract was done by HKK, who, when in doubt, discussed findings with MCK and TAJH, only excluding papers that were definitely not: about the NTD under study, about the association between SEP and the NTD, or within the above-mentioned study and publication period. In the second stage, the full text of the papers was reviewed by HKK, MCK, and TAJH, with at least two of the authors involved in the decision about inclusion or exclusion. Data were extracted by HKK in a sheet including author, publication date, study aim, NTD measurement, study design, statistical methods (including whether clustering was taken into account in the statistical analyses), sampling design, sample size, indicator of SEP, NTD prevalence, and univariate and multivariate association between SEP and the NTD. The extraction sheets were checked for correctness by JAH, TAJH, and MCK. Support with translation of Spanish and Portuguese papers was provided by EL and EdV.

### Analytical framework

**Fig 1:**
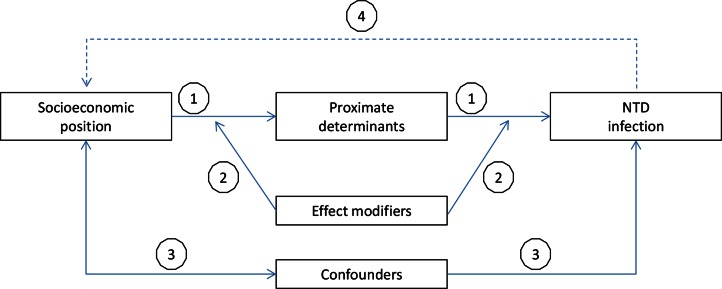
Analytical framework of the relationship between SEP and NTD infection. Note that we use NTD infection to signify the NTD and/or the underlying infection. Pathway (1) shows the effect of SEP on NTD infection via more proximate determinants of such infection, e.g., (lack of) sanitation facilities. The effect of SEP on NTD infection may be modified (2), e.g., by age. Associations between SEP and NTD infection may be partly explained by confounders (3), e.g., sex. Finally, NTD infection can also affect SEP (4). Dashed line: not the focus of our review (part of other reviews in this series). The framework is based on [11–16].

The analytical framework (Fig 1) that we used for this review is based on the extensive literature on socioeconomic inequalities in health [9–14]. The core of the framework, and the focus of our review, is the association between SEP and NTD infection and, in particular, the distribution of disease or infection prevalence across socioeconomic strata. Here, and in the remainder of the manuscript, we use NTD infection to signify the prevalence of the NTD and/or the underlying infection.

Usually, health outcomes are unequally distributed, with worse outcomes among socioeconomically disadvantaged groups compared with better-off strata [10]. Taking infection prevalence as an example, the magnitude of inequality in infection prevalence between socioeconomic groups can be measured using the ratio of the odds (OR) of infection between lower and higher socioeconomic strata or other summary measures of inequality [15]. Sometimes, only disease or infection prevalence rates are described across socioeconomic strata, combined with tests for differences in rates between strata. We included papers reporting any measure of association between SEP and NTD infection as well as papers only presenting SEP-specific diseases or infection prevalence rates.

SEP, in the context of research on low- and middle-income countries, is typically measured using indicators of educational attainment and/or economic status [14]. In such countries, household ownership of assets is often used as measure of economic status [16]. Sometimes, other dimensions of position in the socioeconomic hierarchy are studied, such as caste or occupational class. Ecological and multilevel designs usually (also) use aggregate measures of SEP, such as village-level per capita income, the percentage of adults unemployed, or the percentage of households owning their own home in a given geographical area. We have been inclusive when selecting papers, including studies reporting any measure that, according to the authors of those papers, indicated (individual or aggregate level) position in the socioeconomic hierarchy in their specific context.

SEP influences NTD infection via more proximate determinants of such infection (Fig 1, pathway 1). These proximate determinants vary by NTD and include, for example, hygiene behaviours, access to clean water and sanitation facilities, environmental hygiene, exposure to infection through working conditions, and access to health services. The relationship between these proximate determinants and NTD infection is the subject of a broad literature and is, by itself, not the focus of our review. An implication of the causal pathway from SEP via proximate determinants to NTD infection is that statistical adjustment for such proximal determinants generally reduces the magnitude of socioeconomic inequality in NTD infection. In other words, the association between SEP and NTD infection is partially explained by intermediate variables. As our paper focusses on the description of the magnitude of socioeconomic inequality in NTD infection, we focus in our description on associations unadjusted for these proximal determinants.

Potential confounders (Fig 1, pathway 3) of the relationship between SEP and NTD infection can include, for example, age and sex. A Brazilian study found, for instance, that elderly people tend to be richer and, independently of SEP, have higher odds of having trachoma [17]. Age and sex (but also other factors) can also be effect modifiers of the relationship between SEP and NTD infection (Fig 1, pathway 2). In other words, the relationship between SEP and NTD infection can be stronger or weaker depending on age or sex. Whenever available, we have presented data about the relationship between SEP and NTD infection stratified by age and sex. The magnitude of socioeconomic inequality in NTD prevalence can also vary by the specific outcome studied (e.g., for trachoma, whether trachomatous inflammation—follicular [TF], trachomatous inflammation—intense [TI], trachomatous scarring [TS], or trachomatous trichiasis [TT] was studied) and the detection method used (e.g., based on blood samples or stool samples). We have presented findings stratified by specific outcome and detection method whenever available.

Finally, SEP not only influences the odds of NTD infection; NTD infection can also affect SEP through income lost because of illness, costs of medical care, and, in the case of children, impeded school attendance and performance (Fig 1, pathway 4). The socioeconomic consequences of NTDs are the subject of separate papers in this series [4].

This review is compliant with the Preferred Reporting Items for Systematic Reviews and Meta-Analyses (PRISMA) checklist (S2 Supporting Information) [18].

## Results

### Overview

**Fig 2:**
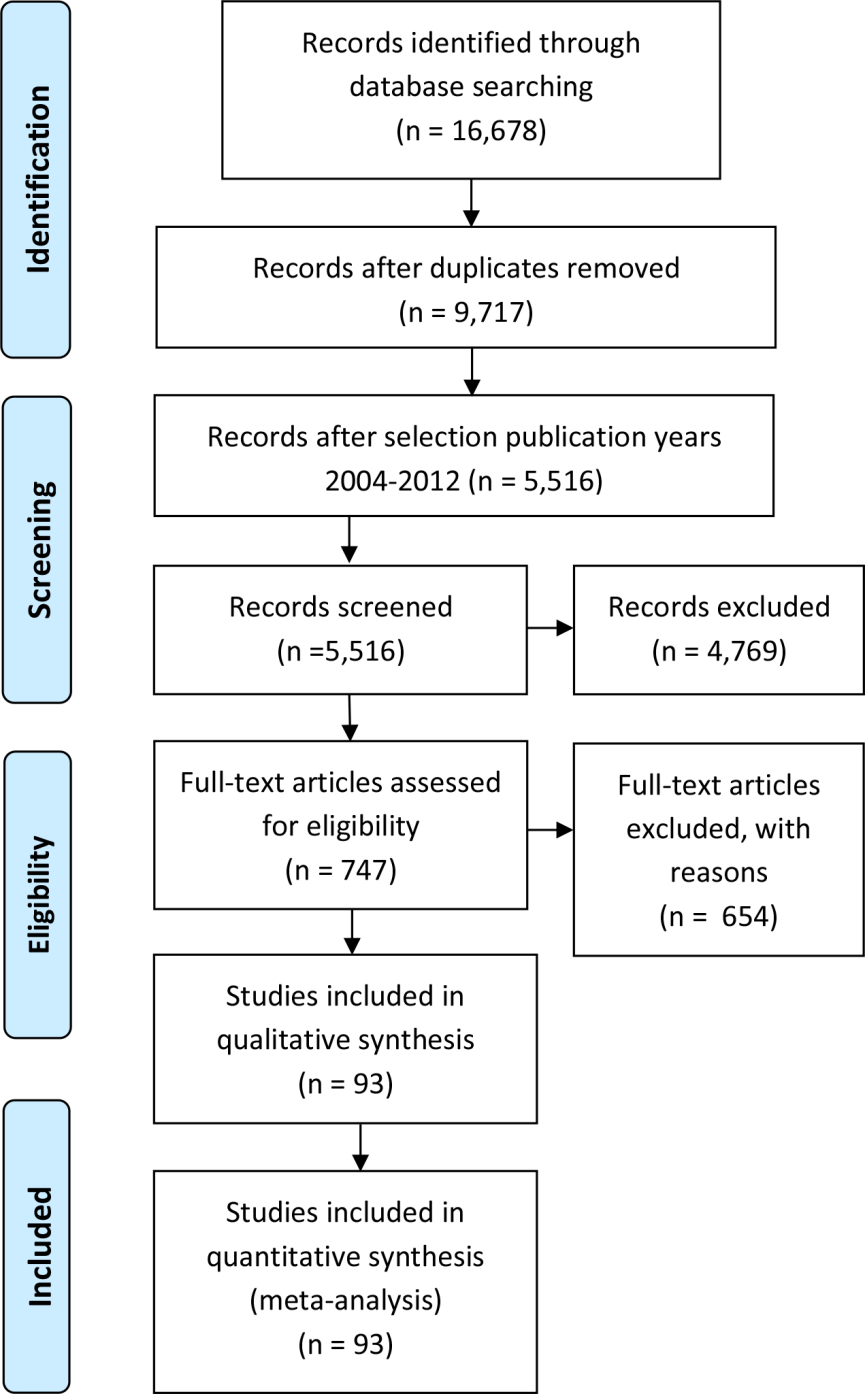
PRISMA flow diagram.

**Fig 3:**
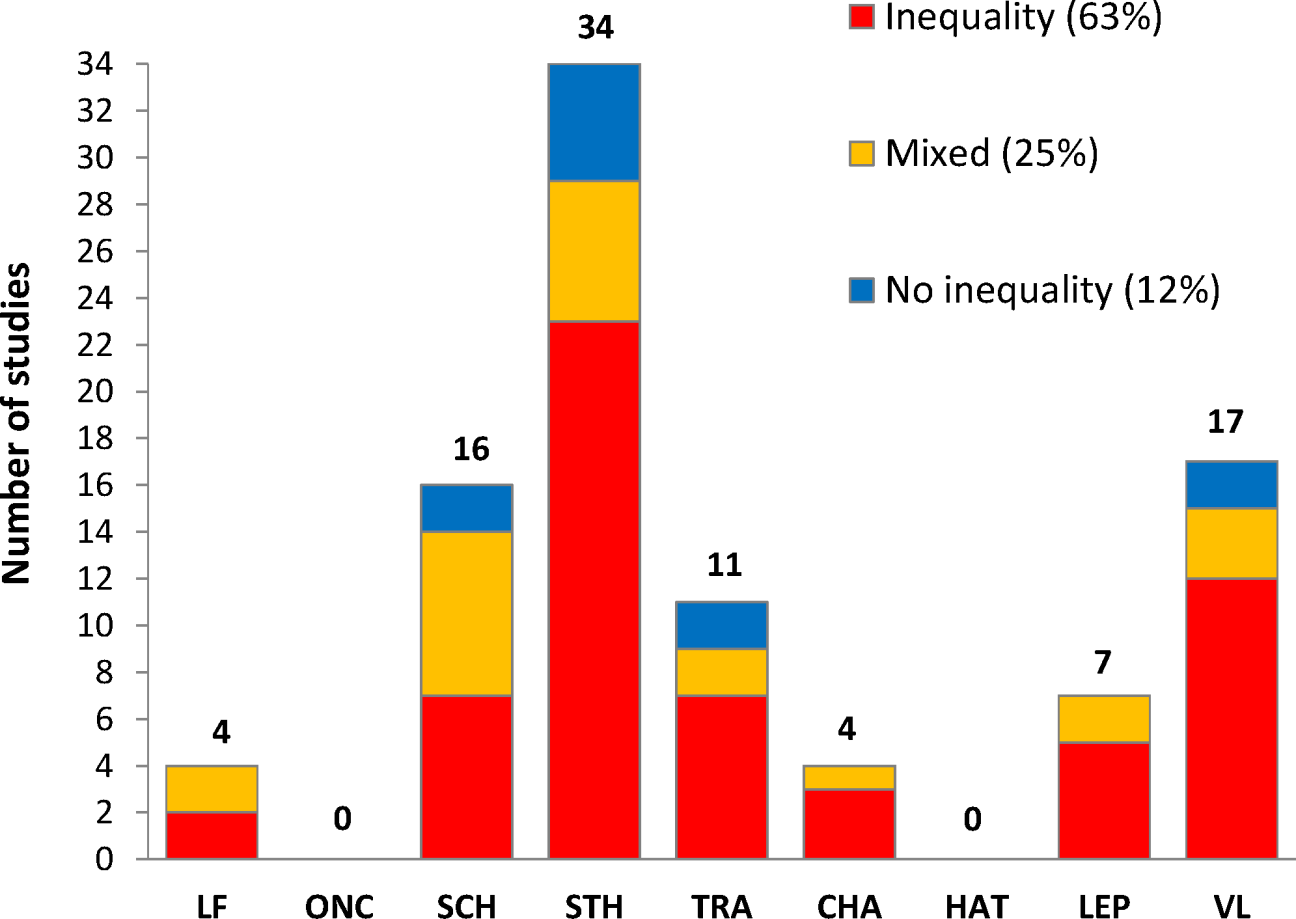
Number of papers reporting greater odds of infection among lower socioeconomic strata than among higher socioeconomic strata, number of papers reporting mixed results, and number of papers reporting no inequality or a reverse association, 2004–2013. *Inequality*: Statistically significant (*p* < 0.05) inequality in NTD distribution, with greater odds of infection among lower socioeconomic strata. This also includes papers reporting statistically significant inequality for one SEP indicator and nonsignificant inequality of at least 50% greater odds of infection among lower strata for another SEP indicator. The same criterion was used for socioeconomic inequality in NTD prevalence in one age group and not in another age group and for one NTD outcome measure and not for another NTD outcome measure. *Mixed*: Studies reporting a combination of statistically significant inequality—with greater odds of infection among lower socioeconomic strata—for one SEP indicator, age group, or NTD outcome measure and no such inequality (or reversed pattern) for another SEP indicator, age group, or NTD outcome measure. *No inequality*: Studies reporting no substantial and statistically significantly greater odds of infection among lower socioeconomic strata or reporting a reverse pattern, with greater odds of infection among higher strata. Of the 93 publications included in the review, two studies (Balen et al. 2011 and Steinmann et al. Acta Tropica 2007) reported findings for both schistosomiasis and STH separately. In the figure, we included these studies under both schistosomisasis and STH. Two other studies that reported on combined schistosomiasis and STH infection in the same individuals were not included in this figure (one reported inequality, the other no inequality).

5,516 unique papers published between 2004 and 2013 in international peer-reviewed journals were identified (Fig 2). Of these, 4,769 were excluded on the basis of title and abstract. The full text of the remaining 747 papers was reviewed, of which 93 papers met the inclusion criteria. Of the 93 studies that presented data on the socioeconomic distribution of NTDs, almost two-thirds (59/93) reported substantial and statistically significant inequalities (Fig 3). The findings were mixed in a quarter of the studies (23/93), and over 10% (11/93) showed no substantial or statistically significant inequality in NTD distribution. Most information was available for STH (34 papers), schistosomiasis (16 papers), VL (17 papers), and, to a lesser extent, for trachoma (11 papers). For the other NTDs, there is a paucity of evidence, with no recent papers identified for onchocerciasis and HAT.

### Lymphatic filariasis

**Fig 4:**
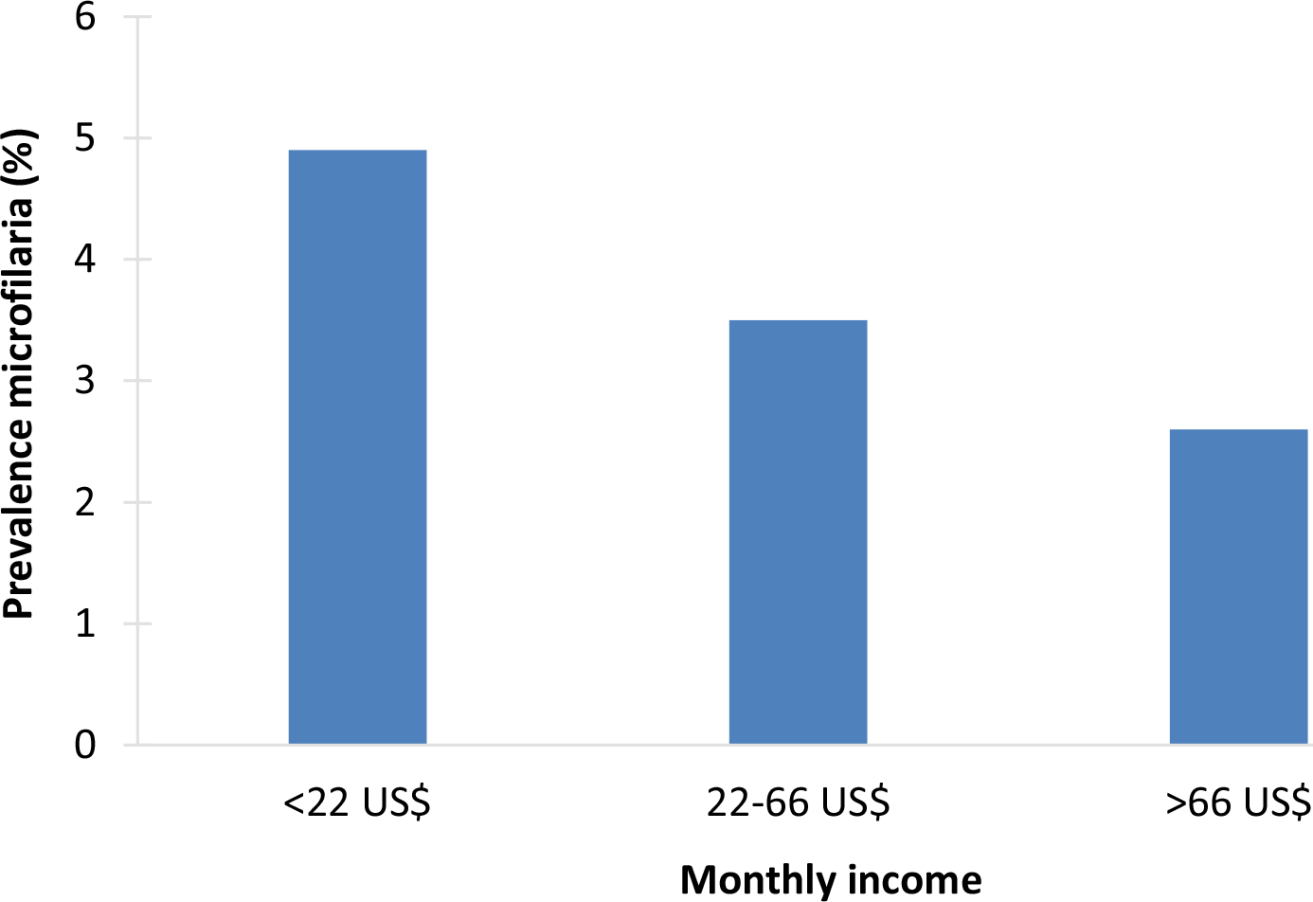
Example of socioeconomic inequalities in LF: association between microfilaria prevalence (%) and household monthly income (US$) in Karimnagar district, India (2004–2007) [20]. The study was performed in Karimnagar district (Andhra Pradesh, India), which is a filariasis-endemic region where a mass drug administration (MDA) program has been active since 2004. Blood samples were taken from 5,394 inhabitants of all ages from 30 villages and tested for *Wuchereria bancrofti* microfilaria. Socioeconomic information was collected through an interview with the household head or other family members. Household monthly income was divided into three categories (<US$22; US$22–US$66; >US$66; currency rate used: 1 Indian Rupee = US$0.022, January 2006). In total, 3.7% of the blood samples tested positive for microfilaria. Microfilaria prevalence was statistically significantly (*p* = 0.02) associated with household income, with the poorer households being more affected than the richer households.

Four papers on lymphatic filariasis met the inclusion criteria (India [GBD #1] two [19,20]; Tanzania [GBD #11] two [21,22]) (S1 Table). All were cross-sectional studies, representing the general population in a defined geographical area: Chennai city in Tamil Nadu (mass drug administration [MDA] status not reported) and endemic villages in Andhra Pradesh (Fig 4) (MDA since 2004, 58% of respondents participated) in India and the cities of Tanga (59% of respondents participated in MDAs) and Dar es Salaam (MDA in 2006 and 2007, 19% of respondents participated) in Tanzania [21,22]. The Tanzanian papers also included schoolchildren in addition to the general population.

In the Indian studies, the prevalence of *Wuchereria bancrofti* microfilaria was nearly twice as high among poor villagers (4.9%) and in poor urban neighbourhoods (1.3%) compared with richer ones (rural: 2.6%, *p* = 0.02; urban: 0.5%, *p* = 0.01). The prevalence was also higher among less-educated people (in the rural study) than among the better educated, but this association became only statistically significant after adjusting for age and several intermediate determinants.

**Fig 5:**
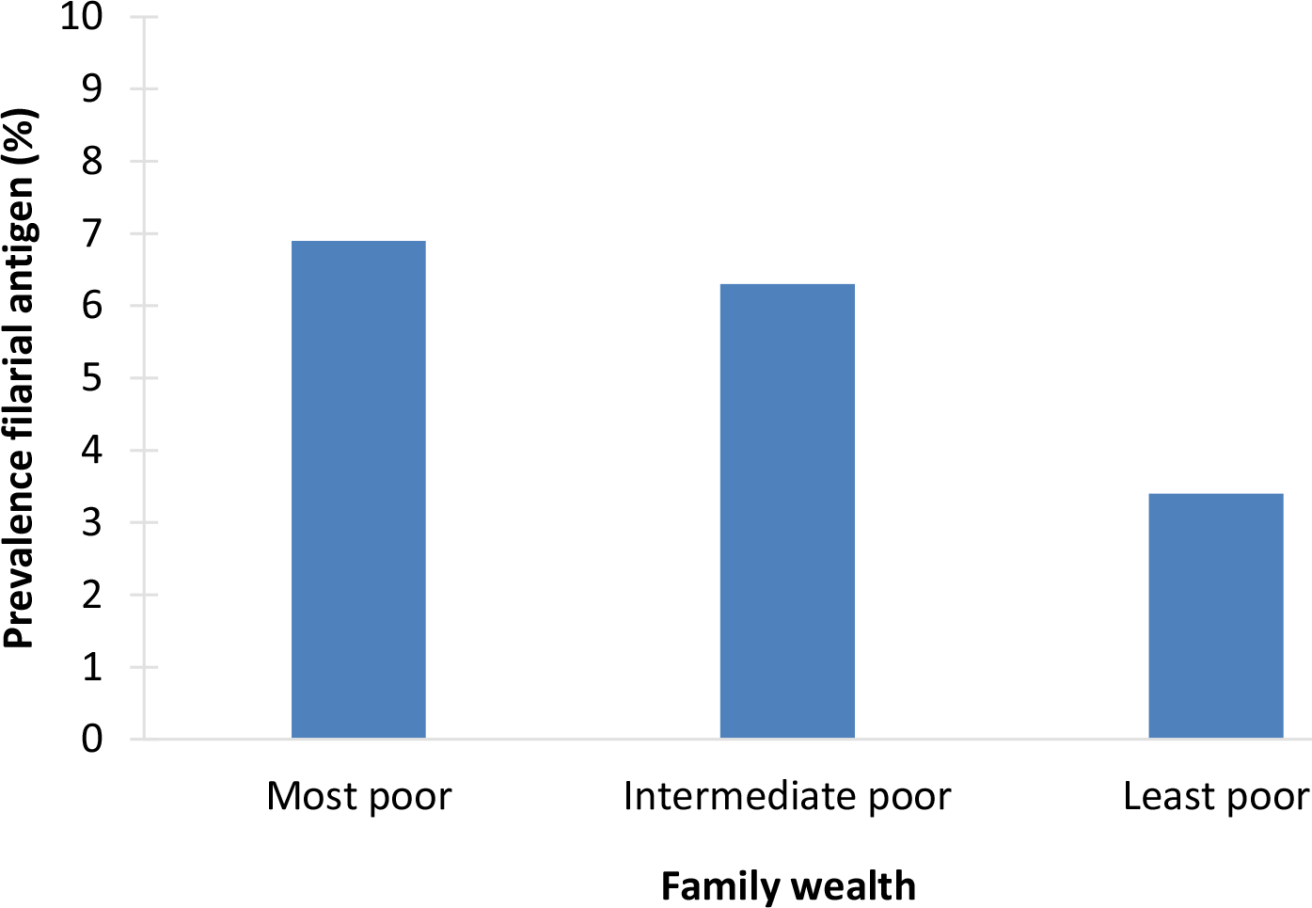
Example of socioeconomic inequalities in LF: association between filarial antigen prevalence (%) and family wealth in the city of Tanga, eastern Tanzania (2012) [22]. The study was conducted in the city of Tanga in eastern Tanzania, along the Indian Ocean, where a mass drug administration program (MDA) has been active since 2004. Two urban and one peri-urban ward were selected as being representative for the city, and 960 children aged five to 16 years from public primary schools were examined for circulating filarial antigens (CFA) in their blood. Of these children, 895 also filled in a questionnaire. Ownership of a fridge and TV was used as indicator of family wealth; this information was aggregated to constitute a measure of wealth at the ward level. To create the figure, we divided the wards into three groups according to the family wealth measures: “most poor,” “intermediate poor,” and “least poor.” The overall CFA prevalence was 5.5%. CFA prevalence was statistically significantly lower in the least poor as compared to the intermediate and most poor (*p* = 0.04).

The Tanzanian studies reported that the prevalence of circulating filarial antigens (CFA) among school children was higher in children from poorer neighbourhoods (Fig 5). Among community members (aged ≥ ten years), such neighbourhood-level association was only found in one study [21,22]. At the household level, no association was found between economic status and CFA prevalence (only examined for school children). The authors suggested that this was perhaps due to the importance of community-level factors like poor water and sanitation conditions.

In sum, there is a paucity of evidence on socioeconomic inequalities in LF prevalence. The currently available studies, from India and Tanzania only, present a mixed picture, with an association between SEP and LF prevalence in some age groups when using some SEP measures but not in other age groups and/or when using other SEP measures.

### Onchocerciasis

No relevant papers were found.

### Schistosomiasis

16 relevant papers were found (Nigeria [GBD #1] one, on *Schistosoma haematobium* [23]; China [GBD#2] six, on *S*. *japonicum* [24–28]; Sudan [GBD#5] two [one on *S*. *haematobium*, one on *S*. *mansoni*] [6,29]; Côte d’Ivoire [GBD #14] five, on *S*. *mansoni* [30–34]; Uganda [GBD #15] two, on *S*. *mansoni* [35,36]) (S2 Table). Moreover, we found two relevant studies about combined schistosomiasis and STH infection (reported on under STH). Four papers from Côte d’Ivoire report about the same study population [31–34]; the same is true for two studies from China [25,26]. All studies used a cross-sectional design.

### S. japonicum

**Fig 6:**
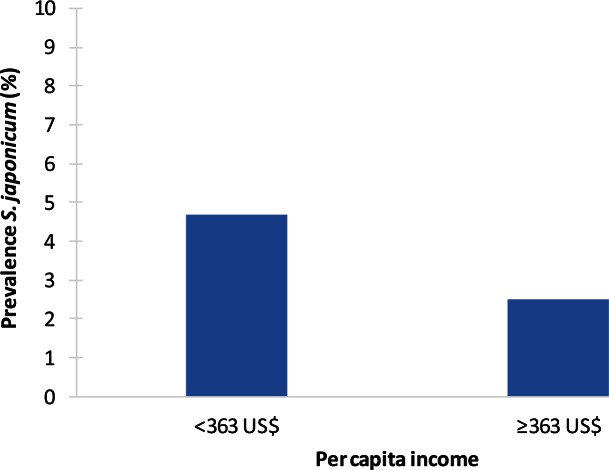
Example of socioeconomic inequalities in schistosomiasis: association between *S*. *japonicum* and income per capita at village level in Hunan province, China (2005) [28]. The studied villages represent four types of areas: lake-embankment, lake-beach, inside embankment, and hill areas. A total of 10,245 residents aged six years and older from 16 villages were included in the study. The presence of antibodies to *S*. *japonicum* was screened using the indirect hemagglutination (IHA) test, and stool samples were examined for IHA-positive cases. Per capita income was measured at the village level (currency rate used: 1 Yuan = US$0.121, July 2005). The overall infection prevalence was 4.1%. Infection prevalence was higher in poorer villages, (*p* < 0.001).

The Chinese studies were conducted in the general population, usually sampling villages in specific geographic settings. These studies found a strong spatial clustering of infection, but the association with SEP appeared to depend on the interlinkage between geography, economy, and occupational structure. In the *S*. *japonicum* surveillance sites of Hunan province, representing different geographic and epidemiologic conditions, strong village-level clustering of infection was observed, with higher infection levels in lake and embankment areas and among fishermen. Here, infection prevalence was higher in poorer villages (4.7%) than in richer ones (2.5%) (*p* < 0.001) (Fig 6) [28]. Conversely, in mountainous Eryuan county, the socioeconomically better-off plain areas with irrigated farmland had higher seroprevalence levels [26]. However, within the endemic (mostly plain area) villages of Eryuan, a higher SEP was protective against seroconversion (OR least poor versus poor: 0.48, 95% CI: 0.32–0.73) [25]. Similarly, two other papers report much greater odds of infection among illiterate and poor people within villages after adjusting for a range of intermediate variables such as water contact [27,37].

### S. mansoni

**Fig 7:**
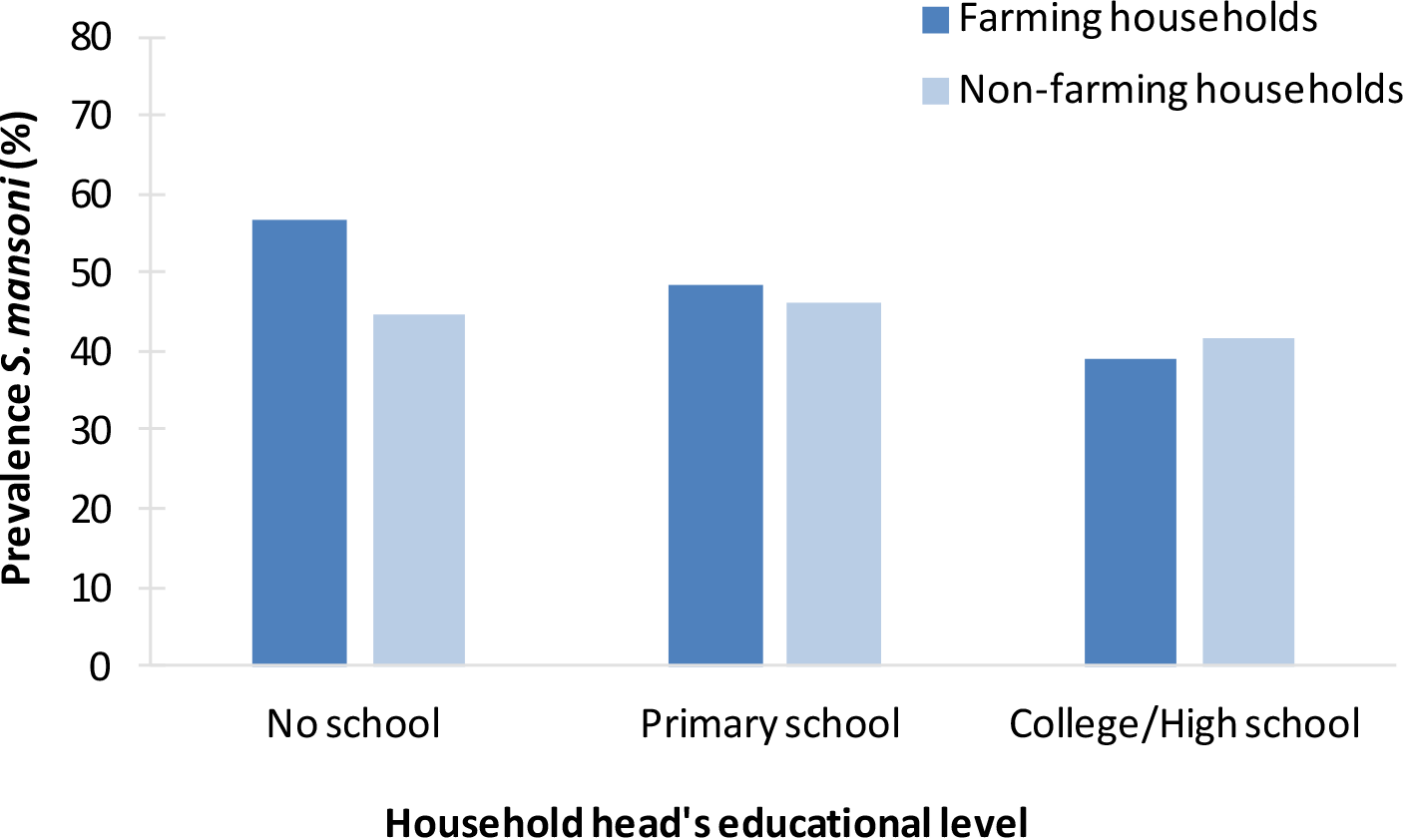
Example of socioeconomic inequalities in schistosomiasis: association between *S*. *mansoni* prevalence (%) and educational attainment of household head in the town of Man, western Côte d’Ivoire (2004–2005) [30]. The study was conducted in urban farming communities in the town of Man, western Côte d’Ivoire. A total of 113 farming households (586 individuals from all ages) and 21 nonfarming households (130 individuals from all ages) from six agricultural zones were interviewed, and stool samples were examined for *S*. *mansoni*. Infection prevalence was 51.4% in farming households and 44.6% in nonfarming households. Lower educational attainment was associated with higher infection prevalence in farming households (*p* = 0.008) but not in nonfarming households. Infection prevalence was higher in poorer households but not statistically significantly so.

Four studies among school children in Côte d’Ivoire—by the same authors using the same data—found similar odds of infection in all wealth groups except for lower odds among the least poor group [31–34]. A systematic socioeconomic gradient in infection prevalence was observed—with prevalences increasing from 39% among those with secondary education to 48% and 57% among those with primary education and no schooling, respectively (*p* = 0.01)—in a study among all age groups in Côte d’Ivoire. This association was found among farming households (irrigated rice cultivation) only; no association was found among nonfarming households (Fig 7) [30]. A study among school children in lakeside and island communities in Uganda reported a drop in the infection risk for each additional household asset owned (electricity, solar power, latrine, landline, mobile phone) (OR 0.74, *p* = 0.001) [35]. A very strong and systematic gradient in *S*. *mansoni* infection among teenagers was also found in a Ugandan study [36]. The odds of infection were 54.5 times higher in the poorest than in the richest households (next-poor: 15.9 times; next-rich: 3.5 times) (*p* < 0.001). This study also reported a systematic gradient in infection intensity, with a twice-as-high intensity in poor households compared with richer households. Substantial inequalities were also reported in a study among pregnant women in a secondary care hospital in central Sudan [29]. Here, the odds of infection were six times higher among women with no education than among those with secondary education or higher (OR 5.9, 95% CI: 2.8–12.3).

### S. haematobium

**Fig 8:**
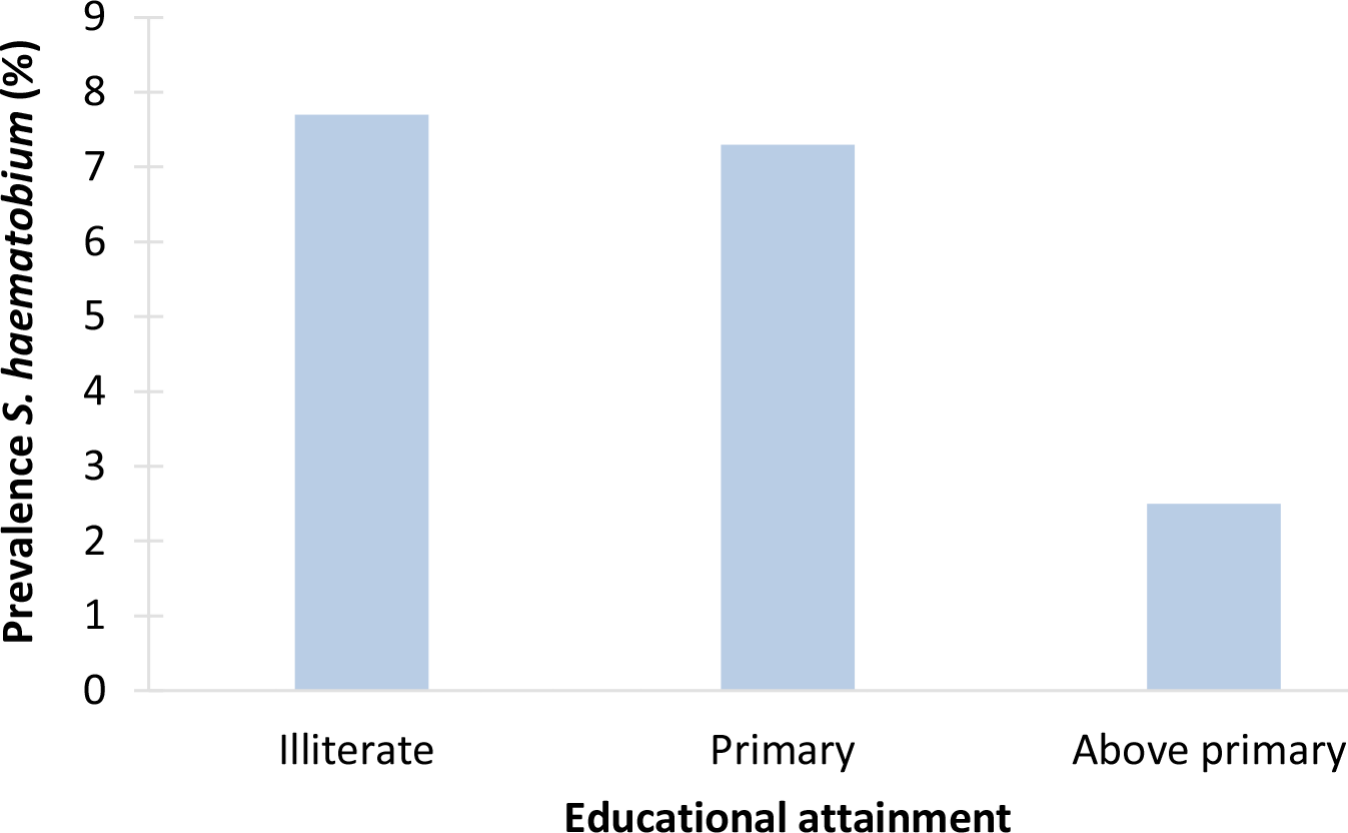
Example of socioeconomic inequalities in schistosomiasis: association between *S*. *haematobium* prevalence (%) and educational attainment in South Kordofan State, Sudan (2009) [6]. South Kordofan State is located in the south of Sudan, bordering South Sudan. A total of 1,826 adults (aged >18 years) were recruited from all nine localities (36 villages or towns) of South Kordofan State. Urine samples were examined for the presence of *S*. *haematobium*, and a questionnaire on demographics and economic status of the family head was administered. Overall prevalence of *S*. *haematobium* eggs was 6.9%. Infection prevalence was higher among people with a lower educational attainment.

The two studies on *S*. *haematobium* reported substantial inequalities in infection prevalence. In South Kordofan State, Sudan, the odds of infection were more than three times as high among adults with a low educational attainment (≤ primary school) than among those with a higher educational attainment (OR 3.07, 95% CI: 1.29–7.32) (Fig 8) [6]. In a study in Nigeria, the prevalence of overall and moderate or high infection (excreting >50 eggs/10 ml urine) was much higher among the poor than among the less poor (73% versus 1.5% for overall infection; 40% versus 0% for moderate or high infection, for monthly household incomes of <US$50 with ≥US$140, respectively). This study found no educational differences in overall infection prevalence and somewhat higher levels of moderate or high infection among the better educated but higher infection intensity among the less educated [23].

In sum, socioeconomic inequalities in schistosomiasis infection were usually (very) large. Nevertheless, the strength and direction of the association appears to be dependent on the intersection of geography and the occupational and socioeconomic structure of the study population. This is related to the strongly spatially clustered nature of the infection, which has to do with fishing and irrigated agriculture, among other causes.

### Soil-transmitted helminths (STH)

34 relevant papers on STH (*Ascaris lumbricoides*, *Trichuris trichiura*, hookworm disease) were found (China [GBD #1] four [25,37–39]; India [GBD #2] three [40–42]; Vietnam [GBD #5] five [43–47]; Malaysia [GBD #6] three [48–50]; Nigeria [GBD #8] three [51–53]; Brazil [GBD #9] ten [5,54–62]; Nepal [GBD #14] one [63]; Pakistan [GBD #15] one [64]; Ethiopia [GBD #16] one [65]; Colombia [GBD #18] one [66]; Tanzania [GBD #19] one [67]; Thailand [GBD #20] one) (S3 Table) [68]. In addition, we found two relevant studies (Ethiopia, Nigeria) about combined STH and schistosomiasis infection [69,70]. Two studies examined STH reinfection using a cohort design, and one paper reported on an ecological study; the other studies used a cross-sectional design. 18 studies included children, two included pregnant women or women of reproductive age, and 16 studies included all ages.

### Preschool- and school-aged children

**Fig 9:**
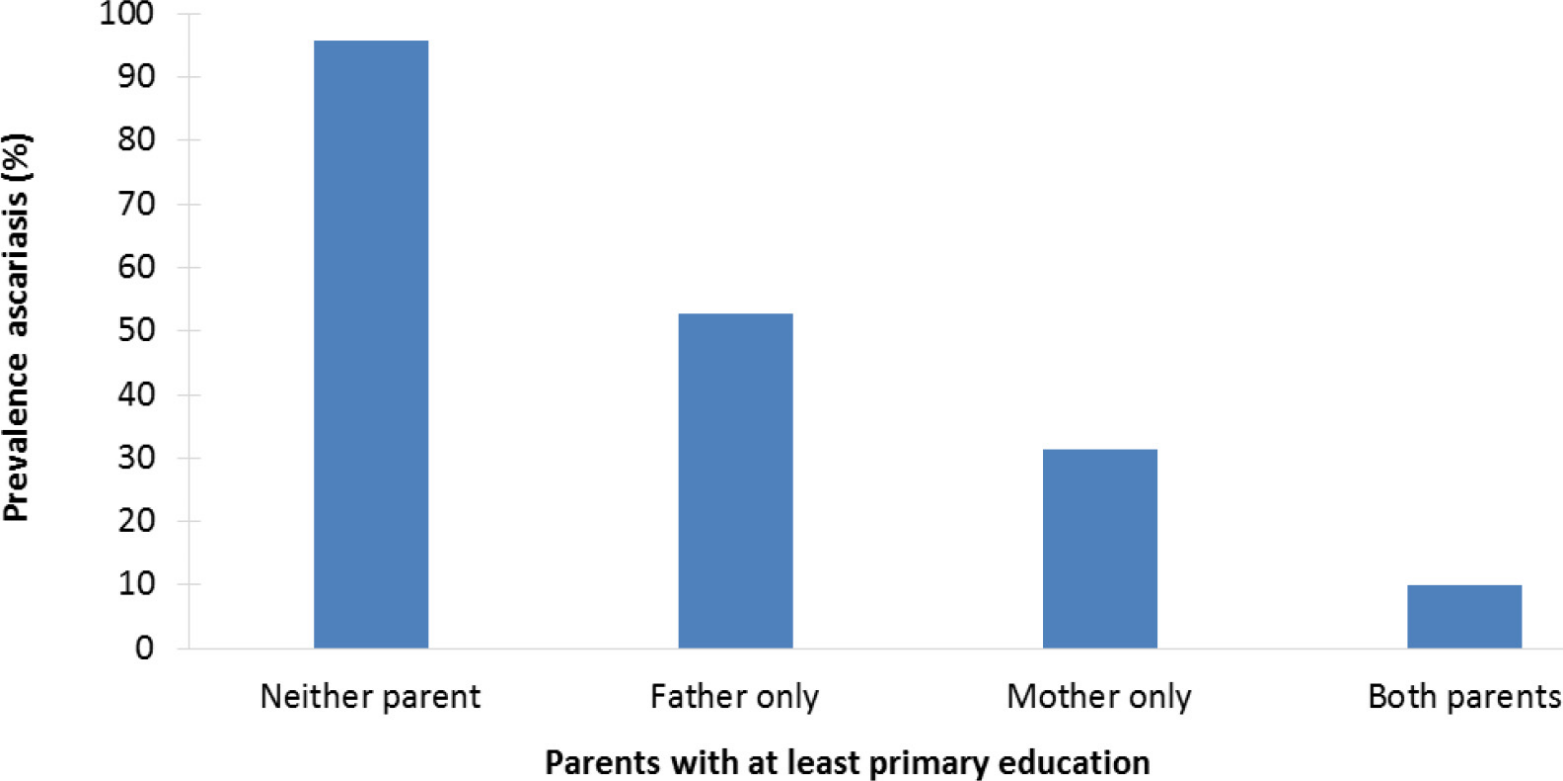
Example of socioeconomic inequalities in STH: parental education and ascariasis infection in a rural community in Osun State, Nigeria (2005–2006) [71]. A total of 440 children <16 years of age from randomly selected households were included. Information on parental education was collected through a questionnaire, and faecal samples were examined for the presence of *Ascaris* eggs. The prevalence of ascariasis was statistically significantly higher among children of parents without a primary education (*p* < 0.001).

STH prevalence was systematically and substantially higher among children from socioeconomically deprived households. Evidence comes from a range of settings (rural and urban areas in China, India, Pakistan, Nigeria, Brazil, and Malaysia) using a variety of SEP measures. While ORs varied strongly—from around 1.5 to 9—typically, infection prevalence was about twice as high among socioeconomically worse-off children. A comparatively large study in Brazil, for instance, found that 41% of poor children had any STH infection, compared with 22% of richer children [58]. Often, there was a systematic socioeconomic gradient in infection. A study in rural Nigeria, for example, found that ascariasis infection prevalence ranged from 10% when both parents had at least primary education to 31% when only the mother had such education and 53% when only the father had such education to 96% when neither parent had a primary education (Fig 9) [53]. Even in children under two years of age, STH prevalence was much higher among those from deprived backgrounds [52,66]. Ascariasis prevalence among children aged 0–25 months, for instance, was 27% among those whose father was a farmer, compared with 11%–13% among those whose father had a professional occupation or was a businessman [52]. Despite these inequalities, infection prevalence was sometimes also very high among better-off children. A study among schoolchildren in Kashmir, India, for instance, found that the prevalence of intestinal helminthiasis was 84% among children of illiterate mothers and 60% among children of mothers with secondary education [41].

There were minor variations to this overall pattern of inequality in STH prevalence in children. In rural Malaysian aboriginal children, the odds of infection were twice as high among poor children, but no association with parental education was found, nor was an association between household income and reinfection with any STH three and six months after treatment observed [48,49]. Conversely, among children attending outpatient clinics in Brazil, a higher prevalence of parasitic intestinal infection was associated with low parental educational attainment but not with income level [60]. A study among schoolchildren in Vietnam found no differences in parental income or education between children that were highly positive for ascariasis (egg per gram count (EPG) of >2,000) or trichuriasis (EPG of >600) and those without these infections [47].

### Other risk groups

The two studies among pregnant women (Thailand) and women of reproductive age (Vietnam) found strong associations between STH prevalence and SEP; the odds of infection were three to nine times higher among women from lower socioeconomic strata (Thai study: OR any STH 3.2 [95% CI, 2.0–5.3]; Vietnamese study OR any STH 7.5 [95% CI, 3.4–16.4], OR ascariasis 9.0 [95% CI, 3.6–22.7], OR trichuriasis 3.7 [95% CI, 1.5–9.1]).

In tea estates in Assam, India, STH prevalence was very strongly associated with educational attainment and occupational status. The prevalence of ascariasis, trichurariasis, and hookworm disease was 5%–6% among staff (teachers, health workers, factory workers) and ranged between 45%–52% among workers (tea-pickers) (*p* < 0.001). Similarly strong associations with educational attainment, in turn strongly associated with occupational status, were found [40].

### General population

Evidence from China, Brazil, and Malaysia indicates that also in the general population (all ages), the association between SEP and STH prevalence is often strong [5,50,54]. For example, in remote poor rural villages in five Malaysian states, the prevalence of intestinal parasitic infections was 83.5% among people from low-income households and 40% among richer people (*p* < 0.001) [50]. Findings were similar in a study on hookworm in southeastern Brazil, illustrating that, despite large inequalities, infection prevalence can remain high among the better off. A study in Hunan province, China, found that the odds of any STH infection were up to five times lower in wealthier households, after adjusting for a range of intermediate determinants such as hygienic behaviors [37]. Reinfection 12 months after successful treatment was found to be twice as high among the poor than among the least poor in a study in southeastern Brazil [56].

At the same time, the strength and direction of the association sometimes differed by type of parasite and SEP indicator [25,44–46,62,65]. For example, a study in an endemic urban area in Brazil found that household income was associated with hookworm infection (prevalence among those with no wage: 16%; >US$396: 5%, *p* = 0.005) but not with ascariasis or trichuriasis [62]. In mountainous Eryuan county, China, the odds of ascariasis infection were about twice as high among the poor and less educated, but trichuriasis was almost exclusively found in areas below 2,150 m, where richer people lived [25]. A Vietnamese study in an agricultural community where the intensely polluted Nhue Rivier was used for irrigation and where excreta were used as fertiliser found an association between STH infection and lower educational attainment but found no association with household economic status [44]. The authors hypothesize that this is due to the relatively homogeneous study population, but it is perhaps also related to the inclusion of rice fields and fish ponds—risk factors for STH—as indicator of economic status. A study among podoconiosis patients and healthy controls in Ethiopia found no association between educational attainment and STH [65].

### Combined STH and schistosomiasis infection

Two studies, one from Ethiopia [69] and one from Nigeria [70], measured the association between SEP and intestinal parasite status. The study among schoolchildren in Ethiopia found no association between family income and intestinal parasite infection (*A*. *lumbricoides*, *T*. *trichiura*, hookworm, *S*. *mansoni*). The Nigerian study, conducted among adult nomadic Fulanis, found that 95% of people with no schooling were infected with an intestinal parasite (*A*. *lumbricoides*, *T*. *trichiura*, and *S*. *mansoni*, among others) compared with a minority among those with some education [70]. Findings were similar when using housing type (hut, brick, cement) as SEP indicator.

In sum, socioeconomic inequalities in STH prevalence are often large, with ORs of two or greater, both among children and pregnant women or women of reproductive age and in the general population. Despite these inequalities, infection prevalence sometimes remained also high among better-off people. In several cases, the specific pattern depended on the measure of socioeconomic status used and the type of STH infection.

### Trachoma

11 relevant papers were found (Ethiopia [GBD #4] six [72–77]; Brazil [GBD #6] one [17]; Sudan [GBD #9] one [78]; Tanzania [GBD #11] three [79–81]) (S4 Table). All studies used a cross-sectional design, describing trachoma prevalence by SEP in specific age groups (especially young children) or the general population within a defined geographical area. MDA coverage was generally not reported.

**Fig 10:**
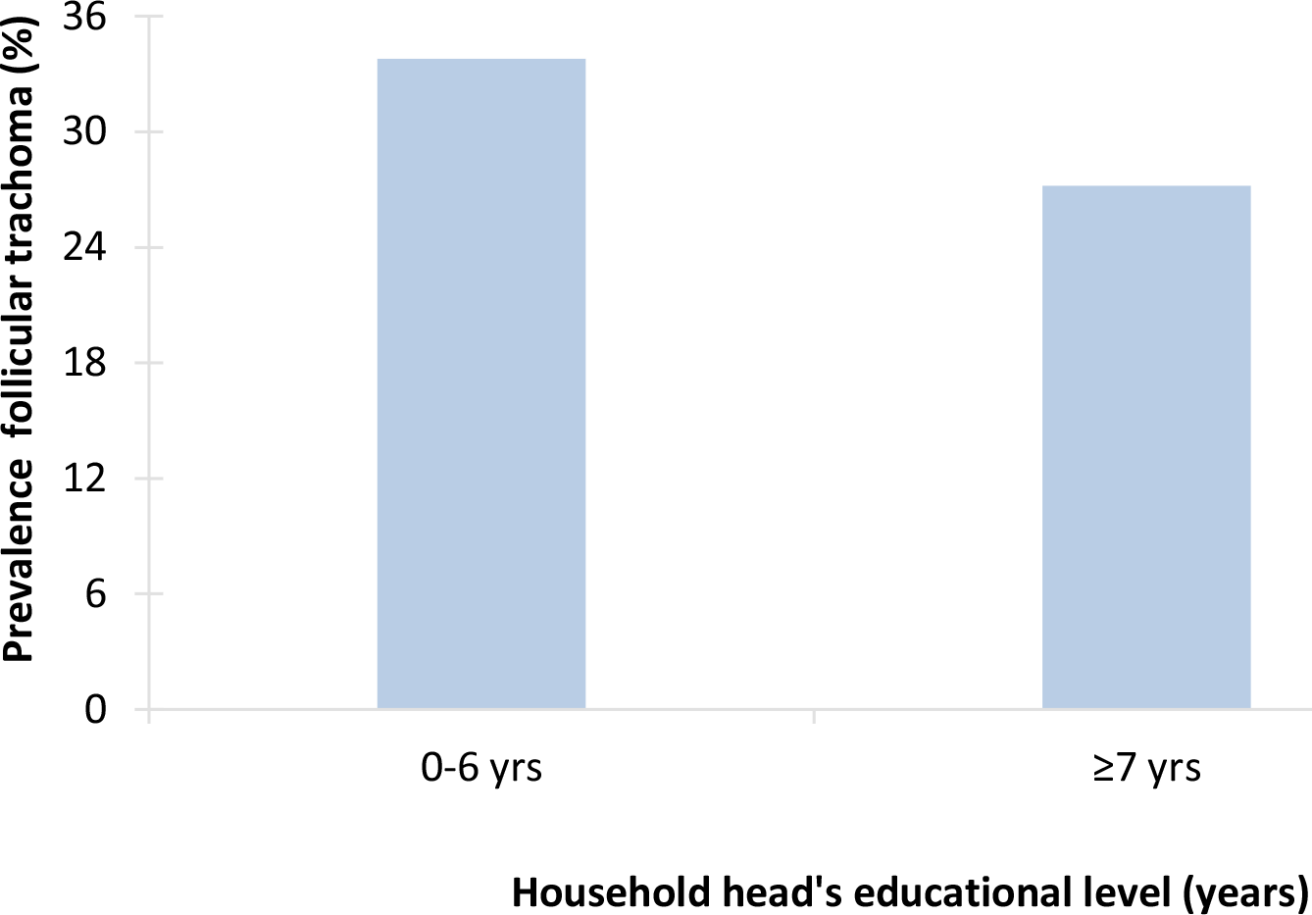
Example of socioeconomic inequalities in trachoma: association between educational attainment of household head and follicular trachoma in young children in Tanzania (2008) [79]. The study was conducted among children aged 0–5 years in 36 communities (3,122 children) in 2008. The eyes of the children were examined for active trachoma, and information on the educational attainment of the household head was collected through a questionnaire. The prevalence of follicular trachoma was 30.9%. Lower educational attainment of the household head was associated with a higher risk of follicular trachoma among children in both countries (Tanzania *p* = 0.001).

All studies examined the association between SEP and active trachoma (TF only, TF and/or TI), usually in young children. Seven studies (Ethiopia five, Tanzania two) reported statistically significant associations between SEP and the prevalence of active trachoma, with higher prevalences among lower strata (Fig 10) [73–77,79,80]. The strength of the association varied between and within (by the SEP indicator used) studies, from moderate (OR between 1.3–1.9) [74,75,79]) to strong (OR between 4 and 9) [73,75,76], with one study reporting a concentration index (CI) (-0.0942) instead of an OR. Two studies (Ethiopia, Sudan) reported associations in the same direction but with fairly wide confidence intervals, including the reference value [72,78]. One study reported no clear pattern between active trachoma and SEP [81].

Two papers also reported on the prevalence of ocular *Chlamydia trachomatis* infection, with similar findings on the association with SEP as for active trachoma [72,79]. One study (Ethiopia) also reported on TS and TT (> ten years of age) and found increasing socioeconomic inequalities between illiterate and literate people with increasing trachoma severity, with ORs rising from 1.9 (95% CI 1.65–2.26) for TF or TI to 2.57 (95% CI 2.1–3.2) for TS and 4.2 (95% CI 2.4–6.9) for TT [74]. Findings from another study on TT were in the same direction, but confidence intervals were wide, including the reference value [78].

One study (Brazil, all ages) compared the distribution of education and income between people with and without trachoma (any trachoma including *C*. *trachomatis* infection). People with trachoma were more likely to be less educated, but this pattern was not consistently statistically significant. Richer people in this study were more likely to have trachoma than poorer people, which the authors attributed to the association between age on the one hand and trachoma and wealth on the other, with older people being richer (in this study population) and having a higher likelihood of having trachoma [17].

In sum, the odds of trachoma in children and adults were usually substantially higher among poor and less-educated people and households. These socioeconomic inequalities possibly increase with increasing trachoma severity, but this finding could also be due to confounding by age, as more severe forms of trachoma are more common in older people who are also more likely to be illiterate. As trachoma prevalence rises with age, a positive association between income and trachoma prevalence may be observed in areas where older people are richer.

### Chagas’ disease

Four papers on Chagas’ disease met the inclusion criteria (Brazil [GDB #1] two [82,83]; Argentina [GDB #3] one [84]; Colombia [GDB #5] one [85]) (S5 Table). Two studies represented the general population in a specific geographical area using a cross-sectional design [82,84], while two focussed on pregnant women (cross-sectional) [85] or women who recently gave birth (case control) [83], reflecting the importance of vertical infection transmission.

The three studies that tested for anti-*Trypanosoma cruzi* seropositivity found strong and statistically significant associations between SEP and infection, with two to three times higher odds of infection among lower than among higher strata. A study among pregnant women in Colombia even reported an OR of 19.6 (95% CI 2.5–152.2) comparing women without completed primary education to university-educated women [85]. The Argentinian study compared self-reported Chagas and vector presence by SEP. In a region with horizontal control strategies, they found lower reported vector prevalence, higher self-reported Chagas prevalence, and smaller (and not statistically significant) educational inequalities in such prevalence than in a region with vertical control strategies [84]. The authors concluded that horizontal control strategies reduced vector prevalence, raised awareness of Chagas’ disease in all strata, and reduced inequalities in such awareness.

In sum, there is a paucity of evidence on socioeconomic inequalities in Chagas prevalence. The available evidence suggests that the odds of Chagas’ disease are much greater for lower socioeconomic groups than for higher strata, both in the general population and particularly in pregnant women.

### Human African trypanosomiasis (HAT)

No relevant papers were found.

### Leprosy

Seven papers on leprosy met the inclusion criteria (Brazil [GBD #2] six [7,8,86–91]; Bangladesh [GBD #7] one [92]) (S6 Table). Four studies represented the general population in a defined geographical area (two population-based or ecological studies [90,88], two case control studies [90,92]). Three studies focussed on specific groups: patients with no leprosy contacts (case-control study) [91], contacts of newly diagnosed leprosy patients (cohort study) [8], and past-five-year migrants (case-control study) [86].

The Brazilian studies all showed a strong association between SEP and leprosy, with a systematic gradient in studies comparing more than two strata. The odds of leprosy were at least twice as high in poorer and less-educated people as in the better off. When using water and sanitation facilities as proxy for (household or community level) SEP, the ORs varied strongly between studies, from 1.17 (95% CI 0.96–1.43) and 1.44 (95% CI 0.95–2.80) [91] to 3.1 (95% CI 1.1–10.02) [86]. The odds of being poor were nearly five times higher among leprosy patients than among controls [90]. Among coprevalent contacts of newly diagnosed leprosy patients, the odds of having leprosy were strongly associated with the educational attainment and income level of the patient. The association with SEP of the contact itself was weaker and not always statistically significant. After follow up of contacts, the odds of leprosy were not systematically associated with educational attainment or income level of the leprosy patient or the contact, except perhaps with a low income of the contact [8].

In the Bangladeshi study, the odds of leprosy were systematically higher among poorer people when using household assets as wealth indicator (borderline statistically significant) [92]. There was no association between income level and educational attainment on the one hand and the odds of leprosy on the other. Conversely, food shortage in the past year was associated with clinical signs of leprosy.

In sum, recent evidence on socioeconomic inequalities in leprosy prevalence is scarce. In Brazil, socioeconomic inequalities in leprosy were large. In Bangladesh, the association remains ambiguous. There is no evidence from other countries.

### Visceral leishmaniasis (VL)

17 papers on VL met the inclusion criteria (India [GBD #1] six [93–98]; Bangladesh [GBD #2] two [99,100]; Ethiopia [GBD #5] one [101]; Uganda and Kenya [GBD #11, #14] one [102]; Brazil [GBD #19] seven [103–108]) (S7 Table). The studies were typically conducted in (highly) endemic areas among the general population and all age groups. One study examined risk factors among people living in mud-wall houses [100], one compared migrants with residents [101], and one examined VL prevalence in household contacts of VL patients [106]. Four of the Brazilian studies were conducted in state capital cities. Study designs varied from ecological to cross-sectional, cohort, and case-control.

The ecological studies (two from India; three from Brazil) found moderate to strong associations between low SEP (poverty, unemployment, low educational attainment) and higher VL incidence [93,94,104,105]. The individual-level studies found that the risk of VL infection was as high or higher in lower strata than in the better off, although findings were not universally consistent across SEP indicators. Details about these individual-level studies are reported below.

The South Asian studies reported 1.6 to 3.4 times higher odds of VL in the poorest groups compared with the less poor [96–98,100]. One study also reported a 2.9 (95% CI 1.3–6.8) times higher odds of infection for the Mushahar caste, who are among the poorest of the poor, after adjusting for wealth quintile and a range of intermediate factors [98]. Two of these studies also examined literacy and found no association with VL infection [96,100]. Conversely, two other studies found no association between poverty and infection, while one of these also examined literacy and found a 1.66 times (95% CI 1.10–2.51) higher odds of VL infection among illiterate people [95,99]. One of these studies attributes the lack of association between poverty and VL to the homogenous high-risk study population [99]. Yet, even among people living in mud-wall houses in rural Bangladesh, the poorest villagers (without electricity) had a 3.40 (95% CI 1.76–6.59) higher odds of VL infection than less-poor villagers (with electricity) [100].

Among the Brazilian studies, two report moderate to strong associations between educational attainment and VL infection [103,107]. Conversely, another study reports no income differences between people with and without VL infection [108]. Among household members of VL patients, the risk of infection was not associated with income level, but it was statistically significantly lower among those owning many household assets [106].

The two African studies report large socioeconomic inequalities in VL. In a study population largely consisting of pastoralists in Uganda and Kenya, a very strong and systematic association between household wealth and VL was observed, with 13 times higher odds of infection in the poorest quintile and five times greater odds in the next-poor quintile than in the least poor group [102]. The Ethiopian study found a 1.4 to nearly 3 times higher odds of VL among less-educated and poor people and those with a low occupational status compared with the better off, but no association was found with land ownership.

In sum, most (15 out of 17) studies found a (much) higher odds of VL infection among lower socioeconomic strata along at least one indicator of SEP, but findings were not universally consistent across SEP indicators. Several studies found inequalities in VL along one SEP indicator but not along another. Only two studies found no association between SEP and VL.

## Discussion

Our systematic review exhibited a paucity of recent evidence on within-country socioeconomic inequalities in several NTDs—onchocerciasis, HAT, LF, and Chagas, in particular—even for high-burden countries. Yet, for STH, schistosomiasis, VL, and, to a lesser extent, for trachoma, there is considerable evidence of substantial socioeconomic inequalities in the odds of infection, with often (much) higher odds among socioeconomically disadvantaged groups. While the magnitude of inequality varied, the odds of infection were usually at least twice as high among disadvantaged groups compared with better-off people. Inequalities often took the form of a gradient in studies comparing more than two strata, with subsequently greater odds of infection each step down the socioeconomic hierarchy. Notwithstanding these inequalities, the prevalence of some NTDs remained high also among better-off groups in some highly endemic areas.

### Limitations of the study

#### Search strategy

The limited number of obtained studies might be partly a result of our search strategy, which only included international publications in the last decade and studies conducted in the 20 countries with the highest burden for each NTD. The rapid changes in many countries—in NTD prevalence [109], economic growth, and concurrent changes in inequality in income and educational attainment—underscore the importance of having up-to-date evidence. While the magnitude and pattern of socioeconomic inequalities in NTDs may have been different in earlier decades, this will be difficult to assess, given the incomparable methodologies used in the studies we reviewed (see below). The included countries comprised nearly 90% of the global burden for the studied NTDs (ranging from around 83%–86% for schistosomiasis and STH to >90% for LF, VL, and leprosy, 97.5% for trachoma, and 100% for Chagas, HAT, and onchocerciasis).

#### Publication and review bias

The substantial proportion of papers reporting inequalities in infection risk could be due to publication bias, with studies reporting statistically significant associations being more likely to get published. Yet, most studies were not specifically set up to assess inequalities, and the fact that we rarely found statistically significantly higher odds of infection among rich people supports the plausibility of our conclusions. Review bias may have led to the exclusion of studies finding no association between SEP and infection, as these did not always report a measure of association.

#### Methods used to assess the relationship between SEP and NTD prevalence in the original studies

Many of the included studies were not designed to describe socioeconomic inequalities in infection prevalence. Often, SEP was examined as one of a wide range of risk factors or only as a potential confounder, using a broad range of methods from a variety of disciplinary backgrounds. The SEP measures used were not always well described or well constructed, and the distribution of the study population across SEP categories was not always described. Sometimes, ORs were only provided after adjustment for intermediate variables (which generally leads to a downward bias in the magnitude of inequality), and potential confounders such as age were not always taken into account. Also, sampling design (clustering) was infrequently taken into account in the included studies, potentially leading to too narrow confidence intervals. Furthermore, studies were usually highly local in nature rather than taken from a nationally representative population sample. For these reasons, one cannot directly compare the magnitude of socioeconomic inequality in NTDs between studies. We will discuss the implications of two problems in more detail: potential confounding by age and sex and the highly local nature of many studies.

Third factors, especially age and sex, may sometimes have biased the reported magnitude of inequality. Inequalities may be overestimated for (more severe forms of) trachoma and Chagas, which are more common among elderly people, who are often less educated. This possibly contributed to the larger inequalities in more severe forms of trachoma than in less-severe forms of trachoma in an Ethiopian study [74]. Conversely, in some settings, older people tend to be richer, arguably explaining the finding of a Brazilian study that people with trachoma (mainly TS) were wealthier and less educated [17]. Nevertheless, studies of trachoma in children generally found substantial inequalities by household (head) SEP, and studies on Chagas that adjusted for age also reported large inequalities, suggesting that confounding by age does not explain the overall pattern of socioeconomic inequality in NTD prevalence. Conversely, some other infections like STH and schistosomiasis are more common among younger people, with usually a higher educational attainment, possibly leading to an underestimation of the magnitude of inequality.

The reported inequalities are probably an underestimation of inequalities within countries as a whole. Most studies were small and conducted in relatively homogeneous, high-risk populations and/or highly endemic areas, leaving country-level heterogeneity in wealth and infection risk underexposed. Several studies that found no or small inequalities cite study population homogeneity as explanation [92,99]. Nevertheless, even within small, highly endemic areas, inequalities were often substantial [26]. Furthermore, several studies that found no statistically significant association between SEP and infection lacked statistical power to detect substantial inequalities. Finally, the reported socioeconomic inequalities in overall prevalence of worm infections may only partly reflect inequalities in intensity of these infections. Most morbidity due to macroparasitic infections is directly associated with intensity of infection at the individual level and thus only indirectly with prevalence of infection at the community level. Infection intensity is known to strongly vary between individuals within communities [110]. While few papers report on the socioeconomic distribution of infection intensity, it is likely that reported inequalities in overall prevalence of worm infection hide even larger inequalities in intensity of infection. The same would arguably hold for multiple parasitic infections in the same individual and/or community.

### Explaining inequalities

Spatial clustering of infection because of geographic conditions, among other causes, is typical for most NTDs. While poor people conceivably tend to live in areas that are conducive to NTD transmission, studies unravelling the contribution of such conditions to socioeconomic inequalities in NTDs are rare. The relationship between spatial infection clustering and SEP may be context-specific, depending on the intersection of economy, geography, and occupational structure. A Chinese study, for instance, found higher schistosomiasis prevalence in richer plain areas with irrigated agriculture, but greater odds of infection among lower SEP groups within these richer areas [26].

Living conditions associated with poverty play an major role in NTD transmission and undoubtedly also in explaining the association between SEP and infection [3]. Scarce multivariate analyses indeed suggest that living conditions are important intermediates in the pathway between SEP and infection [36,38,46,74]. Furthermore, difficulties in accessing preventive and curative care increase the odds that poor people become infected and, once infected, are left untreated [3,111–113]. The costs of care can be substantial for, for example, treatment of trichiasis and advanced stages of Chagas. But even when care is free, other barriers, including distance, low quality of care, and other costs (for transport, working time forgone) can hamper early diagnosis and treatment, which is critical for the prevention of advanced stages of, for instance, leprosy, trachoma, and Chagas. At the same time, MDA for PCT NTDs has the potential to reduce infection prevalence across all socioeconomic layers by reducing barriers to treatment and through herd effects. Unfortunately, the coverage of NTD control programs was not systematically reported, limiting our ability to draw conclusions about their impact on inequalities in infection prevalence. Further research on the socioeconomic distribution of the coverage of NTD control programs will be important to understand the extent to which these efforts help reduce socioeconomic inequalities in NTDs.

SEP clearly influences the odds of NTD infection. The effects of infection on poverty and educational attainment in children are equally clear [4,114]. The paucity of cohort studies makes it difficult to unravel the relative importance of either direction of causality, and evidence from available cohort studies is inconsistent [8,48,56,100]. However, it is clear that reverse causation cannot fully explain inequalities in infection. Many studies report greater odds of infection in children of less-educated parents and greater odds of infection among less-educated adults, while in these cases reverse causation can hardly play a role.

### Implications for policy making and research

The public health impact of socioeconomic inequalities in NTDs is large. The short- and long-term consequences of STH infection, for instance, include anaemia in pregnant women and impaired nutritional status, growth, and cognitive development of children, and it has effects on school attendance and performance, with long-term consequences for health, educational attainment, productivity, and income levels [4,114]. Addressing these inequalities will contribute to more equal life prospects and freedom to lead a flourishing life.

Improving the social determinants of health—the conditions in which people grow, live, work, and age, and the structural drivers of these conditions—is critical for a sustainable reduction in socioeconomic inequalities in NTDs [3,36]. Thus, interventions to reduce inequalities in NTDs should include poverty reduction and improving educational attainment as well as improving housing, water, and sanitation conditions for poor people. Hence, action on inequalities in NTDs in not just the responsibility of the ministry of health and (international) organisations specialized in vector control and mass drug administration. Rather, it requires action across nongovernmental organisations and government departments, including, among others, public works, urban planning, agriculture, finance, and education [3,113].

Reducing inequalities in NTDs requires equitable access to preventive and curative health care without the risk of suffering from catastrophic health expenditures. While treatment of infection is free or inexpensive for NTDs, the costs of care can remain high for some, especially for more advanced disease stages [3], and are compounded by other costs and barriers to seeking care [3]. Universal health coverage for NTDs requires health system strengthening—the penetration of good quality care and vector control programs in poor and remote areas, reaching the poor where they live, with prevention, early detection, and affordable, quality treatment.

Action on inequalities in NTDs requires that monitoring and surveillance systems include equity indicators for disease prevalence and burden and the reach and impact of interventions. Such systems should include, at the minimum, disaggregated data by educational attainment and wealth quintile, further stratified by age and sex where relevant, with measures of statistical uncertainty. The emphasis on reducing inequalities in the Sustainable Development Goals can provide an impetus to the NTD community to incorporate indicators of socioeconomic position in routine monitoring and evaluation.

Evidence-based action would also benefit from more systematic equity research on NTDs to describe the socioeconomic distribution of infection—adjusted for confounders like age and sex—and to measure the contribution of intermediary determinants and the equity impact of interventions. Such research is hampered by the dependence on intensive field data collection because of the lack of national registration data for most NTDs in most countries. Prediction modelling has therefore become important in NTD research, and the evidence presented in our paper can be used to take socioeconomic heterogeneity into account in such models.

## Conclusion

While commonly seen as diseases of the poor, recent evidence on socioeconomic inequalities in several individual NTDs—in particular, onchocerciasis, HAT, LF, and Chagas—remains scarce, and more systematic research on the link between socioeconomic position and NTD infection is warranted. Yet, for some NTDs—in particular, STH, schistosomiasis, and VL—there is considerable evidence of substantially higher odds of infection among socioeconomically disadvantaged groups. Addressing these inequalities in NTDs will contribute to more equal life prospects and freedom to lead a flourishing life. NTD control activities, when set up such that they reach the most in need, will benefit the poorest populations within poor countries. This requires action across government departments and across nongovernmental organisations to improve the social determinants of health and ensure universal access to preventive and curative care. It is recommended that NTD monitoring and surveillance systems include equity indicators to help ensure that interventions reach the most in need.

**Key Learning Points**

- We found that evidence on the relationship between socioeconomic position and infection or disease prevalence remains scarce for several individual NTDs. Yet, for some NTDs—in particular, STH, schistosomiasis, and VL—there is considerable evidence that poor and less-educated people are at a much higher risk of getting the infection or disease than better-off people.
- The magnitude of this inequality varies, but the risk of infection or disease is often about twice as high among disadvantaged groups compared with better-off people. These inequalities often run across the entire society, with a subsequently higher risk of getting an NTD each step down the socioeconomic hierarchy.
- Notwithstanding these socioeconomic inequalities, the prevalence of some NTDs can also be high among the better off in some highly endemic areas, especially for STH.
- It is recommended that NTD monitoring and surveillance systems include equity indicators to help ensure that interventions reach the most in need.

**Top Five Papers**

1. World Health Organization. Investing to overcome the global impact of neglected tropical diseases: Third WHO report on neglected tropical diseases. Geneva: World Health Organization, 122015.
2. Aagaard-Hansen J, Chaignat CL. Neglected tropical diseases: equity and social determinants. In: Blas E, Sivasankara Kurup A, editors. Equity, social determinants and public health programmes Geneva: World Health Organization; 2010. p. 135–57.
3. Hotez PJ. The Disease Next Door. Foreign Policy. 2013;March 25.
4. Dunn C, Callahan K, Katabarwa M, Richards F, Hopkins D, Withers PC, Jr., et al. The Contributions of Onchocerciasis Control and Elimination Programs toward the Achievement of the Millennium Development Goals. PLoS Negl Trop Dis. 2015;9(5):e0003703. doi: 10.1371/journal.pntd.0003703. PubMed PMID: 25996946; PubMed Central PMCID: PMC4440802
5. Commission on Social Determinants of Health. Closing the gap in a generation: health equity through action on the social determinants of health. Final Report of the Commission on Social Determinants of Health. Geneva: World Health Organization. 2008.

## Supporting Information

S1 Supporting Information Systematic search protocol. (DOCX)

S2 Supporting Information PRISMA checklist. (DOCX)

S1 Table Summary of the literature on socioeconomic inequalities in LF, 2004–2013. (DOCX)

S2 Table Summary of the literature on socioeconomic inequalities in schistosomiasis, 2004–2013. (DOCX)

S3 Table Summary of the literature on socioeconomic inequalities in STH, 2004–2013. (DOCX)

S4 Table Summary of the literature on socioeconomic inequalities in trachoma, 2004–2013. (DOCX)

S5 Table Summary of the literature on socioeconomic inequalities in Chagas’ disease, 2004–2013. (DOCX)

S6 Table Summary of the literature on socioeconomic inequalities in leprosy, 2004–2013. (DOCX)

S7 Table Summary of the literature on socioeconomic inequalities in visceral leishmaniasis, 2004–2013. (DOCX)
