# Supplementary material for: Socioeconomic Inequalities in Neglected Tropical Diseases: A Systematic Review
Source: PLoS Negl Trop Dis. 2016 May 12;10(5):e0004546. doi: 10.1371/journal.pntd.0004546 (PMC4865383; doi:10.1371/journal.pntd.0004546)
Supplement: S1 Supporting Information — (DOCX) [file pntd.0004546.s001.docx]

**S1 Supporting Information. Systematic search protocol.**

The following research question was formulated: What is the distribution within countries, by socioeconomic position, of LF, onchocerciasis, schistosomiasis, STH, trachoma, Chagas’ disease, HAT, leprosy, and VL?

The search strategy was designed by a biomedical information specialist (GdJ) in cooperation with MCK. The basic search elements included the nine NTDs, epidemiology, and socioeconomic position. Each element was thoroughly translated into the controlled vocabulary of the databases (Emtree for Embase and Medical Subject Headings for Medline) and into free text words in title and/or abstract using a systemized process of optimization. A filter for human studies was added.

The following databases were searched: Embase.com (Embase and Medline), Medline (OvidSP), Cochrane Central, Web of Science, Popline, Lilacs, Scielo. Additionally, the most relevant results from Google Scholar were screened and we searched for articles present in PubMed but not yet available in Medline. The results were deduplicated in EndNote.

First-stage screening on the basis of title and abstract was done by HKK, who, when in doubt, discussed findings with MCK and TAJH, only excluding papers that were definitely not: about the NTD under study, about the association between SEP and the NTD, or within the above mentioned the study and publication period. In the second stage, the full text of the papers was reviewed by HKK, MCK, and TAJH, with at least 2 of the authors involved in the decision about in/exclusion. The inclusion critera were as follows:

- published in an international peer reviewed journal
- published between January 2004 and December 2013 (last 10 years)
- study period between 2000 and 2013
- top-20 country in terms of infection burden for the NTD under study
- reporting estimates of the relationship between socioeconomic position and NTD infection with a measure of statistical significance

We excluded papers that did not report the study period.

**Search strategies**

1. Search strategies for ten NTD's, socioeconomic positions, epidemiology (December 13^th^ 2013)

**Embase (Embase and Medline)**

((('African trypanosomiasis'/de OR 'Chagas’ disease'/de OR trypanosomiasis/de OR leishmaniasis/exp OR leprosy/exp OR 'Helminthiasis'/de OR 'Ascariasis'/de OR 'Trichuriasis'/de OR 'Hookworm infection'/exp OR 'Schistosomiasis'/exp OR 'Trachoma'/de OR 'Chlamydiasis'/de OR 'Dracunculiasis'/de OR 'Lymphatic filariasis'/exp OR 'Elephantiasis'/de OR 'Filariasis'/de OR 'Onchocerciasis'/de OR (((sleeping OR Hansen* OR neglected OR Robles) NEAR/3 (disease* OR sickness)) OR ((NTD* OR GWD) AND disease*) OR Chagas’* OR leishmaniasis OR trypanosomiasis OR ((leishmania OR trypanosom* OR schizotrypanum OR helminth* OR worm* OR ascaria* OR hookworm* OR trichuri* OR whipworm* OR ancylostoma* OR schistosom* OR dracuncul* OR filari* OR Brugia OR Onchocerca) AND (infect* OR infestation* OR disease*)) OR 'black fever' OR 'kala azar' OR lepros* OR lepra* OR helminthi* OR helmintho* OR ascarias* OR ascario* OR trichurias* OR trichocephal* OR bunostomias* OR schistosomias* OR schistosomat* OR schistosomios* OR bilharzi* OR 'Katayama fever' OR Trachoma* OR 'Egyptian ophthalmia' OR (chlamydia NEAR/3 conjunctiv*) OR dracunculi* OR dracunculo* OR draconti* OR filarias* OR philarias* OR wucher* OR brugias* OR elephantias* OR onchocercias* OR onchocercos* OR (river NEXT/1 blindness)):ab,ti) AND ('Epidemiological data'/exp OR 'Epidemiology'/exp OR 'Health statistics'/exp OR 'Cluster Analysis'/de OR 'Data analysis'/de OR 'Quality adjusted life year'/de OR (distribut* OR epidemiol* OR incidence* OR morbidit* OR survey* OR surveillance* OR endemic* OR hyperendemic OR ((disease* OR illness*) NEAR/3 (frequenc* OR burden* OR exposure*)) OR statistic* OR mortalit* OR prevalen* OR survival* OR (life NEXT/1 year*) OR DALY*):ab,ti)) OR 'leprosy epidemiology'/de) AND ('Social status'/exp OR 'Socioeconomics'/exp OR 'Social Justice'/de OR (((social* OR sociolog* OR economic*) NEAR/3 (status OR stratum OR strata OR level* OR position* OR condition* OR class* OR factor* OR justice)) OR socioeconomic* OR income* OR wealth* OR poor* OR 'non-poor' OR povert* OR impoveri* OR literac* OR read* OR writ* OR (education* NEAR/3 (level* OR status)) OR marginali* OR exclusion* OR exclud* OR vulnerab* OR inequ* OR disparit* OR equal* OR equit* OR ((living OR life) NEAR/3 (standard*))):ab,ti) NOT ([animals]/lim NOT [humans]/lim)

**Medline (OVID-SP)**

("Trypanosomiasis, African"/ OR exp "Chagas’ disease"/ OR "Trypanosomiasis"/ OR exp "Leishmaniasis"/ OR exp leprosy/ OR "Helminthiasis"/ OR "Ascariasis"/ OR "Trichuriasis"/ OR exp "Hookworm infection"/ OR exp "Schistosomiasis"/ OR "Trachoma"/ OR "Chlamydiaceae Infections"/ OR "Chlamydia Infections"/ OR "Dracunculiasis"/ OR "Elephantiasis, Filarial"/ OR "Elephantiasis"/ OR "Filariasis"/ OR exp "Onchocerciasis"/ OR (((sleeping OR Hansen* OR neglected OR Robles) ADJ3 (disease* OR sickness)) OR ((NTD* OR GWD) AND disease*) OR Chagas’* OR leishmaniasis OR trypanosomiasis OR ((leishmania OR trypanosom* OR schizotrypanum OR helminth* OR worm* OR ascaria* OR hookworm* OR trichuri* OR whipworm* OR ancylostoma* OR schistosom* OR dracuncul* OR filari* OR Brugia OR Onchocerca) AND (infect* OR infestation* OR disease*)) OR "black fever" OR "kala azar" OR lepros* OR lepra* OR helminthi* OR helmintho* OR ascarias* OR ascario* OR trichurias* OR trichocephal* OR bunostomias* OR schistosomias* OR schistosomat* OR schistosomios* OR bilharzi* OR "Katayama fever" OR Trachoma* OR "Egyptian ophthalmia" OR (chlamydia ADJ3 conjunctiv*) OR dracunculi* OR dracunculo* OR draconti* OR filarias* OR philarias* OR wucher* OR brugias* OR elephantias* OR onchocercias* OR onchocercos* OR (river ADJ blindness)).ab,ti.) AND ("Epidemiological monitoring"/ OR "Epidemiology"/ OR epidemiology.xs. OR Statistics.pt. OR "Cluster Analysis"/ OR "Data interpretation, Statistical"/ OR "Quality adjusted life years"/ OR (distribut* OR epidemiol* OR incidence* OR morbidit* OR survey* OR surveillance* OR endemic* OR hyperendemic OR ((disease* OR illness*) ADJ3 (frequenc* OR burden* OR exposure*)) OR statistic* OR mortalit* OR prevalen* OR survival* OR "disability adjusted life years" OR (life ADJ year*) OR DALY*).ab,ti.) AND (exp "Socioeconomic Factors"/ OR "Social Justice"/ OR (((social* OR sociolog* OR economic*) ADJ3 (status OR stratum OR strata OR level* OR position* OR class* OR factor* OR justice)) OR socioeconomic* OR income* OR wealth OR poor OR "non-poor" OR poverty OR impoveri* OR literacy OR read* OR writ* OR (education* ADJ3 (level* OR status)) OR marginali* OR exclusion* OR exclud* OR vulnerab* OR inequ* OR disparit* OR equal OR equit*).ab,ti.) NOT (animals NOT humans).sh.

**PubMed Medline as supplied by publisher**

(((sleeping[tiab] OR Hansen*[tiab] OR neglected[tiab] OR Robles[tiab] OR NTD*[tiab] OR GWD*[tiab]) AND (disease*[tiab] OR sickness[tiab])) OR Chagas’*[tiab] OR leishmaniasis[tiab] OR trypanosomiasis[tiab] OR ((leishmania[tiab] OR trypanosom*[tiab] OR schizotrypanum[tiab] OR helminth*[tiab] OR worm*[tiab] OR ascaria*[tiab] OR hookworm*[tiab] OR trichuri*[tiab] OR whipworm*[tiab] OR ancylostoma*[tiab] OR schistosom*[tiab] OR dracuncul*[tiab] OR filari*[tiab] OR Brugia[tiab] OR Onchocerca[tiab]) AND (infect*[tiab] OR infestation*[tiab] OR disease*[tiab])) OR black fever*[tiab] OR kala azar*[tiab] OR lepros*[tiab] OR lepra*[tiab] OR helminthi*[tiab] OR helmintho*[tiab] OR ascarias*[tiab] OR ascario*[tiab] OR trichurias*[tiab] OR trichocephal*[tiab] OR bunostomias*[tiab] OR schistosomias*[tiab] OR schistosomat*[tiab] OR schistosomios*[tiab] OR bilharzi*[tiab] OR Katayama fever*[tiab] OR Trachoma*[tiab] OR Egyptian ophthalmia*[tiab] OR (chlamydia[tiab] AND conjunctiv*[tiab]) OR dracunculi*[tiab] OR dracunculo*[tiab] OR draconti*[tiab] OR filarias*[tiab] OR wucher*[tiab] OR brugias*[tiab] OR elephantias*[tiab] OR onchocercias*[tiab] OR onchocercos*[tiab] OR river blindness*[tiab]) AND (((social*[tiab] OR sociolog*[tiab] OR economic*[tiab]) AND (status[tiab] OR stratum[tiab] OR strata[tiab] OR level*[tiab] OR position*[tiab] OR class*[tiab] OR factor*[tiab] OR justice[tiab])) OR socioeconomic*[tiab] OR income*[tiab] OR wealth*[tiab] OR poor*[tiab] OR non-poor*[tiab] OR povert*[tiab] OR impoveri*[tiab] OR literacy[tiab] OR read*[tiab] OR writ*[tiab] OR (education*[tiab] AND (level*[tiab] OR status[tiab])) OR marginali*[tiab] OR exclusion*[tiab] OR exclud*[tiab] OR vulnerab*[tiab] OR inequ*[tiab] OR disparit*[tiab] OR equal*[tiab] OR equit*[tiab]) AND (distribut*[tiab] OR epidemiol*[tiab] OR incidence*[tiab] OR morbidit*[tiab] OR survey*[tiab] OR surveillance*[tiab] OR endemic*[tiab] OR hyperendemic[tiab] OR ((disease*[tiab] OR illness*[tiab]) AND (frequenc*[tiab] OR burden*[tiab] OR exposure*[tiab])) OR statistic*[tiab] OR mortalit*[tiab] OR prevalen*[tiab] OR survival*[tiab] OR life year*[tiab] OR DALY*[tiab]) AND publisher[sb]

**Web of Science**

TS=((((sleeping OR Hansen* OR neglected OR Robles) NEAR/3 (disease* OR sickness)) OR ((NTD* OR GWD) AND disease*) OR Chagas’* OR leishmaniasis OR trypanosomiasis OR ((leishmania OR trypanosom* OR schizotrypanum OR helminth* OR worm* OR ascaria* OR hookworm* OR trichuri* OR whipworm* OR ancylostoma* OR schistosom* OR dracuncul* OR filari* OR Brugia OR Onchocerca) AND (infect* OR infestation* OR disease*)) OR "black fever" OR "kala azar" OR lepros* OR lepra* OR helminthi* OR helmintho* OR ascarias* OR ascario* OR trichurias* OR trichocephal* OR bunostomias* OR schistosomias* OR schistosomat* OR schistosomios* OR bilharzi* OR "Katayama fever" OR Trachoma* OR "Egyptian ophthalmia" OR (chlamydia NEAR/3 conjunctiv*) OR dracunculi* OR dracunculo* OR draconti* OR filarias* OR philarias* OR wucher* OR brugias* OR elephantias* OR onchocercias* OR onchocercos* OR (river NEAR/1 blindness)) AND (((social* OR sociolog* OR economic*) NEAR/3 (status OR stratum OR strata OR level* OR position* OR class* OR factor* OR justice)) OR socioeconomic* OR income* OR wealth* OR poor* OR "non-poor" OR povert* OR impoveri* OR literac* OR read* OR writ* OR (education* NEAR/3 (level* OR status)) OR marginali* OR exclusion* OR exclud* OR vulnerab* OR inequ* OR disparit* OR equal* OR equit*) AND (distribut* OR epidemiol* OR incidence* OR morbidit* OR survey* OR surveillance* OR endemic* OR hyperendemic OR ((disease* OR illness*) NEAR/3 (frequenc* OR burden* OR exposure*)) OR statistic* OR mortalit* OR prevalen* OR survival* OR (life NEAR/1 year*) OR DALY*))

**Popline**

(trypanosomiasis OR Chagas’ OR leprosy OR Helminthiasis OR Ascariasis OR Trichuriasis OR Hookworm OR Schistosomiasis OR Trachoma OR Chlamydiasis OR Dracunculiasis OR filariasis OR Onchocerciasis OR "neglected disease") AND ("socioeconomic status" OR equity OR poor) AND (distribution OR prevalence OR epidemiology)

**Scielo (Scielo.org)**

(trypanosomiasis OR Chagas’ OR leprosy OR Helminthiasis OR Ascariasis OR Trichuriasis OR Hookworm OR Schistosomiasis OR Trachoma OR Chlamydiasis OR Dracunculiasis OR filariasis OR Onchocerciasis OR "neglected disease") AND ("socioeconomic status" OR equity OR poor) AND (distribution OR prevalence OR epidemiology)

**Lilacs (Virtual Health Library)**

(trypanosomiasis OR Chagas’ OR leprosy OR Helminthiasis OR Ascariasis OR Trichuriasis OR Hookworm OR Schistosomiasis OR Trachoma OR Chlamydiasis OR Dracunculiasis OR filariasis OR Onchocerciasis OR "neglected disease") AND ("socioeconomic status" OR equity OR poor) AND (distribution OR prevalence OR epidemiology)

**Google Scholar (first 10 pages)**

(trypanosomiasis|Chagas’|leprosy|Helminthiasis|Ascariasis|Trichuriasis|Hookworm|Schistosomiasis|Trachoma|Chlamydiasis|Dracunculiasis|filariasis|Onchocerciasis|"neglected disease") ("socioeconomic status"|equity|poor) (distribution|prevalence|epidemiology)
