# Supplementary material for: Socioeconomic Inequalities in Neglected Tropical Diseases: A Systematic Review
Source: PLoS Negl Trop Dis. 2016 May 12;10(5):e0004546. doi: 10.1371/journal.pntd.0004546 (PMC4865383; doi:10.1371/journal.pntd.0004546)
Supplement: S1 Table — (DOCX) [file pntd.0004546.s003.docx]

**S1 Table. Summary of the literature on socioeconomic inequalities in LF, 2004-2013.**

| **Top 20 GBD 2010;**  **Author, Year** | **Aim of study** | **Outcome,**  **detection method** | **Study design, statistical method, sample size** | **Study sample (period, area, population, age, randomization)** | **Measure of SEP** | **Strata** | **Prevalence**  %  (N inf/total N) | **Univariate association**  OR (95% CI), p-value | **Multivariate association**  OR (95% CI)  **(Adjusted for…)** |
| --- | --- | --- | --- | --- | --- | --- | --- | --- | --- |
| #1 India;  Upadhyayula SM *et al.*, 2012 | To assess the relationship between socioeconomic status and LF prevalence | Prevalance *Wuchereria Bancrofti* microfilaria;  Finger prick blood using microscope | Cross-sectional design;  Logistic regression;  N=5,394 | 2004-2007;  30 endemic villages in Karimnagar district, Andhra Pradesh, India;  MDA program since 2004, still ongoing; 58% of study population participated in MDA program.  Villagers;  All ages;  40 households per village were randomly selected (5 people per household) | Monthly income (US$)^[[1]](#endnote-1)^  Education | <22 US$  22-66 US$  >66 US$  Undergraduate  Graduate | (total N)  4.9% (1,251)  3.5% (3,382)  2.6% (761)  3.8% (5,065)  2.4% (329)  Overall prevalence: 3.7% | 1.9 (1.1-3.2)  1.3 (0.8-2.2)  1 (ref), p=0.02  1.6 (0.8-3.2), p=0.22  1 (ref) | 2.6 (1.1-6.5), p<0.05  1 (ref)  (Age, house structure, drainage system, participated in MDA program) |
| #1 India;  Vijay Kumar *et al.*, 2008 | To explore the usage and costs of personal-protection measures for LF and long-term trends in LF prevalence across income groups | Prevalance *Wuchereria Bancrofti* microfilaria;  Finger prick blood | Cross-sectional design;  Chi-square;  N=7,560 people from 1,660 households | 2004-2006;  Chennai, capital of Tamil Nadu state, India; MDA status not reported;  Household members;  All ages;  Different areas of the city were classified by income level before randomly selecting households | Locality-based income measure^[[2]](#endnote-2)^ | Very Low  Low  Moderate | (N localities, total N)  Mean prevalence  1.30%  (18, 3,220)  1.15%  (11, 1,998)  0.51%  (25, 2,342)  Overall prevalence: 0.90% | At locality level:  p=0.01 | NR |
| #11 Tanzania;  Mwakitalu ME *et al.*, Acta Trop 2013 | To investigate LF infection, disease and transmission in a medium-sized city after seven rounds of mass drug administration | Prevalence of circulating filarial antigens (CFA);  Finger prick blood using ICT cards | Cross-sectional design;  Chi-square test;  N=960 children from 3 wards | 2012;  3 wards in the city of Tanga, Eastern Tanzania;  School children;  5-16 yrs;  2 urban and 1 peri-urban wards, representative for the city in terms of population density and socioeconomic and environmental characteristics: 1 or 2 neighboring public primary schools were selected from each of the three wards | Household ownership of TV or fridge (individual level and ward level measure, N=895) | (% owning TV/fridge)  Tangasisi (35%/19%)  Makorora (52%/31%)  Central (73%/52%)  Tangasisi + Makorora  Central | 6.9% (22/320)  6.3% (20/317)  3.4% (11/323)  6.6%  3.4%  Overall prevalence: 5.5% | At individual level: p>0.05  At ward level (comparing Tangasisi/Makorora vs. Central): p=0.04 | NR |
|  |  |  | Cross-sectional design  Chi-square test;  N=806 people from 3 wards | Community members;  59% of respondents participated in one or more of seven MDAs.  ≥10 yrs;  1 ‘ten cell unit’ (smallest administrative unit) from which a high number of the examined pupils came was selected from each ward, but because many community members refused to participate 1–2 neighbouring units were also selected | Household monthly income below <48 US$^c^  (N=142) | (% below <48 US$)  Tangasisi (81%)  Makorora (82%)  Central (70%) | 13.2% (42/317)  14.2% (41/288)  19.9% (40/201)  Overall prevalence: 15.3% | NR | On ward level: p>0.05  (Sex, age, as the sex and age distributions varied strongly between the wards) |
| #11 Tanzania;  Mwakitalu ME *et al.*, Parasit Vectors 2013 | To investigate epidemiological aspects of LF as background for planning and implementation of control measures | Prevalence of circulating filarial antigens (CFA);  Finger prick blood using ICT cards | Cross-sectional design;  Chi-square test;  N=1,697 children from 6 wards | 2011;  Ilala district, Dar es Salaam, eastern Tanzania; MDA was implemented twice (2006, 2007) in the city;  Schoolchildren;  5-13 yrs  6 out of 24 wards were selected based on distance to city center, population density, environmental characteristics, and facilities: 1 or 2 public primary schools were selected from each of the 6 wards; | Household ownership of TV or fridge (N=1,496) | (% owning TV/fridge)  Chanika (34%/16%)  Majohe (40%/26%)  Vingunguti (56%/23%)  Mchikichini (68%/49%)  Ukonga (69%/46%)  Buguruni (72%/50%)  Vingunguti and Majohe  Buguruni, Ukonga and Mchikichini  (Chanika was not included in this comparison. The authors comment that it did not fit the pattern, perhaps because of its rural location.) | 1.9% (5/552)  4.5% (12/700)  5.3% (16/782)  2.5% (7/347)  1.0% (3/692)  2.4% (7/582)  5.0%  2.1%  Overall prevalence: 3.0% | At individual level:  p>0.05  At ward level:  p=0.01 | NR |
|  |  |  | Cross-sectional design;  Chi-square test;  N=1,212 people from 4 wards | Community members; 19% participated in at least 1 of the 2 MDA campaigns;  ≥10 yrs;  1 ‘ten cell unit’ (smallest administrative unit) from which a high number of the examined pupils came was selected from 4 wards, but because many community members refused to participate 1–2 neighboring units were also selected | Household monthly income below <48 US$^[[3]](#endnote-3)^  (N=172) | (% below <48 US$)  Vingunguti (82%)  Chanika (74%)  Mchikichini (47%)  Ukonga (41%)  Vingunguti  Chanika, Mchikichini and Ukonga | 13.5% (47/348)  8.8% (25/284)  7.0% (20/287) 7.8% (23/293)  13.5%  7.9%  Overall prevalence: 9.5% | At ward level: p<0.01 | NR |

inf: infected. LF: Lymphatic Filariasis. MDA: mass drug administration

1. The paper reported: <1000 Rs, 1000-3000 Rs and >3000 Rs, currency rate used: 1 Indian Rupee = 0.022 US$, January 1, 2006 (mid of study period, www.xe.com). [↑](#endnote-ref-1)
2. The paper does not provide a definition. [↑](#endnote-ref-2)
3. The paper reported: <80,000 TSh and ≥80,000 TSh, currency rate used: 1 Tanzanian Shilling = 0.0006 US$, July 1, 2011 and January 1, 2012 (mid of study periods, both same rate, www.xe.com). [↑](#endnote-ref-3)
