# Supplementary material for: Socioeconomic Inequalities in Neglected Tropical Diseases: A Systematic Review
Source: PLoS Negl Trop Dis. 2016 May 12;10(5):e0004546. doi: 10.1371/journal.pntd.0004546 (PMC4865383; doi:10.1371/journal.pntd.0004546)
Supplement: S2 Table — (DOCX) [file pntd.0004546.s004.docx]

**S2 Table: Summary of the literature on socioeconomic inequalities in schistosomiasis, 2004-2013.**

| **Top 20 GBD 2010;**  **Author, Year** | **Aim of study** | **Outcome,**  **detection method** | **Study design, statistical method, sample size** | **Study sample (period, area, population, age, randomization)** | **Measure of SEP** | **Strata** | **Prevalence**  %  (N inf/total N) | **Univariate association**  OR (95% CI) | **Multivariate association**  OR (95% CI)  **(Adjusted for…)** |
| --- | --- | --- | --- | --- | --- | --- | --- | --- | --- |
| #1 Nigeria;  Ugbomoiko US *et al.*, 2010 | To explore the impact of knowledge and various demographic, socioeconomic, cultural and environmental factors on the prevalence and intensity of urinary schistosomiasis | *S.haemato-bium* infection (total and moderate-heavy infections: >50 eggs/10 ml urine);  Urine samples were examined using a light microscope | Cross-sectional design;  Logistic regression;  N=1,023 | 2006-2007;  Peri-urban and rural villages of Eko-ende and Ore, Osun state, south-western Nigeria;  Villagers;  All ages;  Households with members who had lived at least 4 months in Eko-ende or Ore were randomly selected and all household members were included | Education household head  Monthly household income (US$)^[[1]](#endnote-1)^  House ownership | Incomplete primary  Complete primary  <50 US$  50-139 US$  ≥140 US$  No  Yes | Mean (SD) intensity (eggs/10 ml urine); prevalence  all, moderate-heavy inf  131.4 (382.7);  61.0%, 27.0% (393, 174/644)  84.8 (199.3);  63.5%, 35.6%  (241, 135 /379)  168.6 (404.2);  72.8%, 40.4% (456, 253/626)  33.7 (90.6);  53.4%, 16.9%  (177, 56/331)  0.4 (3.2);  1.5%, 0%  (1, 0/66)  190.2 (423.4); 72.9%, 42.6%  (406, 296/173)  64.1 (232.1);  54.7%, 22.0%  (617, 338/136)  Overall prevalence: all 62.0% | Mean intensity; all, moderate-heavy inf  p=0.027;  p=0.42, p=0.004  p<0.001;  both p<0.001  p<0.001;  both p<0.001 | All, moderate-heavy inf  Illiterate: 1 (ref)  Literate: 0.28 (0.19-0.42), 0.30 (0.19-0.46),  both p<0.001  <500 US$^[[2]](#endnote-2)^: 3.73 (2.66-5.21), 3.35, 2.12-5.30, both p<0.001  >500 US$: 1 (ref)  1 (ref)^[[3]](#endnote-3)^  2.46 (1.75-3.46), 2.19 (1.50-3.18), both p<0.001  (Being single, no. of children 1-15 yrs/household, not living with biological parents, household close to the river) |
| #2 China;  Balen J *et al.*, 2011 | To examine the association between helminth infection and behavioural, demographic, economic, environmental, and social risk factors in two endemic settings | *S.japonicum* infection;  Stool samples were examined by Kato-Katz thick smear method | Cross-sectional design;  Logistic regression;  N=1,298 | 2006;  Wuyi (rural) and Laogang (peri-urban) villages, Dongting Lake region of Hunan province, China;  Villagers;  All ages;  Villages were selected on the basis of previous studies and ongoing collaboration with local anti-schistosomiasis control station personnel | Wealth quartiles^[[4]](#endnote-4)^ | Most poor  Below average  Above average  Most wealthy  N infected/total N: <=6 yrs 39/650, >6yrs 21/270 | Overall prevalence: 6.5% | NR | 1 (ref)  0.74 (0.60-1.99), p=0.17  0.13 (0.08-0.35), p<0.002  0.06 (0.02-0.19), p<0.001  (Village, occupation, lake water contact, soil contact, animal ownership, water source, washing hands with soap after defecation/before eating) |
| #2 China;  Peng WX *et al.*, 2010 | To identify areas of high and low risk of schistosomiasis within a community in a highly endemic area of China, and the factors influencing small-scale spatial variation | *S.japonicum* infection;  Stool samples were examined using Kato-Katz thick smear method | Cross-sectional design;  Logistic regression;  N=920 | 2007;  Ximiao village, Poyang Lake region, Jiangxi province, China;  Villagers;  5-80 yrs;  Sampling NR | Education (years) | ≤6 yrs  >6 yrs | 6.0% (39/650)  7.8% (21/270)  Overall prevalence: 6.5% | 0.78 (0.44-1.31)  1 (ref) | 1.22 (0.65-2.30)  1 (ref)  (Gender, age, occupation, distance of household to snail habitat) |
| #2 China;  Steinmann P *et al.*, Acta Tropica 2007 ^[[5]](#endnote-5),^ ^[[6]](#endnote-6)^ | To assess prevalence of schistosomiasis, STH and food-borne helminthes and to investigate behavioral, demographic, environmental and socioeconomic risk factors for infection | *S.japonicum* infection (based on stool samples);  Stool samples were examined using Kato-Katz method | Cross-sectional design;  Logistic regression;  N=3,220 from 35 villages. Risk factor analyses were only done for the 13 endemic villages, N=1,429 individuals) | 2005;  Eryuan county, north-west Yunnan province, southern China;  Household members;  ≥5 yrs;  35 villages were randomly selected from a map using a grid and 35 families per village were randomly selected, all family members ≥5 yrs were included | Education  Wealth quintiles^[[7]](#endnote-7)^ | Illiterate  ≤ junior middle school  ≥ high middle school  Most poor  Very poor  Poor  Less poor  Least poor | Overall prevalence: 2.7% (eggs) | 1 (ref)  0.95 (0.35-2.58), p=0.93  2.04 (0.47-8.76), p=0.34  1 (ref)  N.A  0.72 (0.16-3.29), p=0.67 0.86 (0.24-3.13), p=0.82 1.21 (0.35-4.13), p=0.77 | Education and Household asset index were not included |
|  |  | *S.japonicum*  infection (based on blood samples);  Blood samples were examined using ELISA |  |  | Education  Wealth quintiles | Illiterate  ≤ junior middle school  ≥ high middle school  Most poor  Very poor  Poor  Less poor  Least poor | Overall prevalence: 49.5% | 1 (ref)  1.10 (0.81-1.49), p=0.53 0.65 (0.37-1.15), p=0.14  1 (ref)  NA  0.71 (0.44-1.14), p=0.16  0.54 (0.35-0.82), p=0.004 0.48 (0.32-0.73), p=0.001 | 1 (ref)  1.58 (0.95-2.62), p=0.08  NA  0.68 (0.48-0.98), p=0.04  0.51 (0.36-0.73), p<0.001  (Consumption beef/fish, age, gender, ethnic group, tobacco grower) |
| #2 China;  Steinmann P *et al.*, Geospatial Health 2007^f^ | To develop a spatially-explicit statistical model by integrating epidemiological and remotely sensed environmental data for risk profiling of schistosomiasis in a mountainous area in China; and to explore the potential of serological data for the appreciation of infection pressure in this setting, where praziquantel has been repeatedly  administered over the past several years | *S.japonicum* seropositivityin **all** villages;  Blood samples were examined using ELISA | Cross-sectional design;  Bayesian non-spatial and spatial logistic  regression analyses adjusting for family and village level random effects;  N=3,220 from 35 villages | 2005;  Eryuan county, northwest Yunnan province, China;  Household members;  ≥5 yrs;  35 villages were randomly selected from a map using a grid and 35 families per village were randomly selected; all family members ≥5 yrs were included | Education  Wealth quintiles | Illiterate  ≤ junior middle school  ≥ high middle school  Most poor  Very poor  Poor  Less poor  Least poor | Overall prevalence: 27.1% | 1 (ref)  2.06 (1.69-2.51), p<0.001 1.55 (0.99-2.42), p=0.05  1 (ref)  3.47 (2.35-5.12), p<0.001  3.95 (2.69-5.79), p<0.001  7.77 (5.38-11.22), p<0.001  8.41 (5.85-12.10), p<0.001 | Non-spatial model:  1 (ref)  0.99 (0.57-1.59)  0.69 (0.39-1.12)  0.76 (0.42-1.26)  0.59 (0.32-0.99)  Spatial model (taking spatial correlation into account):  1 (ref)  1.37 (0.73-2.34)  1.04 (0.52-1.86)  1.10 (0.54-2.00)  0.79 (0.38-1.46)  (Gender, age, ethnic group, tobacco grower, Tenancy of irrigated agricultural land, slope, village location, median night-time land surface temperature, median normalized difference vegetation index) |
|  |  | *S.japonicum* seropositivityin schistome **endemic** villages; | N=NR |  |  | Illiterate  ≤ junior middle school  ≥ high middle school  Most poor  Very poor  Poor  Less poor  Least poor | Overall prevalence: 49.5% | 1 (ref)  1.10 (0.81-1.49), p=0.53  0.65 (0.37-1.15), p=0.14  1 (ref)  NA  0.71 (0.44-1.14), p=0.16  0.54 (0.35-0.82), p=0.004  0.48 (0.32-0.73), p=0.001 |  |
|  |  | *S.japonicum* seropositivityin **non-endemic** villages; | N=NR |  |  | Illiterate  ≤ junior middle school  ≥ high middle school  Most poor  Very poor  Poor  Less poor  Least poor | Overall prevalence: 9.2% | 1 (ref)  1.31 (0.92-1.87), p=0.14  1.16 (0.44-3.04), p=0.77  1 (ref)  1.51 (0.96-2.36), p=0.08  0.98 (0.59-1.63), p=0.95  1.95 (1.17-3.25), p=0.01  2.10 (1.21-3.65), p=0.01 |  |
| #2 China;  Xu J-F *et al.*, 2013 | To identify risk factors for schistosomiasis in order to offer guidelines for controlling the transmission tailored to the local settings | *S.japonicum*  seropositivity;  Blood samples were examined using IHA reconfirmed by stool samples (Kato-Katz method) | Cross-sectional design;  Logistic regression;  N= 2,339 from 1,247 households | 2010;  Villages in Jiangling County, Hubei Province, China;  Villagers;  6-60 yrs;  6 villages were randomly sampled by stratified clustered sampling in which prevalence and village were taken as stratum and cluster, respectively. | Educational level  Wealth tertiles^[[8]](#endnote-8)^ | Illiterate  Literate  Poor  General  Good | Overall prevalence: 15.1% | p=0.048  p<0.001 | 1 (ref)  0.45 (0.36-0.58)  1 (ref)  0.36 (0.28-0.47)  0.27 (0.19-0.37)  (Village, age, infection times, history of water contact) |
| #2 China;  Yang J *et al.*, 2009 | To explore risk factors for schistosomiasis in China | Infection status of *S.japonicum* ;  Blood samples were examined using IHA and reconfirmed by stool samples (Kato-Katz method) | Cross-sectional design;  Single-level and multi-level logistic regression;  N=10,245 | 2005;  16 villages^[[9]](#endnote-9)^ in Hunan province, China;  Villagers;  ≥6 yrs;  16 villages representing various geographic areas with different snail habitat types and severity of schistosomiasis (lake-embankment type, lake-beach type, inside embankment type, hills). | Village level per capita income (US$, N=10,108)^[[10]](#endnote-10)^ | <362 US$  ≥362 US$ | 4.7% (302/6,424)  2.5% (92/3,684)  Overall prevalence: 4.1% | p<0.001 | Single-level model:  1 (ref)  0.70 (0.56-0.88)  Multi-level model: income level was not included but reported as not significant.  (Gender, age, occupation, endemic type, drinking water sources, proportion of using hygienic lavatory, average precipitation in April, October and June, density of infected snail) |
| #5 Sudan;  Abou-Zeid AH *et al.*, 2012 | To estimate prevalence of schistosomiasis infection and to identify associated risk factors among the adult population to guide treatment strategies | *S. haemato-bium* infected*;*  Urine samples were examined | Cross-sectional design;  Logistic regression;  N=1,826 | 2009;  Kordofan State, Sudan;  Villagers;  >18 yrs;  Multi-stage random sampling^[[11]](#endnote-11)^; | Education | Illiterate  Primary  Above primary | 7.7% (77/994)  7.3% (43/591)  2.7% (6/241)  Overall prevalence: 6.9% | 3.29 (1.42-7.64)  3.07 (1.29-7.32)  1 (ref)  p=0.01 | Education was not included |
| #5 Sudan;  Khalid A *et al.*, 2012 | To investigate epidemiology of schistosomiasis infection among pregnant women in a  secondary-care hospital | *S.mansoni* infection*;*  Stool samples were examined using Kato-Katz method | Cross-sectional design;  Logistic regression;  N=292 | 2010;  Geizera state, central Sudan;  Pregnant women;  16-40 yrs;  Pregnant women attending prenatal care (in a secondary care-hospital)^[[12]](#endnote-12)^ | Education | None  ≥ Secondary | (25/87)  (13/205)  Overall prevalence: 13.0% | 5.9 (2.8-12.3), p<0.001  1 (ref) | 6.2 (2.8-12.9), p<0.001  1 (ref)  (Age, gravidity, gestational age, housewife) |
| #14 Côte d'Ivoire;  Matthys B *et al.*, 2007 | To identify risk factors for schistosomiasis and hookworm infections in urban farming communities, and to investigate small-scale spatial patterns of infection prevalence | *S.Mansoni* infection among **farming** households*;* | Cross-sectional design;  Bayesian spatial and non-spatial multiple logistic regression: last model considered between-household variation;  N=716 from 134 households | 2004-2005;  Town of Man, western Côte d’Ivoire;  Urban farming and non-farming households;  All ages;  131 farming households were identified by directly contacting farmers during their fieldwork; 34 households not engaged in urban agriculture were randomly selected  from the same study area using the EPI survey approach | Education household head  Wealth quintiles^[[13]](#endnote-13)^ | No school  Primary school  College/high school  Poorest  Very poor  Poor  Less poor  Least poor | 56.6% (180/318)  48.3% (86/178)  38.9% (35/90)  55.2% (80/145)  57.6% (83/144)  51.5% (51/99)  44.8% (43/96)  43.1% (44/102) | p=0.01  p=0.11 | *Results combined for farming and non-farming household*  Bayesian spatial model:  1 (ref)  0.69 (0.40-1.10)  0.37 (0.19-0.63)  1 (ref)  1.94 (0.97-3.52)  1.07 (0.50-2.01)  1.24 (0.57-2.39)  0.93 (0.42-1.78)  Non-spatial model:  1 (ref)  0.72 (0.50-1.04)  0.49 (0.30-0.79), p=0.01  1 (ref)  1.11 (0.69-1.76)  0.86 (0.52-1.44)  0.66 (0.39-1.11)  0.62 (0.37-1.03), p=0.11  (Gender, age, number of persons living in household, toilet disposal, distance to river, bathing/swimming in river, fishing with a net, contact with water from irrigation well or pond, cultivated crop type) |
|  |  | *S.Mansoni* infection among **non-farming** households*;*  Stool samples were examined using FECT and Kato-Katz method |  |  | Education household head  Wealth quintiles^[[14]](#endnote-14)^ | No school  Primary school  College/high school  Poorest  Very poor  Poor  Less poor  Least poor | 44.6% (25/56)  46.0% (23/50)  41.7% (10/24)  0% (0/0)  66.7% (2/3)  42.5% (17/40)  45.7% (21/46)  43.9% (18/41) | p=0.94  p=0.90 |  |
| #14 Côte d'Ivoire;  Raso G *et al.*, 2006^[[15]](#endnote-15)^ | To identify demographic, environmental, and socioeconomic risk factors for schistosomiasis and hookworm (co)infection in schoolchildren | *S.Mansoni* infection;  Stool samples were examined using Kato-Katz and SAF methods | Cross-sectional design;  Bayesian spatial regression: binomial and multinomial models with random effects;  N=3,578 | 2001-2002;  Man region, western Cote d'Ivoire;  Schoolchildren;  6-16 yrs;  Only children who were present at the 57 rural schools were screened | Wealth quintiles | Most poor  Very poor  Poor  Less poor  Least poor  Most poor  Very poor  Poor  Less poor  Least poor  Most poor  Very poor  Poor  Less poor  Least poor | Total prevalence: mono-infection 24.1%, coinfection with hookworm 19.0% | NR | Binomial spatial model:  1 (ref)  1.12 (0.85-1.44)  1.08 (0.82-1.39)  0.91 (0.69-1.18)  0.73 (0.55-0.95)  (Age, gender, elevation (m))  Multinominal spatial model:  RRR, 95% CI  *Mono-infection:*  1 (ref)  0.86 (0.61-1.21)  0.84 (0.61-1.17)  0.82 (0.59-1.14)  0.58 (0.41-0.82)  *Coinfection with hookworm:*  1 (ref)  1.33 (0.94-1.88)  0.89 (0.63-1.25)  0.59 (0.41-0.84)  0.41 (0.29-0.60)  (Age, gender, elevation (m), land cover type) |
| #14 Côte d'Ivoire;  Raso G *et al.*, Parasitol 2005^o^ | To identify risk factors explaining the geographical distribution of schistosomiasis infection in a mountainous region | *S. mansoni* infection;  Stool samples were examined using Kato-Katz method | Cross-sectional design;  Logistic regression (non-spatial) and Bayesian logistic spatial regression;  N=3,818 | 2001-2002;  Region of Man, western Côte d’Ivoire;  Schoolchildren;  6-16 yrs;  All schools located in the town of Man and those schools in rural areas with <100 pupils registered were excluded from the survey. In the remaining 57 rural schools, all schoolchildren attending grades 3–5 were enrolled for parasitological screening | Wealth quintiles^[[16]](#endnote-16)^ | Most poor  Very poor  Poor  Less poor  Least poor  Most poor  Very poor  Poor  Less poor  Least poor | Total prevalence: 38.9% | Non-spatial model:  1 (ref)  1.08 (0.88-1.33)  1.10 (0.89-1.35)  0.99 (0.80-1.22)  0.89 (0.72-1.10)  Spatial model:  1 (ref)  1.12 (0.86-1.44)  1.12 (0.86-1.43)  0.93 (0.70-1.19)  0.73 (0.55-0.95) | Non-spatial model:  1 (ref)  1.06 (0.84-1.31)  1.07 (0.86-1.33)  0.97 (0.78-1.20)  0.72 (0.58-0.89)  Spatial model:  1 (ref)  1.12 (0.85-1.44)  1.08 (0.82-1.39)  0.91 (0.69-1.18)  0.73 (0.55-0.95)  (*3 spatial models were given in the paper and here is the one presented with the best fit*)  (Age, gender, elevation (m)) |
| #14 Côte d'Ivoire;  Raso G *et al.*, Trop Med 2005^o^ | To investigate disparities in parasitic infections, schoolchildren’s self-reported ill health and access to health care in relation to the children’s socioeconomic status and to examine associations between parasitic infections and individual household assets, hand washing habits and place of residency | *S.mansoni*  infection;  Stool samples were examined using Kato-Katz method | Cross-sectional design;  Logistic regression;  N=3,374 | 2001-2002;  Region of Man, western Côte d’Ivoire;  Schoolchildren;  6-16 yrs;  All schools located in the town of Man and those schools in rural areas with <100 pupils registered were excluded from the survey. In the remaining 57 rural schools, all schoolchildren attending grades 3–5 were enrolled for parasitological screening | Wealth quintiles^[[17]](#endnote-17)^  Having bicycle  Having fan | Poorest  Very poor  Poor  Less poor  Least poor  No  Yes  No  Yes | (total N)  39.2% (676)  41.2% (663)  40.8% (660)  38.7% (679)  34.1% (696)  NR  NR  Overall prevalence: 38.7% | p>0.05 | 1 (ref)  1.42 (1.17-1.72), p<0.001;  1 (ref)  0.70 (0.53-0.93), p=0.01  (Age, gender, having soap, washing hands after defecation, living in village) |
| #14 Côte d'Ivoire;  Vounatsou P *et al.*, 2009^o^ | To map and predict the distribution of schistosomiasis prevalence data | Density ratio of excreted egg counts of *S. mansoni;*  Stool samples were examined using Kato-Katz method | Cross-sectional design;  Bayesian non-stationary zero-inflated negative binomial analysis which take village-specific spatial  random effects into account  N=3,818 | 2002;  Region of Man, western Côte d’Ivoire;  Schoolchildren;  Grade 3-5;  From 55 schools, 43 schools were randomly selected  and fitted into the models and the remaining 12 schools were used for validation purposes | Wealth quintiles^[[18]](#endnote-18)^ | Most poor  Very poor  Poor  Less poor  Least poor | Overall prevalence: 38.9% | NR | Density ratio of excreted egg counts (95% CI)  1 (ref)  1.05 (0.85-1.28)  0.98 (0.79-1.20)  1.16 (0.93-1.44)  1.15 (0.91-1.43)  (Age, gender, household within village boundary, elevation, stream order, watershed, normalized difference vegetation index, land cover, distance to permanent/temporary water bodies, mean land surface temperature, distance to dispensaries) |
| #15 Uganda;  Kabatereine NB *et al.*, 2011 | To examine  risk factors associated with intestinal schistosomiasis, STH and malaria, and to evaluate the prevalence of these three infections and to ascertain the prevalence of co-infection | *S.mansoni*  infection;  Stool samples were examined using Kato-Katz thick smear | Cross-sectional design;  Logistic regression including village-level random effects;  N=4,534 | 2009-2010;  Lake Victoria Islands, Uganda;  Schoolchildren;  10-14 yrs;  The Lot Quality Assurance Sampling protocol was followed: 203 villages, and 15 children, with a roughly even mix of gender and age, were randomly selected from each village | Enrolled in school  Education household head  Wealth index^[[19]](#endnote-19)^ | No  Yes  Continuous | Overall prevalence: 40.8% | 0.74, p<0.001  (every additional household asset item owned was associated with a decrease in the odds of infection ) | 1 (ref)  2.93 (2.11-4.06), p<0.001  1.10^[[20]](#endnote-20)^ (1.04-1.17), p=0.003  (Gender, age, hookworm infection, trichuris infection, household latrine present, enrolled in school and source of household water) |
| #15 Uganda;  Muhumuza S *et al.*, 2009 | To examine the association between socioeconomic position and the risk and intensity of infection with schistosomiasis | *S.Mansoni*  infection;  Stool samples were examined using Kato-Katz method | Cross-sectional design;  Logistic regression adjusting for clustering at village level;  N=463 | 2007;  Walukuba division, Jinja district, Uganda;  Teenagers;  10-20 yrs;  A random sample of about 20 homes was selected from each village from a list of all households using a table of random numbers.  One individual in the same household was randomly selected from those in the age group 10–20 years^[[21]](#endnote-21)^ | Education household head  Wealth quintiles^[[22]](#endnote-22)^  Wealth quintiles | None  Lower primary  Upper primary  Secondary  Tertiary  Lowest  Second  Third  Highest  Lowest  Second  Third  Highest | (96/108)  (77/92)  (70/96)  (50/126)  (8/41)  (105/110)  (98/114)  (61/106)  (37/133)  N inf  105  98  61  37  Overall prevalence: 65.0% | 33.0 (11.3-100.6), p<0.001  21.2 (7.5-62.1), p<0.001  11.1 (4.2-30.2), p<0.001  2.7 (1.1-7.0), p=0.03  1 (ref)  54.5 (19.4-165.4), p<0.001 15.9 (7.9-32.2), p<0.001 3.5 (2.0-6.3), p<0.001  1 (ref)  Infection intensity (geometric mean of egg counts/gram of faeces)  230.04 (189.71-278.93)  228.86 (187.71-279.13)  167.38 (127.50-219.74)  114.26 (80.33-162.11)  P<0.001 | 10.4 (3.4-32.3), p<0.001  5.4 (2.2-13.3), p<0.001  2.8 (1.3-6.1), p=0.01  1 (ref)  (Reported contact with water of lake Victoria in the past one week, total duration of contact with water of lake Victoria water in previous week, taken praziquantel during last distribution) |

NR: Not Reported; NA: Not Applicable; inf: infected; STH: Soil-transmitted Helminths (ascariasis (*A.lumbricoides*), trichuriasis (*T.trichuria*) and hookworm infection (*A.duodenale* and *N.Americanus))*; IHA: indirect hemagglutination test; FECT: formalin-ether concentration technique; SAF: sodium–acetic acid–formalin method; WASH: water supply, sanitation, and hygiene.

1. The paper reports household monthly income as <7500 naira, 7500-20,000 naira and ≥21,000 naira; exchange rate as used in our paper: 1 50 naira = 1 US$. [↑](#endnote-ref-1)
2. 500 US$ seems too high and might be an error in the paper. [↑](#endnote-ref-2)
3. Possibly, the reference group was reversed in the paper, but this remains unclear. [↑](#endnote-ref-3)
4. Household based asset index, constructed using principal components analysis. [↑](#endnote-ref-4)
5. This paper is also included in S6 Table on STH . [↑](#endnote-ref-5)
6. Both articles from Steinmann P *et al.* report about the same study population, but Steinmann P *et al.*, Geospatial Health includes also non-endemic villages.

   ^§^ These three papers by Raso G *et al.* and the paper by Vounatsou P et al. report about the same study population. [↑](#endnote-ref-6)
7. Based on household assets (radio, TV, telephone, video compact disc, electric fan, electric rice cooker, washing machine, refrigerator, bicycle, motorbike, car, tractor, no. of cows owned, m^2^ irrigated land for agriculture). [↑](#endnote-ref-7)
8. Based on household economic status, including (i) electric appliance, e.g. electric rice-cooker, electric fan, TV set, refrigerator, (ii) agricultural machine, e.g. tractor, cultivator, and (iii) vehicles, e.g. bicycle, motorcycle, automobile. [↑](#endnote-ref-8)
9. 16 villages represented various geographic areas with different snail habitat types and severity of schistosomiasis. These areas were divided into 4 defined types: lake-embankment type, lake-beach type, inside embankment type, and hill type. All citizens ≥6 yrs were invited to participate. [↑](#endnote-ref-9)
10. The paper reports: <3000 Yuan and ≥3000 Yuan; currency rate we used: 1 Chinese Yuan = 0.1208 US$, July 1, 2005 (mid of study period, www.xe.com). [↑](#endnote-ref-10)
11. From each locality, two administrative units were randomly selected, two villages/towns were randomly selected, which made a total of 36 villages/towns. Within each village/town ten families were included based on systematic random sampling. [↑](#endnote-ref-11)
12. Pregnant women attending prenatal care (in a secondary care-hospital) for the first time were invited to participate. Women with diabetes mellitus or hypertensive disorders of pregnancy were excluded. [↑](#endnote-ref-12)
13. Based on housing characteristics (e.g. type of wall) and household assets owned (e.g. bicycle). [↑](#endnote-ref-13)
14. Based on housing characteristics (e.g. type of wall) and household assets owned (e.g. bicycle). [↑](#endnote-ref-14)
15. These three papers by Raso G *et al.* and the paper by Vounatsou P et al. report about the same study population. [↑](#endnote-ref-15)
16. Based on 12 household assets: cement house, electricity, radio, television, refrigerator, fan, car, motorbike, bicycle, soap, sleeps under bed net, wears shoes. [↑](#endnote-ref-16)
17. Based on 12 household assets: cement house, electricity, radio, television, refrigerator, fan, car, motorbike, bicycle, soap, sleeps under bed net, wears shoes. [↑](#endnote-ref-17)
18. Based on assets owned (e.g., possession of a radio) and household characteristics (e.g., walls constructed with bricks). [↑](#endnote-ref-18)
19. Based on household assets: electricity, solar power, latrine, landline and mobile phone. [↑](#endnote-ref-19)
20. 1.10 increase for each additional level of educational attainment (no education, primary incomplete, primary complete, secondary incomplete, secondary complete or higher). [↑](#endnote-ref-20)
21. If there was no one in the 10 to 20 year age group or if such a person failed to provide a stool sample, the next household was included. [↑](#endnote-ref-21)
22. Based on type of housing, water supply, fecal disposal facilities and on household assets. [↑](#endnote-ref-22)
