# Supplementary material for: Socioeconomic Inequalities in Neglected Tropical Diseases: A Systematic Review
Source: PLoS Negl Trop Dis. 2016 May 12;10(5):e0004546. doi: 10.1371/journal.pntd.0004546 (PMC4865383; doi:10.1371/journal.pntd.0004546)
Supplement: S3 Table — (DOCX) [file pntd.0004546.s005.docx]

**S3 Table: Summary of the literature on socioeconomic inequalities in STH, 2004-2013.**

| **Top 20 GBD 2010;**  **Author, Year** | **Aim of study** | **Outcome,**  **detection method** | **Study design, statistical method, sample size** | **Study sample (period, area, population, age, randomization)** | **Measure of SEP** | **Strata** | **Prevalence**  %  (N inf/total N) | **Univariate association**  OR (95% CI) | **Multivariate association**  OR (95% CI)  **(Adjusted for…)** |
| --- | --- | --- | --- | --- | --- | --- | --- | --- | --- |
| #1, China;  Balen J *et al.*, 2011^[[1]](#endnote-1)^ | To examine the association of STH infection with behavioral, demographic, economic, environmental and social risk factors | Prevalence any STH infection;  Stool samples were examined using Kato-Katz thick smear method | Cross-sectional design;  Logistic regression;  N=1,298 | 2006;  Wuyi (rural) and Laogang (peri-urban) villages, Dongting Lake region, Hunan province, China;  Villagers;  All ages;  Selection of villages was based on previous epidemiological studies and ongoing collaborations; all citizens of these villages were invited to participate | Household asset index (quartiles)^[[2]](#endnote-2)^ | Most poor  Below average  Above average  Most wealthy | Overall prevalence: 8.2% (ascariasis 5.5%, trichuriasis 3.0%, hookworm infection 0.8%) | NR | 1 (ref)  0.68 (0.34-0.79), p=0.02  0.24 (0.13-0.61), p<0.001  0.18 (0.08-0.50), p<0.001  (Village, soil contact, animal ownership, washing hands with soap after defecation/before eating) |

| #1, China;  Steinmann P *et al.*, Acta Tropica 2007 ^[[3]](#endnote-3)^ | To assess prevalence of *S.japonicum*, STH and food-borne helminthes and to investigate behavioral, demographic, environmental and socio-economic risk factors for infection | Prevalence ascariasis; | Cross-sectional design;  Logistic regression;  N=3,220 people from 35 villages | 2005;  Eryuan county, north-west Yunnan province, southern China;  Household members;  ≥5 yrs;  35 villages were randomly selected from a map using a grid and 35 families per village were randomly selected, all family members ≥5 yrs were included | Education  (only for  ≥18 yrs, N=2,549)  Household asset index (quintiles)^[[4]](#endnote-4)^ | Illiterate  ≤ junior middle school  ≥ high middle school  Most poor  Very poor  Poor  Less poor  Least poor | Overall prevalence: 15.4% | 1 (ref)  0.76 (0.61-0.96), p=0.02  0.47 (0.24-0.92), p=0.03  1 (ref)  0.80 (0.59-1.08), p=0.14  0.61 (0.45-0.82), p=0.001  0.56 (0.41-0.75), p<0.001  0.51 (0.38-0.68), p<0.001 | 1 (ref)  0.76 (0.56-1.05), p=0.09  0.55 (0.39-0.77), p<0.001  0.51 (0.35-0.73), p<0.001  0.51 (0.34-0.75), p=0.001  (Washing hands before meals, consumption of raw pork/water plants, livestock breeder, temporary employment, village location) |
| --- | --- | --- | --- | --- | --- | --- | --- | --- | --- |
|  |  | Prevalence trichuriasis;  Stool samples were examined using Kato-Katz method |  |  | Education  (only for  ≥18 yrs, N=2,549)  Household asset index (quintiles) | Illiterate  ≤ junior middle school  ≥ high middle school  Most poor  Very poor  Poor  Less poor  Least poor | Overall prevalence: 1.7% | 1 (ref)  1.20 (0.60-2.36), p=0.61  NA  1 (ref)  3.73 (1.05-13.29), p=0.04  4.28 (1.23-14.86), p=0.02  3.00 (0.84-10.70), p=0.09  2.44 (0.68-8.78), p=0.17 | Education and household asset index were not included  (Washing hands before meals, consumption of raw pork/beef/vegetables, livestock breeder, resident at altitude >=2150m) |
| #1, China;  Wang X *et al.*, 2012 | To determine the prevalence and correlates of STH infections | Prevalence any STH infection;  Stool samples were examined using Kato-Katz method | Cross-sectional design;  Logistic regression and decom-position analysis;  N=1,701 children from 95 villages | 2010;  Rural Guizhou and Sichuan provinces, southwestern China;  Pre-school and schoolchildren;  3-5 yrs and 8-10 yrs;  Sampling details provided underneath the table.^[[5]](#endnote-5)^ | Maternal education:  ≥ secondary school  Paternal education:  ≥ secondary school | No  Yes  No  Yes | (258/935)  (118/766)  (190/712)  (186/989)  Overall prevalence ≥1 STH infection: pre-school children 21.2%, school children 22.9% | 1(ref)  0.48 (0.37-0.61), p<0.001  1(ref)  0.64 (0.51-0.80), p<0.001 | 1 (ref)  0.63 (0.48-0.83), p<0.001  1 (ref)  0.90 (0.67-1.17, p=0.44  (Age, gender, # family members, # siblings, washing hands before meals/after defecation, eating raw meat/vegetables, drinking unboiled water, dirty floor, household own toilet, soil-based latrine, use of human fecal material in household crop production, household owns livestock, use of human fecal material in household garden) |
| #1, China;  Zhang L *et al.*, 2013^[[6]](#endnote-6)^ | To report on the relationship between deworming treatment and STH infection in rural China and to identify the individual and household characteristics correlated with the decision to deworm | Any STH infection;  Stool samples were examined using Kato-Katz method | Cross-sectional design:  Probit regression;  N=1,724 children from 94 villages | 2010;  Guizhou province, southwest China;  Pre-school and schoolchildren;  3-5 yrs and 8-10 yrs;  6 rural counties were randomly selected from the bottom quartile of counties based on average net per capita incomes.^[[7]](#endnote-7)^ | Household wealth (quartiles)^[[8]](#endnote-8)^  Maternal education: ≥secondary school  Paternal education: ≥secondary school | 1  2  3  4  No  Yes  No  Yes | Overall prevalence any STH: 37.5% (pre-school children 32.6%, school children 42.3%) | Probit estimate  (ref)  -0.16  -0.23, p<0.05  -0.35, p<0.01  (Adjusted for taking deworming medicine during past 18 months) | Probit estimate  (ref)  -0.15  -0.17  -0.24, p<0.05  (ref)  -0.09, p>0.05  (ref)  -0.20, p<0.05  (Taking deworming medicine during past 18 months, age, student stage, student*age, gender, number of siblings) |
| #2, India;  Traub RJ *et al.*, 2004 | To determine the prevalence and risk factors of STHs among 3 tea-growing communities | Prevalence ascariasis | Cross-sectional design;  Logistic regression;  N=328 | 2000;  Rural Assam, north-east India;  Tea-growing community members;  All ages;  Households were randomly chosen from each housing division within each tea estate | Education  SES (based on status within tea estate) | None  Primary  Secondary  Tertiary  Worker  Staff | 48.6%  34.2%  34.2%  13.8%  6.5%^[[9]](#endnote-9)^  45.0%  Overall prevalence: 38% | p=0.04  p<0.001 | (Education was entered as a continuous variable)  0.78, 0.61-1.01, p=0.06  SES was not included^[[10]](#endnote-10)^  (Age, household crowding, anthelmintic usage, religion) |
|  |  | Prevalence trichuriasis |  |  | Education  SES (based on status within tea estate) | None  Primary  Secondary  Tertiary  Worker  Staff | 57.0%  45.5%  32.9%  6.9%  52.5%  4.8%  Overall prevalence: 43% | p<0.001  p<0.001 | 0.71, 0.55-0.93, p=0.01  SES was not included  (Household crowding, footwear outdoors, defecation site (indoor/outdoor), water source) |
|  |  | Prevalence hookworm infection;  Stool samples were examined using Kato-Katz method |  |  | Education  SES (based on status within tea estate) | None  Primary  Secondary  Tertiary  Worker  Staff | 65.4%  48.1%  34.2%  3.5%  52.0%  4.8%  Overall prevalence: 43% | p<0.001  p<0.001 | 0.61, 0.47-0.79, p<0.001  SES was not included  (Household crowding, footwear outdoors, Anthelmintic usage) |
| #2, India;  Wani SA *et al.*, 2009 | To determine the prevalence of intestinal helminth infection in schoolchildren of Pulwama district, Kashmir, India | Prevalence of intestinal helminthiasis (includes ascariasis, trichuriasis, *E.vermicularis,T.saginata*);  Stool samples were examined using direct smear and zinc sulphate concentra-tion method | Cross-sectional design;  Chi-square test;  N=199 | 2007;  Pulwama district, Kashmir, India;  Urban and rural Schoolchildren;  5-14 yrs;  Eight middle  level schools located in 8 localities of  Pulwama district: asymptomatic children not on antiparasitic treatment were included | Maternal education | Illiterate  Secondary school | 83.9% (94/112)  59.8% (52/87)  Overall prevalence: 73.4% (ascariasis 69.8%, trichuriasis 31.7%) | p=0.01 | NR |
| #2, India;  Wani SA *et al.*, 2007 | To determine prevalence of intestinal parasitic infections in schoolchildren and to assess epidemiological factors associated with the disease | Prevalence intestinal helminthiasis (includes ascariasis, trichuriasis,  *G.lamblia, T.saginata*);  Stool samples were examined using simple smear and zinc sulphate concentra-tion method, then microscopically examined for intestinal parasites | Cross-sectional design;  Chi-square;  N=514 | 2006;  Srinagar City, Kashmir, India;  Urban schoolchildren;  5-14 yrs;  4 middle schools in 4 localities of Srinagar City: children with no disabilities and not on antiparasitic treatment were included | Maternal education | Illiterate  Below 10^th^ class  Above 10^th^ class | (total N)  58.9% (141)  45.7% (291)  29.3% (82)  Overall prevalence: 46.7% (ascariasis 28.4%, trichuriasis 4.9%) | p=0.03 | NR |
| #5, Vietnam;  Mihrshahi S *et al.*, 2009 | To report the impact of an integrated public health program for anaemia prevention and deworming on the prevalence of STH infections in women of reproductive age and identify major risk factors for high STH burden among women | Prevalence any STHs | Cross-sectional design (intervention baseline);  Logistic regression using village as cluster variable;  N=366 | 2005;  Tran Yen and Yen Binh districts, Yen Bai province, north-west Vietnam;  Women of reproductive age;  16-45 yrs;  Stratified multi-stage cluster sampling design was used | Education  SES^[[11]](#endnote-11)^ | No education  Some education  Low  High | Overall prevalence: 83.1% | 7.5 (3.4-16.4), p<0.001  1 (ref)  SES: NR | NR |
|  |  | Prevalence ascariasis |  |  | Education  SES | No education  Some education  Low  High | Overall prevalence 19.2% | 9.0 (3.6-22.7), p=0.001  1 (ref)  SES: NR | 4.6 (1.5-14.2), p=0.01  1 (ref)  (No previous deworming, eating meat <= twice/week, no shoes worn) |
|  |  | Prevalence trichuriasis;  Stool samples were examined using Kato-Katz method |  |  | Education  SES | No education  Some education  Low  High | Overall prevalence 29.1% | 3.7 (1.5-9.1), p=0.03  1 (ref)  3.0 (1.1-8.9), p=0.03  1 (ref) | 6.5 (2.1-19.6), p=0.001  1 (ref)  5.7 (1.6-20.2), p=0.01  1 (ref)  (No other variables) |
| #5, Vietnam;  Pham-Duc P *et al.*, 2013 | To determine prevalence of STH infections and the contribution of exposure to wastewater and human and animal excreta in agriculture | Prevalence any STH | Cross-sectional design;  Logistic regression and generalised  estimating equations (GEE) method;  N=1,425 | 2008-2009;  Nhat Tan and Hoang Tay communes, Kim Bang district, Hanam province, northern Vietnam;  Household members;  >1 yrs;  Households were randomly selected from the list of the Communal People’s Committee | Education  Household SES^[[12]](#endnote-12)^ | No/Primary  Secondary  High school  Poor  Average  Good | (353/723)  (255/520)  (60/182)  (241/478)  (222/473)  (205/474) | 1.9  2.0  1 (ref)  1 (ref)  0.9  0.7 | 1.7 (1.2-2.4)  1.7 (1.2-2.3)  1 (ref)  1 (ref)  1.0 (0.7-1.3)  0.8 (0.6-1.1)  (Occupation, sanitary condition, type of latrine, water source used in household, agricultural practices related with excreta, agricultural practices related with Nhue River water, personal hygiene practices related with agricultural work) |
|  |  | Prevalence ascariasis |  |  | Education  Household SES | No/Primary  Secondary  High school  Poor  Average  Good | (172/723)  (135/520)  (33/182)  (130/478)  (113/473)  (97/474) | 1.4  1.6  1 (ref)  1 (ref)  0.8  0.7 | 1.2 (0.8-1.8)  1.4 (0.9-2.1)  1 (ref)  1 (ref)  1.0 (0.7-1.3)  0.8 (0.5-1.1)  (Same as above) |
|  |  | Prevalence trichuriasis*;*  Stool samples were examined using FECT and Kato-Katz method |  |  | Education  Household SES | No/Primary  Secondary  High school  Poor  Average  Good | (307/723)  (214/520)  (52/182)  (204/478)  (188/473)  (181/474) | 1.8  1.7  1 (ref)  1 (ref)  0.9  0.8 | 1.6 (1.1-2.3)  1.5 (1.1-2.2)  1 (ref)  1 (ref)  1.0 (0.7-1.3)  0.9 (0.7-1.2)  (Same as above) |
| #5, Vietnam;  Trang DT *et al.*, 2007 | To assess prevalence of helminth infections and associated risk factors in a community using wastewater and human excreta in agriculture and aquaculture | Prevalence STHs | Cross-sectional design;  Poisson regression and generalised  estimating equations (GEE) method was used to adjust for intracorrelation within a household;  N=807 | 2002;  Yen So commune, south of central Hanoi, Vietnam;  preschool children and adults from agriculture households;  <6 yrs and >=15 yrs;  400 agriculture households were randomly selected | SES^[[13]](#endnote-13)^ | Poor  Good | (N inf)  40.5% (167)  37.5% (148) | 1.07 (0.86-1.33)  1 (ref) | SES was not included  (Age, gender, duration of excreta compost, use of fresh human excreta in agriculture) |
|  |  | Prevalence ascariasis |  |  | SES | Poor  Good | 23.1% (95)  20.0% (79) | 1.15 (0.85-2.56)  1 (ref) | SES was not included  (Age, urine stored in vessel for plant irrigation) |
|  |  | Prevalence trichuriasis |  |  | SES | Poor  Good | 10.4% (43)  9.1% (36) | 1.12 (0.72-1.76)  1 (ref) | SES was not included  (Age, duration of excreta compost, year-round contact with wastewater) |
|  |  | Prevalence hookworm infection;  Stool samples were examined using direct smear method |  |  | SES | Poor  Good | 22.1% (91)  21.5% (85) | 0.99 (0.74-1.35)  1 (ref) | SES was not included  (Age, gender, use of fresh human excreta in agriculture) |
| #5, Vietnam;  Trang DT *et al.*, 2006 | To assess the relationship between helminth infection and wastewater-fed rice cultivation | Prevalence ascariasis and trichuriasis | Cross-sectional design;  Logistic regression;  N=1,088 | 2003;  My Loc district, Nam Dinh province, northern Vietnam;  Members of agricultural households;  >=15 yrs;  202 agricultural households were randomly selected from the official list of each commune | Education  SES^[[14]](#endnote-14)^ | Low (illiterate to secondary school)  High (high school or university levels)  Low  High | (total N)  (177)  (911)  (548)  (540) | 1.49 (1.08-2.06), p=0.02  1 (ref)  1.29 (1.02-1.64), p=0.03  1 (ref) | 1.35 (0.96-1.91), p=0.08  1 (ref)  1.05 (0.81-1.36), p=0.70  1 (ref)  (Wastewater use, hygienic status latrine, night soil use, use of fresh excreta, treatment of latrine waste, hand washing, use of protective measures, availability of drinking water) |
|  |  | Prevalence ascariasis |  |  | Education  SES | Low (illiterate to secondary school)  High (high school or university levels)  Low  High |  | 1.48 (1.04-2.10)  1 (ref)  1.31 (1.02-1.69)  1 (ref) | NR |
|  |  | Prevalence trichuriasis |  |  | Education  SES | Low (illiterate to secondary school)  High (high school or university levels)  Low  High |  | 1.06 (0.70-1.64)  1 (ref)  1.51 (1.10-2.06)  1 (ref) | NR |
|  |  | Prevalence hookworm infection;  Stool samples were examined using direct smear method |  |  | Education  SES | Low (illiterate to secondary school)  High (high school or university levels)  Low  High |  | 1.21 (0.69-2.26)  1 (ref)  1.02 (0.68-1.54)  1 (ref) | NR |
| #5, Vietnam;  Uga S *et al.*, 2005 | To identify helminth and protozoa infections among schoolchildren in a suburban area and to analyze the relationship between parasitic infection and related factors | High positive for ascariasis (>2,000 eggs/gram) or trichuriasis (>600 eggs/gram);  Stool samples were examined using formalin-ether  sedimentation technique | Cross-sectional design;  Student’s t-test;  N=217 | 2003-2004;  Secondary school of about 1,600 students in hamlet in suburbs of Hanoi, Vietnam;  Schoolchildren;  14-15 yrs;  Randomization: NR | Paternal education  Maternal education  Paternal income (US$)  Maternal income (US$) | 1=Primary school  2=Secondary school  3=High school  4=University  5=Graduate school  1=Primary school  2=Secondary school  3=High school  4=University  5=Graduate schoo3l | Overall prevalence: ascariasis 34%, trichuriasis 67% | Mean SEP among high positive (n=34) vs. negative (n=42) children  2.2±0.54 vs. 2.3±0.69, p>0.05  2.2±0.83 vs. 2.2±0.65, p>0.05  (Education was treated as a continuous variable)  33 US$±42 vs. 44 US$±66, p>0.05;  23 US$±16 vs. 29 US$±20, p>0.05 | NR |
| #6, Malaysia;  Hesham Al-Mekhlafi M *et al.*, 2008 | To investigate the pattern and predictors of STH reinfection among *Orang Asli* school-children | Prevalence STHs **reinfection**;  Stool samples were examined using modified cellophane thick smear method and Harada Mori faecal cultivation method (only for hookworm) | Cohort design;  Logistic regression;  N=120 | 2006;  National Primary School of Betau, Pahang, Malaysia;  *Orang Asli* schoolchildren;  7-12 yrs;  Study was based on repeated screening of this cohort of primary schoolchildren, who attended the school during the visits | Paternal education  (yrs)  Maternal education (yrs)  Household monthly income (US$)^[[15]](#endnote-15)^ | None  ≥6 yrs  None  ≥6 yrs  <141 US$  ≥141 US$ | Prevalence at 3 and 6 mo (N inf)  50.0%/80.9% (35/55)  48.8%/77.5%  (20/31)  49.4%/77.0% (44/67)  50.0%/90.5%  (11/19)  45.8%/78.8% (38/63)  60.7%/82.1%  (17/23) | p>0.05  p>0.05  p>0.05 | Parental education and family income were not included  (Reinfection at 3 months: gender, toilet in house; at 6 months: gender, toilet in house, maternal employment status) |
| #6, Malaysia;  Hesham Al-Mekhlafi M *et al.*, 2007 | To determine STH prevalence and to investigate possible risk factors affecting the pattern of this prevalence among *Orang Asli* children | Prevalence ascariasis;  Stool samples were examined using Kato-Katz method | Cross-sectional design;  Logistic regression;  N=292 | 2006;  Pos Betau, Kuala Lipis, Pahang, Malaysia;  *Orang Asli* schoolchildren;  7-12 yrs;  Schoolchildren from 18 villages around the school that were present during the visits | Paternal education  Maternal education  Household monthly income (US$)^[[16]](#endnote-16)^ | None  ≥6 yrs  None  ≥6 yrs  <141 US$  >141 US$ | (total N)  (127)  (67)  (148)  (46)  (164)  (30) | 1 (ref)  0.96 (0.84-1.22), p=0.90  1 (ref)  0.77 (0.93-1.21), p=0.42  2.0 (1.1-3.7), p=0.03  1 (ref) | Only family income was included: results NR  (Toilet in house, maternal employment status) |
| #6, Malaysia;  Ngui R *et al.*, 2011 | To determine the current epidemiological status and to identify risk factors associated with intestinal parasites infections among communities residing in rural and remote areas | Prevalence intestinal parasitic infections (includes STHs and protozoa infections);  Stool samples were examined using FECT and Kato-Katz method | Cross-sectional design;  Logistic regression;  N=716 | 2007-2009;  8 villages from 5 different states in rural and remote areas, West Malaysia;  Village members;  All ages;  Villages were selected based on village entry approval by the Ministry of Rural and Regional Development and willingness to participate by the village head and community members | Household monthly income (US$)^[[17]](#endnote-17)^ | <150 US$  >150 US$ | 83.5% (456/546)  40.0% (27/68) | 7.60 (5.30-11.13), p<0.001  1 (ref) | 4.93 (3.15-7.73), p<0.001  1 (ref)  (Age, water supply, defecation place) |
| #8, Nigeria;  Ekpo UF *et al.*, 2008 | To evaluate demographic features and intestinal helminth infections among schoolchildren, to investigate hygiene conditions in schools and to identify factors that are essential in the development of sustainable school health programmes | Prevalence any STHs | Cross-sectional design;  Chi-square;  N=232 | 2004-2005;  Ikenne Local Government Area (LGA), one of the twenty LGAs in Ogun State, Nigeria;  Schoolchildren;  4-15 yrs;  3 primary schools were randomly selected after stratifying by public/private and rural/urban: no private schools were located in rural area. In each school, all pupils were enrolled | School type | Govt rural  Govt. urban  Private urban | 63.5% (47/74)  54.9% (50/91)  28.4% (19/67) | p<0.001 | NR |
|  |  | Prevalence ascariasis; |  |  | School type | Govt rural  Govt. urban  Private urban | 39.2% (29/74)  37.4% (34/91)  16.4% (11/67) | p=0.01 | NR |
|  |  | Prevalence trichuriasis |  |  | School type | Govt rural  Govt. urban  Private urban | 24.3% (18/74)  16.5% (15/91)  7.5% (5/67) | p=0.03 | NR |
|  |  | Prevalence hookworm infection;  Stool samples were examined using Kato-Katz method |  |  | School type | Govt. urban  Govt rural  Private urban | 5.5% (5/19)  4.1% (3/74)  0% (0/67) | p=0.16 |  |
| #8, Nigeria;  Kirwan P *et al.*, 2009 | To determine prevalence and intensity of STHs in children and to identify associated risk factors for ascariasis | Prevalence ascariasis;  Stool samples were examined using modified FECT and indirect measure of helminth intensity | Cross-sectional design;  Logistic regression;  N=655 | 2005;  3 semi-urban villages: Moro, Edun-abon, Ipetumodu, Osun state, Nigeria;  Young children;  0-25 months;  Sampling details provided underneath the table.^[[18]](#endnote-18)^ | Paternal occupation^[[19]](#endnote-19)^ (only for children aged ≥6 months) | Farmer  Businessman  Professional | (total N)  27% (89)  11.4% (114)  13.3% (60) | p=0.01 | log-odds estimate  (ref)  -0.95, p=0.02  -0.82, p=0.09,  in text: OR 0.4 for both  (Age, dog ownership) |
| #8, Nigeria;  Ugbomoiko US *et al.*, 2009 | To investigate the effect of water supply, sanitation and parent’s characteristics on prevalence and intensity of ascariasis in children <16 yrs in a rural community | Prevalence ascariasis;  Stool samples were examined using Kato-Katz method | Cross-sectional design;  Logistic regression;  N=440 | 2005-2006;  Ilobu (rural village), Irepodu Local Govt.area of Osun state, Nigeria;  Children;  <16 yrs;  Households were randomly selected from a preliminary household census.^[[20]](#endnote-20)^ | Parental education (≥primary school) | Neither parents  Father only  Mother only  Both parents | 95.8% (182/190)  52.8% (57/108)  31.4% (16/51)  9.9% (9/91) | p<0.001 | 1.93, p=0.01  (unclear how education was included in the model)  (Source of water supply, type of latrine, distance of household to waste dumpsite) |
| #9, Brazil;  Brooker S *et al.*, 2007 | To investigate age-specific relationships between hookworm infection and iron status. | Prevalence hookworm infection;  Stool samples were examined using FECT and Kato-Katz method | Cross-sectional design;  Binomial regression adjusted for household clustering using sandwich estimation;  N=1,247 | 2004;  Americaninhas, Minas Gerais state, south-eastern Brazil;  Household members;  All ages;  Included were residents who stayed in the study area over the previous 24 months, and willing and able to give informed consent and participate.^[[21]](#endnote-21)^ | Household asset index^[[22]](#endnote-22)^ | Poorest  Very poor  Poor  Less poor  Least poor | 79%  83%  74%  69%  46%  (estimated from Figure 1a in the original paper) | p<0.001 | NR |
| #9, Brazil;  Carvalho-Casto FA et al., 2007 | To identify the relationship between intestinal parasitism and nutritional status of children with Indian ascendancy | Prevalence ascariasis | Cross-sectional design;  Chi-square test;  N=307 | 2005;  Santa Isabel do Rio Negro, urban area in northwest Amazonas state, Brazil;  Children;  0.5-7 yrs;  Children included in this survey are attended by the non-governmental organization “Pastoral da Criança” which performs monthly weight measurements of children aged six to 84 months | Family income (US$, N=242) | No wage  133-266 US$  >266 US$ | 61.1% (22/36)  41.4% (67/163)  18.6% (8/43) | p=0.001 | NR |
|  |  | Prevalence trichuriasis |  |  | Family income (US$, N=242) | No wage  133-266 US$  >266 US$ | 36.1% (13/36)  22.1% (36/163)  23.3% (10/43) | p=0.20 | NR |
|  |  | Prevalence hookworm infection;  Stool samples were examined using Baermann-Moraes, Ritchie, Safranin-methylene blue and Kato-Katz method |  |  | Family income (US$, N=242) | No wage  133-266 US$  >266 US$ | 11.1% (4/36)  3.1% (5/163)  0% (0/43) | p=0.02 | NR |
| #9, Brazil;  Cundill B *et al.*, 2011 | To quantify the rate and intensity of re-infection with hookworm and *S. mansoni* 12 months following successful treatment, and investigate the influence of socio-economic, geographical and environmental factors | Hookworm  **Reinfection** rate;  Stool samples were examined using Kato-Katz method | Cohort design;  Interval censored regression;  N=615 | 2004-2006;  Americaninhas, Minas Gerais state, southeastern  Brazil;  Household members;  >=5 yrs;  Inclusion criteria: not received antihelmintic treatment outside the study or participated in another helminth study within the last 30 days; worked or attended school inside the study area; were not severely anaemic (haemoglobin (Hb) concentration <80 g/L); not pregnant | Parental education  Electricity  Ownership land  Wealth index^[[23]](#endnote-23)^ | Not educated  Educated  No  Yes  No  Yes  Poorest  Less poor  Least poor | (N inf)  37% (104)  23% (72)  41% (85)  24% (96)  28% (98)  32% (79)  42% (70)  31% (62)  19% (45) | RR, 95% CI  1 (ref)  0.59 (0.35-0.99), p=0.05  1 (ref)  0.41 (0.24-0.70), p=0.001  1 (ref)  1.15 (0.67-1.97), p=0.62  1 (ref)  0.58 (0.31-1.09)  0.27 (0.14-0.51)  p<0.001 | None of these variables were included  (Gender, house structure, hookworm infection at baseline, hookworm intensity at baseline per 100 epg increase) |
| #9, Brazil;  de Carvalho TB *et al.*, 2006 | To determine prevalences of *Cryptosporidium* sp., *G.duodenalis*, *E. vermicularis* and other entero-parasites in nursery school children, to investigate possible associations between their occurrence and socio-economic and sanitary conditions, | Prevalence intestinal parasites (includes ascariasis, trichuriasis,  *S.stercoralis*,  *E.vermicularis, H.nana,*  *G.duodenalis, Blastocystis hominis,*  *E.coli*, *Cryptosporidium* sp., *E.nana*);  Stool samples were by examined using spontaneous sedimenta-tion in water and fluctuation centrifuge in zinc sulfate | Cross-sectional design;  Chi-square test;  N=279 | 2002;  Botucatu district, São Paulo state, Brazil;  Nursery school children;  0-6 yrs;  From the 17 municipal day care centers located in urban area and rural area, 4 were randomly selected: 3 from urban area and 1 from rural area | Family income (as number of minimum wage equivalents), N=266)  Maternal education (N=265) | <1  1-2  >3  No formal education  First degree  Second degree  University | 71.4% (5/7)  64.3% (72/112)  46.9% (69/147)  80.0% (4/5)  60.3% (88/146)  48.4% (46/95)  26.3% (5/19)  Overall prevalence any intestinal parasite: 53.4% (ascariasis 6.1%, trichuriasis 5.7%) | p=0.01  p=0.01 | NR |
| #9, Brazil;  Fonseca EOL *et al.*, 2010 | To estimate the prevalence and identify risk factors for STH infections among children in municipalities with low human development indices | Prevalence any STH | Cross-sectional design;  Logistic regression adjusting for clustering;  N=2,523 | 2005;  6 municipalities from the Northeast Region and 4 from the North Region, Brazil  Children;  5-14 yrs;  The availability of socio-environmental information  needed  for the study was the criterion for  the selection of the 10 municipalities.  Within the municipalities, families were chosen randomly, and if the family had more than 1 child, the investigated one was also chosen randomly | Maternal education (yrs)  Family income (minimum wage of 123 US$^[[24]](#endnote-24)^) | ≤3 yrs  >3 yrs  ≤1  >1 | (total N)  46.9% (973)  30.1% (1,550)  41.4% (1,821)  22.3% (611)  Overall prevalence: 36.5% | 1.56 (1.41-1.72), p<0.01  1 (ref)  1.86 (1.59-2.18), p<0.01  1 (ref) | 1.69 (1.39-2.06)  1 (ref)  1.75 (1.38-2.23)  1 (ref)  (Garbage near household, >5 household members |
|  |  | Prevalence ascariasis |  |  | Maternal education (yrs)  Family income (minimum wage) | ≤3 yrs  >3 yrs  ≤1  >1 | 32.3% (973)  20.6% (1,550)  29.0% (1,821)  13.7% (611)  Overall prevalence: 25.1% | 1.57 (1.37-1.79), p<0.01  1 (ref)  2.11 (1.71-2.60), p<0.01  1 (ref) | 1.43 (1.16-1.76)  1 (ref)  1.78 (1.35-2.36)  1 (ref)  (Garbage near household, >5 household members, number of rooms in household ≤4) |
|  |  | Prevalence trichuriasis |  |  | Maternal education (yrs)  Family income (minimum wage) | ≤3 yrs  >3 yrs  ≤1  >1 | 15.4% (973)  10.1% (1,550)  13.6% (1,821)  7.7% (611)  Overall prevalence: 12.2% | 1.52 (1.23-1.87), p<0.01  1 (ref)  1.76 (1.31-2.37), p<0.01  1 (ref) | 1.38 (1.03-1.84)  1 (ref)  (Area of residence (rural/urban), presence of running water, >5 household members, number of rooms in household ≤4) |
|  |  | Prevalence hookworm infection;  Stool samples examined using Kato-Katz and sedimentation |  |  | Maternal education (yrs)  Family income (minimum wage) | ≤3 yrs  >3 yrs  ≤1  >1 | 21.9% (973)  11.1% (1,550)  17.6% (1,821)  7.0% (611)  Overall prevalence: 15.3% | 1.97 (1.64-2.37), p<0.01  1 (ref)  2.50 (1.84-3.39), p<0.01  1 (ref) | 1.52 (1.18-1.96)  1 (ref)  1.74 (1.21-2.51)  1 (ref)  (Age, gender, area of residence (rural/urban), garbage near household, >5 household members) |
| #9, Brazil;  Lander RL et al., 2012 | To assess prevalence of poor growth and gastro-intestinal parasites in pre-school children and to explore risk factors | Prevalence STHs;  Stool samples examined using FECT | Cross-sectional design;  Logistic regression using sandwich estimator to account for the sampling procedure;  N=325 | 2010;  Salvador, capital of Bahia state, Brazil;  Preschool children;  3-6 yrs;  7 preschool day care centers located in the city center and peri-urban areas were selected. Included: apparently healthy children enrolled in the 2010 school year | SES (N=58)^[[25]](#endnote-25)^ | Extremely low  Low | Overall prevalence: 17.8% (ascariasis 10.5%, trichuriasis 12.0%, hookworm infection 0.9%) | NR | 2.04 (1.33-3.13), p=0.01  1 (ref)  (Gender, age, deworming treatment, vit. A supplementation |
| #9, Brazil;  Maia MMM *et al.*, 2009 | To determine prevalence of intestinal parasitic infections among children and to investigate risk  factors associated with such infections | Prevalence  parasitic intestinal infection  *(*includes ascariasis, trichuriasis, hookworm infection, *E. vermicularis, S. stercoralis* and protozoa*);*  Stool samples were examined using direct smears and after sedi-mentation using the Hoffman method | Cross-sectional design;  Logistic regression, adjusting for clustering;  N=451 | 2001-2002;  Outpatient clinics in Manaus,  Amazonas state, Brazil;  Children;  0-10 yrs;  Participants were recruited from 4 outpatient clinics and were considered representative of  children attending outpatient clinics in the public health system of Manaus. | Parental education (years)  Monthly household income (minimum wages of roughly 77 US$^[[26]](#endnote-26)^) | ≤8 yrs  >8 yrs  ≤3  3-5  ≥5 | (97/251)  (57/120)  (69/169)  (35/69)  (20/52)  Overall prevalence parasitic intestinal infection: 58.7% (ascariaisis 13.5%, trichuriasis 4.9%, hookworm infection 1.1%) | 1.48 (0.96-2.30), p=0.08  1 (ref)  1.10 (0.58-2.09), p=0.76  0.67 (0.38-1.18), p=0.16 1 (ref) | 1.68 (1.06-2.64), p=0.02  1 (ref)  (Age of child) |
| #9, Brazil;  Pullan RL *et al.*, 2008 | To explore spatial patterns of co-infection with hookworm infection and *S.mansoni* and the role of individual, household and environmental risk factors | Prevalence of hookworm (*N.americanus)*;  Stool samples were examined using FECT and Kato-Katz thick smear | Cross-sectional design;  Logistic regression; (multivariate model also adjusted for clustering);  N=1,332 | 2004;  Americhaninhas, a region in the municipality of Nova Oriente in northeast of Minas Gerias state, southeast Brazil;  Household members;  All ages;  NR | Household asset index^[[27]](#endnote-27)^ | Poorest  More poor  Median  Less poor  Least poor | (total N)  (424)  (143)  (258)  (282)  (225)  Overall prevalence: 71.1% | Coefficient  (ref)  0.06, p=0.88  -0.53, p=0.15  -1.34, p<0.001  -2.42, p<0.001 | Poor: 1 (ref)  Least poor: 0.40, 0.23-0.68  (Gender, age, household crowding, watershed and NDVI (Normalized Difference Vegetation Index) |
| #9, Brazil;  Scholte GCR *et al.*, 2013 | To visualize the distribution of 3 STHs using advanced Bayesian geostatistical modelling coupled with geographical information systems and remote sensing | Prevalence ascariasis | Ecological design;  Bayesian geostatistical logistic regression;  N=1,020 municipalities | 2005-2009: prevalence data  2000: HDI data^[[28]](#endnote-28)^;  1,020 municipalities across Brazil;  Citizens of municipalities;  All ages;  Population-based | HDI  % people with electricity at home  % people with sanitation at home | <0.6  0.60-0.64  0.65-0.69  ≥0.70  <80%  80-89.9%  90-94.9%  ≥95%  Continuous variable | Overall prevalence:  10.3% | NR | 1 (ref)  0.52 (0.50-0.55)  0.57 (0.43-0.66)  0.24 (0.23-0.25)  1 (ref)  0.89 (0.64-1.01)  0.69 (0.63-0.74)  0.56 (0.42-0.63)  0.85, 0.72-0.89  (Land surface temperature day, temperature annual range, annual precipitation, precipitation driest month and warmest quarter, altitude, infant mortality rate, human influence index, % own x rent house, index secure tenure house, % house with phone) |
|  |  | Prevalence trichuriasis |  |  | HDI | <0.6  0.60-0.64  0.65-0.69  ≥0.70 | Overall prevalence:  3.7% | NR | 1 (ref)  0.65 (0.53-0.72)  0.46 (0.40-0.51)  0.20 (0.15-0.23)  (Land surface temperature day, temperature annual range, annual precipitation, precipitation warmest quarter, altitude, infant mortality rate, human influence index, % own x rent house, % owning computer, % people overcrowding, % people subsistence) |
|  |  | Prevalence hookworm infection;  Stool samples were examined using Kato-Katz thick smear |  |  | HDI  % people with electricity at home | Continuous variable  <85%  85-94.9%  ≥95% | Overall prevalence: 4.9% |  | 0.30, (0.22-0.32)  1 (ref)  1.20 (0.91-1.28)  1.98 (1.26-2.44)  (Normalized difference vegetation index (NDVI), mean diurnal range, precipitation wettest month, driest month and warmest quarter, % altitude, % urban population, infant mortality rate, human influence index, % people potable water at house, Index secure tenure house, % house with phone, unemployment rate) |
| #9, Brazil;  Valverde JG *et al.*, 2011 | To estimate prevalence of intestinal parasitic infections through 3 distinct techniques, correlating prevalence rates with family income and age groups as well as assessing household clustering of infections | Prevalence ascariasis | Cross-sectional design;  Chi-square;  N=463 | 2008;  Santa Isabel do Rio Negro, Amazonas state, Brazil;  Urban population;  All ages;  Urban domiciles were randomly selected | Monthly household income (US$) | No wage  198-396 US$  >396 US$ | 31.6% (6/19)  25.4% (84/331)  28.4% (29/102)  Overall prevalence: 26.0% | p=0.79 | NR |
|  |  | Prevalence trichuriasis |  |  | Monthly household income (US$) | No wage  198-396 US$  >396 US$ | 10.5% (2/19)  24.2% (80/331)  14.7% (15/102)  Overall prevalence: 22.5% | p=0.25 | NR |
|  |  | Prevalence hookworm infection;  Stool samples were examined using Ritchie, Baermann-Moraes and Graham (only for children <5 yrs) methods |  |  | Monthly household income (US$) | No wage  198-396 US$  >396 US$ | 15.8% (3/19)  10.9% (36/331)  4.9% (5/102)  Overall prevalence: 9.5% | p=0.005 | NR |
| #14, Nepal;  Parajuli RP *et al.,* 2009 | To evaluate the role of behaviors and nutritional status of people with STH infection in 2 indigenous ethnic groups | Prevalence any STH | Cross-sectional design;  Logistic regression;  N=95 | 2005-2006;  Parsauni village in Sakhawaparsauni Village Development Committee (VDC) of Parsa district, Nepal;  Adult household members;  20-60 yrs;  Mushar households were randomly selected. Tharu households were selected from the lowest economic status non-Mushar households (to minimize effects of differences in economic status between Mushar and Tharu households) | Poverty  School attendance | Yes  No  No  Yes | Overall prevalence: 42.1% | 1.38 (0.59-3.25), p=0.45  1 (ref)  1.02 (0.40-2.61), p=0.96  1 (ref) | None of these variables were included  (Age, gender, ethnicity, walking barefoot outdoor, using soap for hand washing, height (cm)) |
|  |  | Prevalence ascariasis |  |  | Poverty  School attendance | Yes  No  No  Yes | Overall prevalence: 26.3% | 1.33 (0.51-3.51), p=0.56  1 (ref)  0.63 (0.23-1.76), p=0.37  1 (ref) | None of these variables were included  (Age, gender, using soap for handwashing, height (cm)) |
|  |  | Prevalence trichuriasis |  |  | Poverty  School attendance | Yes  No  No  Yes | Overall prevalence: 6.3% | 3.09 (0.35-27.58), p=0.31  1 (ref)  1.74 (0.19-15.70), p=0.62  1 (ref) | NR |
|  |  | Prevalence hookworm infection;  Stool samples were examined using direct wetmount  Lugol’s iodine thin-smear method |  |  | Poverty  School attendance | Yes  No  No  Yes | Overall prevalence: 9.5% | 0.71 (0.18-2.82), p=0.62  1 (ref)  2.92 (0.35-24.65), p=0.32  1 (ref) | None of these variables were included  (Walking barefoot outdoor) |
| #15, Pakistan;  Mehraj V *et al.,* 2008 | To estimate the prevalence of intestinal parasites and its covariates among children from 1–5 years of age residing in an urban slum | Prevalence Intestinal parasitic infections  (includes ascariasis,  *G. lamblia*,  *B.hominis*,  *H. nana*, *E.nana*,  *E. coli* and *Iodoamoeba butschlii*);  Stool samples were examined using FECT | Cross-sectional design;  Logistic regression;  N=218 | 2006;  Ghosia colony, a squatter settlement in the central area of Karachi, Pakistan;  Children;  1-5 yrs;  Simple random sampling to select households from a preliminary census type survey. From households with >1 child, 1 child was randomly selected through simple lottery method | Maternal education  Mean monthly family income (US$)^[[29]](#endnote-29)^  Rented house | ≤Primary  >Primary  Infected: 107 US$  Non-infected: 123 US$  Yes  No | (79/137)  (36/81)  (41/60)  (74/158)  Overall prevalence: 52.8% (ascariasis 16.5%) | 1.7 (0.9-2.9), p=0.06  1 (ref)  1.0 (1.0-1.0), p=0.10^[[30]](#endnote-30)^  1 (ref)  1.5 (1.2-1.9), p=0.004  1 (ref) | 2.0 (1.0-3.9)  1 (ref)  (Age of child, positive history of excessive crying of the child) |
| #16, Ethiopia;  Taye B *et al.*, 2013 | To explore the overlap of STH infection and podoconiosis and to quantify their separate and combined effects on prevalent anemia and hemoglobin levels in podoconiosis patients and health controls | Prevalence STHs  (inlcudes also *S. stercoralis*);  Stool samples were examined using FECT | Cross-sectional design;  Logistic regression;  N=434 | 2010;  Wolaita zone, southern Ethiopia;  clinically confirmed podoconiosis and unmatched controls;  ≥18 yrs;  New adult podoconiosis patients were selected from attending outreach clinics using clinic registration books as the sampling frame; controls were unmatched adults living in the same administrative area | Education | Illiterate  Primary school  (grade 1-8)  Secondary completed | (115/221)  (79/171)  (20/42)  Overall prevalence any STH, (patients and controls combined): 43.8% | 1.19 (0.61-2.31)  0.94 (0.48-1.85)  1 (ref) | 1.17 (0.54-2.55)  0.89 (0.42-1.89)  1 (ref)  (Clinical status (podoconiosis yes/no), gender, age, occupation, shoe wearing habit and stage of podoconiosis disease) |
| #18, Colombia;  Alvarado BE *et al.*, 2006 | To estimate prevalence of pathogenic intestinal parasites (PIP) and to determine risk factors such as social factors, sanitary conditions and nutritional practices and to evaluate the effect of PIP on infant nutritional status | Prevalence ascariasis or trichuriasis;  Stool samples were examined using direct  microscopy and confirmed by Ritchie-Frick procedure | Cross-sectional design;  Logistic regression;  N=136 | 2002;  Municipality of Guapi on the Pacific coast, Colombia;  Young children from urban mothers;  7-18 months;  All mothers with children between 7 and 18 months of age and living in an urban community of coastal Columbia were invited: response was 62% | Maternal education (yrs)  Number of assets owned^[[31]](#endnote-31)^  House floor | 0-5 yrs  >5 yrs  0-1  2-3  4-6  Wood/mud  Tile/cement | 19.4%  34.4%^[[32]](#endnote-32)^  35.6%  27.0%  10.7%  32.0%  12.8%  Overall prevalence; 26.2% (this is inclusive *S.stercoralis* (N=2) which were not included in the analysis) | OR, 90% CI  2.37 (1.20-4.67), p=0.05  1 (ref)  4.57 (1.45-14.3), p=0.02  3.17 (1.03-9.70), p=0.09  1 (ref)  3.19 (1.32-7.70), p=0.06  1 (ref) | NR |
| #19, Tanzania;  Knopp S *et al.*, 2010 | To asses prevalence and intensity of helminth infections and to determine anemia levels in the context of helminth control programs | Prevalence trichuriasis | Cross-sectional design;  Logistic regression stratified by community and adjusting for clustering within households using sandwich estimator;  N=375 | 2008;  Rural Bandamaji and peri-urban Dole, Zanzibar, Tanzania;  Community members;  >=5 yrs;  People who attended the information meeting were invited to participate | Household asset index^[[33]](#endnote-33)^ | Most poor  Least poor  (Asset index was only included for Bandamaji, N=236) | (total N)  (56)  (23)  Overall prevalence: 48.7% | NR | 1 (ref)  0.28 (0.10-0.82), p=0.02  (Age) |
|  |  | Prevalence hookworm infection;  Stool samples were examined using Kato-Katz, Baermann and the Koga agar plate method |  |  | Household Asset Index | Most poor  Very poor  Least poor  (Asset index was only included for Dole, N=139) | (19)  (13)  (51)  Overall prevalence: 32.2% | NR | 1 (ref)  0.11 (0.02-0.58), p=0.01  0.12 (0.04-0.42), p=0.001  (Recent travel history, eating unpeeled fruits, gender, washing hands with soap after defecation) |
| #20, Thailand;  Liabsuetrakul T *et al.*, 2009 | To assess STH in pregnant women, to explore factors associated with STH and evaluate effects of its treatment, both in cure rates and egg reduction rates | Prevalence STHs;  Stool samples were examined using modified Kato-Katz method | Cohort design;  Logistic regression;  N=1,063 | 2006;  Songkhla, Pattani, Yala, Narathiwat provinces, south Thailand;  Pregnant women;  13-46 yrs;  All pregnant women who attended their first prenatal care visit at one of the participating hospitals were invited to participate.  Excluded: gestational age of >32 weeks or had a history of anthelminthic drug allergy | Education  Family monthly income (US$) | <Secondary school  Secondary/diploma  ≥Bachelor  ≤300 US$  >300 US$ | (91/385)  (85/519)  (14/159)  (168/789)  (21/273)  Overall prevalence: 17.9% (ascariasis 10.3%, trichuriasis 5.7%, hookworm infection 6.3%) | p<0.001  3.2 (2.0-5.3), p<0.001  1 (ref) | 2.5 (1.5-4.0)  1 (ref)  (Muslim religion, bathing outside house, no knowledge regarding impact on mother and child) |

NR: not reported; NA: not applicable; inf.: infected; STH: Soil-transmitted helminthes (ascariasis (*A.lumbricoides*), trichuriasis (*T.trichuria*) and hookworm infection (*A.duodenale* and *N.Americanus*)); SES: social-economic status; FECT: formalin-ether concentration technique; HDI: Human Development Index.

**Combined STH and schistosomiasis infection.**

| **Top 20 GBD 2010;**  **Author, Year** | **Aim of study** | **Outcome,**  **detection method** | **Study design, statistical method, sample size** | **Study sample (period, area, population, age, randomization)** | **Measure of SEP** | **Strata** | **Prevalence**  %  (N inf/total N) | **Univariate association**  OR (95% CI) | **Multivariate association**  OR (95% CI)  **(Adjusted for…)** |
| --- | --- | --- | --- | --- | --- | --- | --- | --- | --- |
| Ethiopia;  Gelaw A *et al.*, 2013 | To determine prevalence and risk factors of intestinal parasites among  schoolchildren | Positive intestinal parasite status  (**ascariasis***,* **trichuriasis,**  **hookworm infection***,* ***S.mansoni****, E.histolytica/dispar*, G*.*  *intestinalis, S.stercoralis****,*** *H.nana*);  Stool samples were examined for eggs, larvae, cysts and trophozoites using direct saline smear and formol-ether con-centration methods | Cross-sectional design;  Logistic regression;  N=304 | 2012;  “University of Gondar Community School” in Gondar town, Northwest Ethiopia;  Schoolchildren;  Grade 1 – 8;  Schoolchildren were stratified by grade; the number of students selected was proportional to the number of students in each grade. Systematic random sampling by using class rosters as a sample frame was used for final selection | Family monthly income (US$)^[[34]](#endnote-34)^ | <57.2 US$  57.2-114.4 US$  >114.4 US$ | 38.3% (46/120)  32.8% (39/119)  29.2% (19/65)  Overall prevalence: 34.2% (ascariasis 5.9%, trichuriasis 3.2%, hookworm infection 2.0%, *S.mansoni* 1.3%) | 1.59 (0.26-1.36)^[[35]](#endnote-35)^, p=0.42 | NR |
| Nigeria;  Jombo GT *et al.*, 2010 | To study the association between the level of intestinal parasites and the quality of housing water supply, and sanitary conditions | Prevalence intestinal parasites ( **ascariaisis,** **trichuriasis,** ***S.mansoni***, *S.stercoralis*,*E.histolytica*, *G.lamblia*, *H.nana*, *E.vermicularis)*;  Stool samples were examined for larvae and eggs using formal-ether concentration method | Cross-sectional design;  Mantel-Haenszel X^2^;  N=519 | 2009;  Kuraje village, Zamfara state, northwest Nigeria;  nomadic Fulanis;  6-81 yrs;  Households were selected using systematic random sampling methods | Educational level (≥18 yrs only)  Type of housing | None  Primary  Secondary  Tertiary  Hut  Brick  Cement | 95% (302/318)  11.9% (8/67)  10.2% (5/49)  2.0% (2/10)  86.7% (300/346)  29.4% (46/157)  6.3% (1/16)  Overall prevalence: 66.9% (ascariasis 18.5%, trichuriasis 8.8%, hookworm infection 22.0%, *S.mansoni* 8.4%) | RR  1.4 (1.3-2.2)  Hut and brick vs. cement p<0.001 | NR |

Bold written NTDs were included. Ethiopia is #3 GBD in 2010 for Schistosomiasis and #16 for STHs, Nigeria is #1 for Schistosomiasis and #4 for STHs; NR: Not reported; inf: infected.

1. This paper is also included in the table and text about schistosomiasis. [↑](#endnote-ref-1)
2. Asset-based approach. [↑](#endnote-ref-2)
3. This paper is also included in the table and text about schistosomiasis. [↑](#endnote-ref-3)
4. Based on household assets: radio, TV, telephone, video compact disc, electric fan, electric rice cooker, washing machine, refrigerator, bicycle, motorbike, car, tractor, no. of cows owned, m2 irrigated land for agriculture. [↑](#endnote-ref-4)
5. Purposeful sampling of 2 provinces with high rates of poverty and climate conducive to STH infection. Stratified random sampling: 3 counties per province were randomly selected from the bottom per capita income quartile of counties; then per county 2 towns were randomly selected among towns with higher than mean per capita income and 2 among those with lower than mean per capita income. From each town the central primary school and a randomly selected primary school were chosen. Among the villages that feed into the school 2 were randomly chosen among those with at least 16 students in the school. From each sampled village, 11 students were randomly sampled. In each sampled village, also 11 pre-school children (3-5 years) were randomly sampled from the Registry of Child Immunization. [↑](#endnote-ref-5)
6. Zhang L *et al.* has an overlap with Wang X et al.; the study from Zhang L *et al.* only includes the 3 counties from Guizhou province plus 3 extra counties with minority populations. [↑](#endnote-ref-6)
7. See Wang et al. 2012. All towns were ranked by income per capita and then 2 towns above the mean and 2 towns below the mean were randomly selected. From each town the central primary school and a randomly selected primary school were chosen. From villages with ≥16 students 2 villages were randomly selected and from each village 11 pre-school and 11 schoolchildren were randomly sampled. [↑](#endnote-ref-7)
8. No further details were provided in the paper. [↑](#endnote-ref-8)
9. This appears to be an error in the original paper. When examining the text and other tables in the paper, the percentages should probably be 45.0% for worker and 6.5% for staff. [↑](#endnote-ref-9)
10. SES was not included in the multivariate analysis because SES was strongly related to household crowding, water source, footwear outside, educational level and defecation site (indoor/outdoor). [↑](#endnote-ref-10)
11. Assessed by inclusion in the ‘Free Health Card for the Poor’ scheme. [↑](#endnote-ref-11)
12. Based on indicators of surface of household’s rice field, fish ponds, number of animals, housing characteristics and household assets. [↑](#endnote-ref-12)
13. Compound indicator using a scoring system based on asset ownerships such as motorbike, TV, refrigerator, drilled well, septic tank latrine and house construction. [↑](#endnote-ref-13)
14. Based on 8 items pertaining to type of house construction, ownership of a private well, cattle, motorbike, and telephone. Households with a score below the median were classified as having a low SES and those above median were classified as having a high SES. [↑](#endnote-ref-14)
15. The articles mentions: <450 RM and >450 RM; currency rate used: 1 Malaysian Ringgit = 0.3131 US$, May 1, 2006 (mid of study periods, www.xe.com). [↑](#endnote-ref-15)
16. The articles mentions: <450 RM and >450 RM; currency rate used: 1 Malaysian Ringgit = 0.3131 US$, May 1, 2006 (mid of study periods, www.xe.com). [↑](#endnote-ref-16)
17. The articles mentions: <500 RM and >500 RM; currency rate used: 1 Malaysian Ringgit = 0.2998 US$, January 1, 2008 (mid of study period, www.xe.com). [↑](#endnote-ref-17)
18. Introductory meetings were held in each village and a call was made for children (0-2 yrs) to attend temporary clinics for assessments on arranged dates. Local government health officers helped [↑](#endnote-ref-18)
19. Occupations were also related to exposure of STHs vectors. [↑](#endnote-ref-19)
20. Excluded were: children who refused to participate, were absent during sample visits or had received anthelminthic treatment < 2 weeks before the study. [↑](#endnote-ref-20)
21. Excluded were those who (1) attended school outside of study area; (2) worked fulltime outside of study area; (3) pregnant women; (4) received anthelminthic treatment within the last 24 months as determined by interview. [↑](#endnote-ref-21)
22. Based on housing characteristics and asset ownership: radio, stereo, television, bed, refrigerator, motorcycle, car, presence of electricity and house ownership. [↑](#endnote-ref-22)
23. Based on ownership of farm animals, television, car, motorcycle, fridge, bed, and radio. [↑](#endnote-ref-23)
24. Minimum wage during the study period was R$ 300.00; currency rate used: 1 Brazilian Real=0.4096 US$, June 1 2005 (mid of study period, www.xe.com). [↑](#endnote-ref-24)
25. Based on family and house size and structure, parental education and occupation, marital status, house ownership and household assets, toilet and sewage facilities, type of drinking water, availability of electricity, and susceptibility to flooding during heavy rain. SES scores were divided into two categories: extremely low (≤ 34) and low (≥ 35). [↑](#endnote-ref-25)
26. Household income was compared against the legal minimum monthly wage in Amazones state, which was R$180.00 during the study period. The authors mentions that this was roughly equivalent to 77 US$. [↑](#endnote-ref-26)
27. Information on ownership of household assets was used to construct a wealth index using principal component analysis, using the method of Filmer and Pritchett. [↑](#endnote-ref-27)
28. Provided by the Instituto Brasileiro de Geografia e Estatistica (IBGE). No further details are provided in the original paper, but a web search suggests that the municipal level HDI in Brazil is a composite index consisting of life expectancy at birth, monthly per capita income and indicators for educational attainment (http://www.pnud.org.br/arquivos/Factsheet3englishmetodology.pdf). [↑](#endnote-ref-28)
29. The paper mentions: infected: 6,405 Rs., and non-infected: 7,336 Rs., currency rate used: 1 Pakistani Rupee=0.0167 US$, April 1, 2006 (mid of study period, www.xe.com) [↑](#endnote-ref-29)
30. Income was probably included as continuous variable in in Pakistan Rs. [↑](#endnote-ref-30)
31. Based on ownership of fridge, stove, radio, television and electricity. [↑](#endnote-ref-31)
32. Based on the text of the paper and the OR for maternal education in the paper, the prevalences by educational group appear to be an error in the paper. [↑](#endnote-ref-32)
33. SES was determined according to a wealth index, calculated on the basis of housing characteristics and asset ownership. [↑](#endnote-ref-33)
34. The paper mentions: <1000 EBR, 1000-2000 EBR and >2000 EBR; currency rate used: 1 Ethiopian birr = 0.0572 US$, May 1, 2012 (mid of study periods, www.xe.com). [↑](#endnote-ref-34)
35. Unclear how variable was entered into the model. [↑](#endnote-ref-35)
