# Supplementary material for: Socioeconomic Inequalities in Neglected Tropical Diseases: A Systematic Review
Source: PLoS Negl Trop Dis. 2016 May 12;10(5):e0004546. doi: 10.1371/journal.pntd.0004546 (PMC4865383; doi:10.1371/journal.pntd.0004546)
Supplement: S4 Table — (DOCX) [file pntd.0004546.s006.docx]

**S4 Table. Summary of the literature on socioeconomic inequalities in trachoma, 2004-2013.**

| **Top 20 GBD 2010;**  **Author, Year** | **Aim of study** | **Outcome,**  **detection method** | **Study design, statistical method, sample size** | **Study sample (period, area, population, age, randomization)** | **Measure of SEP** | **Strata** | **Prevalence**  %  (N inf/total N) | | | **Univariate association**  OR (95% CI), p-value | **Multivariate association**  OR (95% CI), p-value  **(Adjusted for…)** |
| --- | --- | --- | --- | --- | --- | --- | --- | --- | --- | --- | --- |
| #4, Ethiopia;  Ayele B *et al.*, 2011 | To assess risk factors associated with ocular chlamydia after repeated  mass antibiotic treatments | Clinically active trachoma (TF/TI);  Examination of upper right tarsal conjunctiva using WHO grading scale | Cross-sectional design;  Logistic regression taking community random effects into account;  N=575 from 364 households | 2009;  12 communities in Goncha Siso Enese woreda, Amhara region, Ethiopia, which received 3 annual **mass azithromycin distributions** (MAD) as part of a trial; 81-92% of children under 10 received antibiotics during MAD;    Children;  0-9 yrs;  Random sample of 50 children from each community | Education of household head (N=563) | No formal education  Some formal education | Overall prevalence: 43.3% | | | 1.40 (0.84-2.34)  1 (ref) | Education not included |
|  |  | RNA evidence of chlamydial infection after MAD;  2 swabs of the upper right tarsal  conjunctiva were analyzed using APTIMA-CT |  |  | Education of household head (N=563) | No formal education  Some formal education | Overall prevalence: 7.2% | | | 1.96 (0.64-6.01)  1 (ref) | Education not included |
|  |  | DNA evidence of chlamydial infection after MAD;  2 swabs of the upper right tarsal  conjunctiva were analyzed using AMPLICOR |  |  | Education of household head (N=563) | No formal education  Some formal education | Overall prevalence: 4.4% | | | 2.48 (0.55-11.14)  1 (ref) | Education not included |
| #4, Ethiopia;  Ketema K *et al.*, 2012 | To assess active trachoma and associated risk factors in children | Prevalence active trachoma (TF/TI);  Eye examination using WHO grading scale: careful inspection of  eye lashes, cornea, limbus, eversion of the upper lid and tarsal conjunctiva using magnifying  binocular lenses and penlight torches | Cross- sectional design;  Logistic regression;  N=792 from 365 households | 2012;  Baso Liben district, East Gojjam, Amhara Regional state, Ethiopia; MDA status not reported;  Children;  1-9 years;  Two-stage random cluster sampling: (i) 5 villages - randomly selected and (ii) households - random walk method. Number of children were taken proportionally to population size^[[1]](#endnote-1)^ | Education household head  Household monthly income (US$)^[[2]](#endnote-2)^ | Illiterate  ≥Primary  <34.5 US$  34.6-69.0 US$  69.1-115.0 US$  >115.0 US$ | | | 35.8% (78/219)  6.6% (10/146)  18.7% (148/443)  5.2% (41/239)  0.3% (2/75)  0% (0/35)  Overall prevalence: 24.1% | 7.85 (4.85-12.70)  1 (ref)  p≤0.001  4.60 (2.81-7.53)  2.37 (1.37-4.09)  1.01 (0.53-1.93)  1 (ref)  p≤0.001 | 5.18 (2.93-9.17)  1 (ref)  p≤0.001  2.98 (1.85-7.85)  3.89 (1.87-8.09)  1.29 (0.56-3.81)  1 (ref)  p≤0.001  (Agro-climate, access to water source, water consumption, frequency of latrine usage, soap usage, clean face of child, number of flies on face of child, knowledge of household head |
| #4, Ethiopia;  Mesfin MM *et al.*, 2006 | To investigate the prevalence of and potential risk factors for trachoma in order to institute effective control strategies | Prevalence active trachoma (TF/TI);  WHO clinical grading using torch light and a magnifying loupe | Cross- sectional design;  Logistic regression: multivariate analyses were adjusted for clustering using Huber-White sandwich estimator  N=3,900 from 1,200 households | 2003;  Tigray, northern Ethiopia; MDA status not reported.  Household members;  >10 yrs;  From 6 districts a total of 48 villages were selected using multistage cluster random sampling | Education | Illiterate  Literate | | TF, TI  13.8%, 28%  (385, 788 / 2,794)  11%, 22,5%  (123, 249 / 1,106)  Overall prevalence:  TF 13%, TI 27% | | (TF/TI)  1.93 (1.65-2.26)    1 (ref) | 1.38 (1.13-1.69)  1 (ref)  (Gender, age, residence (urban/rural), presence of latrine, waste disposal, chimney in kitchen, number of days since respondent washed their face, use of soap for face washing, time of fetching water/day (min) |
|  |  | Prevalence TS |  |  | Education | Illiterate  Literate | 20% (556/2,794)  12% (133/1,106)  Overall prevalence: 17.7% | | | 2.57 (2.1-3.2)  1 (ref) | 0.98 (0.72-1.34)  1 (ref)  (Same variables as above) |
|  |  | Prevalence TT |  |  | Education | Illiterate  Literate | 4.1% (115/2,794)  1.5% (17/1,106)  Overall prevalence: 3.4% | | | 4.2 (2.4-6.9)  1 (ref) | 1.4 (0.67-2.89)  1 (ref)  (Same variables as above) |
| #4, Ethiopia;  Ngondi J *et al.*, 2008 | To investigate potential risk factors associated with trachoma in order to tailor the delivery of the SAFE strategy | Prevalence  active trachoma (only TF/any TI);  Each eye was graded (WHO) separately | Cross-sectional design;  Ordinal logistic regression, adjusted for clustering using GLM;  N=5,427 from 2,845 households | 2006-2007;  Amhara region, north-western  Ethiopia; MDA status not reported;  Children;  1-9 yrs;  Multistage cluster random sampling was used to select 160 clusters to give a sample of 4000 households. All eligible participants in the selected households were examined for trachoma. | Household electricity  Type of roof | No  Yes  Thatch  Tin | Only TF, any TI  25%, 22%  14%, 7%  25%, 24%  25%, 19%  Overall prevalence:  only TF 24.9%, any TI 21.9% | | | severity of active trachoma (no TF & no TI; TF  only; any TI)  4.1 (2.3-7.5), p<0.001  1 (ref)  1.3 (1.1-1.6), p=0.01  1 (ref) | 2.4 (1.3-4.3), p=0.004  1 (ref)  1.3 (1.0-1.5), p=0.02  1 (ref)  (Ocular/nasal discharge, altitude (m)) |
| #4, Ethiopia;  Regassa K *et al.*, 2004 | To determine prevalence of trachoma and its risk factors among adults | Prevalence active trachoma (TF/TI);  Each eye was examined using a binocular magnifying loupe and torchlight | Cross-sectional design;  Logistic regression;  N=855 | 2002;  Damot Gale district, south Ethiopia; MDA status not reported;  Villagers in a trachoma hyperendemic area;  >15 yrs;  Multi-stage sampling method: 11 out of 54 *kebeles* were randomly selected from which villages and, next, households were selected using simple random sampling | Education | Illiterate  Literate | 17.8% (152/535)  2.8% (24/320)  Overall prevalence: 20.6% | | | 4.89 (3.04-7.94), p<0.001  1 (ref) | NR |
| #4, Ethiopia;  Vinke C *et al.*, 2011 | To study the influence of 14 predictor variables on active trachoma risk and disease severity in children | Prevalence active trachoma (TF and TI);  Eye examination using torch lights and binocular loupes | Cross-sectional design;  Mixed effects logistic regression with random effects;  N=1,513 from 306 households | 2007;  Damboya and Kedida Gamela districts in Kembata Tembaro zone, Southern Ethiopia; MDA status not reported;  Children;  1-9 yrs;  30 *gotts* (villages) among 28 *kebeles* (smallest government unit) were selected using systematic random sampling taking population size into account; 34 households were chosen from each village through random walk  sampling | Household expenses/  person (US$)^[[3]](#endnote-3)^ | Continuous variable | Overall prevalence: NR | | | NR | 0.99 (0.98-0.99), p=0.03  For each increase in income with 0.1128 US$, the odds of active trachoma declined with 0.99  (Age, face cleanliness) |
| #6, Brazil;  Da Rocha Luchena *et al.*, 2010 | To evaluate the epidemiological factors of trachoma | Trachoma (TF, TS, TT, ocular C.*trachomatis*;  Physical examination using loupe following WHO guidelines | Cross-sectional design;  Multinominal logistic regression;  N=412 | 2007;  Araripe village, Ceara state, Brazil; MDA status not reported;  Villagers;  All ages;  62% of citizens was examined | Education  Income (minimum wage) | Illiterate  1^st^ grade incomplete  1^st^ grade complete  2^nd^/3^rd^ grade    <1  ≥1 | % in SEP category among people without/with trachoma  20.4%/ 49.1%  14.1%/29.6%  50.0%/17.6%  15.5%/3.7%  45.7%/16.7%  54.3%/83.3%  Overall prevalence of any trachoma: 26.2% | | | P-values for difference in educational and wealth distribution (for each SEP category) between people with/without trachoma  p=0.24  p<0.001  p=0.20  p=0.90  p<0.001  p=0.05 |  |
| #9, Sudan;  Edwards T *et al.*, 2012 | To estimate prevalence for planning trachoma interventions, to identify risk factors, and to investigate the effect of different sampling approaches on study conclusions | Prevalence TF for 1-9 yrs | Cross-sectional design;  Logistic regression adjusted for clustering;  N=2,406 | 2010;  Unity state, north South Sudan;  Household members; MDA status not reported;  1-9 yrs and ≥15 yrs;  One county from 9 was excluded because no SAFE intervention had occurred. Number of villages surveyed was based on the population size of the county. Within each village, 20 households were randomly selected using the sketch map and segmentation method | Education of household head | None  ≥1 yr primary  ≥1 yr secondary | 70.7% (1,509/2,134)  72.1% (168/233)  50.0% (14/28)  Overall prevalence: 71.0% (adjusted)^[[4]](#endnote-4)^  70.5% (unadjusted) | | | 1 (ref)  1.17 (0.80-1.71)  0.54 (0.20-1.44)  p=0.32 | Education was not included |
|  |  | Prevalence TT for ≥15 yrs;  Each eye was examined separately using torch and binocular loupe (WHO grading) | N=1,602 |  | Education of household head | None  ≥1 yr primary  ≥1 yr secondary | 16.1% (224/1,395)  9.8% (16/164)  6.1% (2/33)  Overall prevalence: 13.5% (adjusted)^d^  15.1% (unadjusted) | | | 1 (ref)  0.61 (0.32-1.16)  0.37 (0.07-1.88)  p=0.14 | Education was not included |
| #11, Tanzania;  Harding-Esch T *et al.*, 2010 | To assess prevalence of, and risk factors for active trachoma and ocular C.*trachomatis* infection pre-treatment as part of the Partnership for the Rapid Elimination of Trachoma (PRET) cluster randomized controlled trial in The Gambia and Tanzania | Prevalence active trachoma (TF);  Both upper eyelids were graded using torch/sun and loupe | Cross-sectional design;  Random effects logistic regression;  N=3,198 | 2008;  Kongwa district, Dodoma region, Tanzania; MDA status not reported;  Children;  0-5 yrs;  Communities were selected based on having an active trachoma prevalence above 20% in preliminary surveys | Household head education (yrs) | 0-6 yrs  ≥7 yrs | 33.8% (567/1,678)  27.2% (413/1,518)  Overall prevalence: 30.9% | | | 1 (ref)  0.70 (0.56-0.87), p=0.001 | 1 (ref)  0.77 (0.60-0.98), p=0.03  (Age, gender, ocular/nasal discharge, flies on face, amplicor positive, number of people/household) |
|  |  | Prevalence ocular C.*trachomatis;*  Swab from right eye using Amplicor test |  |  | Household head education (yrs) | 0-6 yrs  ≥7 yrs | 24.8% (405/1,634)  18.7% (278/1,486)  Overall prevalence: 21.9% | | | 1 (ref)  0.55 (0.40-0.76), p<0.001 | 1 (ref)  0.51 (0.35-0.74), p<0.001  (Age, gender, ocular/nasal discharge, time to water) |
| #11, Tanzania;  Jansen E et al., 2007 | To assess to what extent trachoma is a disease of the poor, and trachoma services reach the poor in Tanzania (and Vietnam) | Prevalence active trachoma (defined here as TF)  for 1-9 yrs  Averting eyelids checked to establish the number of follicles present in the upper tarsal conjunctiva | Cross-sectional design;  Concentration indices (CI, degree of inequality);  N=6,855 | 2004;  Tunduru, Sikonge, Magu, Ruangwa,  and Handeni, largely rural districts spread over the country, Tanzania; No MDA;  Children and adults;  1-9 yrs and ≥15 yrs;  Stratified sampling design: random selection of 20 villages within each district and approximately 34 households within each village. The probability of selection of a village was proportional to its population size | Wealth quintile^[[5]](#endnote-5)^ | 1 (poorest)  2  3  4  5 (least poor) | 35.3%  36.0%  29.3%  29.0%  23.1%  Overall prevalence: 30.6% | | | Concentration Index: -0.0942, p<0.05 | NR |
|  |  | Prevalence TT for ≥15 yrs;  At least one eyelash rub-  bing the eyebal | N=9,380 |  | Wealth quintile | 1 (poorest)  2  3  4  5 (least poor) | 4.9%  5.6%  4.5%  4.1%  2.1%  Overall prevalence: 4.2% | | | Concentration Index:  -0.1647, p<0.05 |  |
| #11, Tanzania;  Polack S *et al.*, 2006 | To explore the relationship between active trachoma in children and detailed measures of water including water collection time, quantity of water collected and household water use patterns in a trachoma endemic village | Prevalence active trachoma (TF and/or TI) in either eye;  Both eyes were examined using binocular loupes; grading according to WHO simplified trachoma scale. | Cross-sectional design;  Logistic regression using generalized estimating equation;  N=914 from 416 households | 2003;  Shimbi Mashiriki village, Rombo district, northern Tanzania; No MDA;  Children;  1-9 yrs;  All households with children aged 1-9 years were included | Multidimensional wealth index  (quartiles)^[[6]](#endnote-6)^  Household monthly expenditure per capita (US$) | Poorest quartile  Second quartile  Third quartile  Richest quartile  <2 US$  2-3.69 US$  3.70-6.50 US$  >6.50 US$ | 21.6% (55/255)  19.9% (37/186)  14.1% (32/227)  17.9% (38/212)  13.6% (29/214)  20.0% (41/205)  23.0% (56/243)  16.2% (38/234)  Overall prevalence: 18.4% | | | 1 (ref)  0.89 (0.49-1.67)  0.59 (0.32-1.08)  0.90 (0.51-1.68)    1 (ref)  1.57 (0.81-3.05)  1.94 (1.04-3.62)  1.05 (0.53-2.09)  (Both variables adjusted for age, gender, household clustering) | 1 (ref)  1.01 (0.55-1.87)  0.66 (0.35-1.25)  1.52 (0.79-2.90)  (Age, gender, household clustering, reported water collection time (min), years lived in the village, cattle in same room as people overnight)  1 (ref)  1.73 (0.89-3.38)  2.20 (1.17-4.14)  1.19 (0.58-2.41)  (Age, gender, household clustering, household SES) |

TF: follicular trachoma; TI: intense inflammatory trachoma; TT: trichiasis; TS: Trachomatous scarring; inf: infected; SEP: socioeconomic position; MDA: mass drug administration.

1. Excluded were children who had eye injury and/or who were seriously sick or who were not at home at the time of study or < 1 year old. [↑](#endnote-ref-1)
2. The paper reported: <600 EBR, 601-1200 EBR, 1201-2000 EBR and >2000 EBR; currency rate used: 1 Ethiopian birr = 0.0575 US$, March 1, 2012 (mid of study period, www.xe.com). [↑](#endnote-ref-2)
3. The paper mentions Ethiopian birr; currency rate used: 1 Ethiopian birr = 0.1128 US$, May 1, 2007 ([www.xe.com](http://www.xe.com)). [↑](#endnote-ref-3)
4. Adjusted for age, sex, county, between-village variation and between-household variation using random effects regression models. [↑](#endnote-ref-4)
5. Based on asset ownership: electricity, bicycle, radio, television, refrigerator, motorcycle, car, number of household members/sleeping room, source drinking water, toilet facilities, floor material. [↑](#endnote-ref-5)
6. Multidimensional index including housing, durable goods, education, and occupation. [↑](#endnote-ref-6)
