# Supplementary material for: Socioeconomic Inequalities in Neglected Tropical Diseases: A Systematic Review
Source: PLoS Negl Trop Dis. 2016 May 12;10(5):e0004546. doi: 10.1371/journal.pntd.0004546 (PMC4865383; doi:10.1371/journal.pntd.0004546)
Supplement: S5 Table — (DOCX) [file pntd.0004546.s007.docx]

**S5 Table: Summary of the literature on socioeconomic inequalities in Chagas’ disease, 2004-2013.**

| **Top 20 GBD 2010;**  **Author, Year** | **Aim of study** | **Outcome,**  **detection method** | | **Study design, statistical method, sample size** | | **Study sample (period, area, population, age, randomization)** | | **Measure of SEP** | | **Strata** | **Prevalence**  %  (N inf/total N) | **Univariate association**  OR (95% CI), p-value | | | **Multivariate association**  OR (95% CI)  **(Adjusted for…)** | |  |
| --- | --- | --- | --- | --- | --- | --- | --- | --- | --- | --- | --- | --- | --- | --- | --- | --- | --- |
| #1, Brazil;  Borges-Pereira J *et al.*, 2006 | To study the association between Chagas and age, gender, blood transfusion and spontaneous abortion | anti *T. cruzi* sero-positivity;  Blood samples were examined using indirect immunofluorescence test | | Cross-sectional design;  Chi-square test;  N=36,399 people from 11,439 households | | 2002;  Piaui State, Brazil;  Rural zone of the entire state;  All ages;  Random sample of inhabitants | | Education  (only for >10 yrs) | | Illiterate  Primary  Secondary  Tertiary  Illiterate  Literate (primary-tertiary education) | 4.1% (407/9,850)  1.6% (268/17,095)  1.1% (10/919)  0.9% (1/107)  4.1% (407/9,850)  1.5% (279/18,121)  Overall prevalence: 1.9% | RR (95% CI)  2.68 (2.31-3.12), p<0.001  1 (ref) | | | NR | |  |
| #1, Brazil;  Pinto FS *et al.*, 2013 | To assess the epidemiological characteristics of mothers with Chagas and the birth conditions of neonates born to these mothers | anti *T. cruzi* sero-positivity;  Two serological tests | | Case-control design;  Logistic regression;  N= 401 infected, 404 uninfected mothers (matched by age) | | 2005-2006;  Minas Gerais State, Brazil;  Mothers who recently gave birth;  ≥16 yrs;  Cases: from serological survey of Newborn Screening Program of Minas Gerais, conducted in all 853 municipalities of Minas Gerais State  Controls: randomly selected from the “System of Information on Live Births” database | | | Education (yrs) | <8 yrs  ≥8 yrs | (cases/controls)  (318/233)  (74/167) | 3.08 (2.23-4.25), p<0.01  1 (ref) | | | 2.61 (1.82-3.74)  1 (ref)  (Number of children from previous pregnancies) | |  |
| #3, Argentina;  Llovet I *et al.*, 2011 | To compare social patterning of the Chagas burden by examining socio-demographic predictors of self-reported Chagas and the presence of *vinchucas^[[1]](#endnote-1)^* in 2 rural areas known to have experienced different surveillance and control interventions | | Household member with Chagas disease;  Self-reported | | Cross-sectional design;  Logistic regression;  N=400 households  (N=200 in Av, N=200 in Sp) | | 2006;  Avellaneda (Av) and Silípica (Sp) in Santiago del Estero, northern Argentina;  Rural households;  All ages;  Av and Sp rank among the poorest *departamentos* in the country.  Two-stage sampling (census segments and households within each segment). | | Education household head | < Primary  Primary or more  < Primary  Primary or more | (total N)  Av  (138)  (57)  Sp  (93)  (100)  Total prevalence: 37.4% in Av, 13.7% in Sp | | Av  1.63 (0.82-3.24)  1 (ref)  Sp  2.75 (1.06-7.13)  1 (ref) | Av  1.97 (0.94-4.15)  1 (ref)  Sp  4.03 (1.37-11.82)  1 (ref)  (Age household head, gender household head, single-parent household, occupation household head, household size) | | | |
| #5, Colombia;  Cucunuba ZM *et al.*, 2012 | To determine prevalence of Chagas in pregnant women and its associated risk factors | anti *T. cruzi* sero-positivity;  blood samples were examined using ELISA, positive results were confirmed by at least two of the following three tests:  ELISA, IFAT, and IHA | | Cross-sectional design;  Logistic regression;  N=982 | | 2010;  El Yopal, capital of Casanare, Orinoco River Basin region, eastern Colombia;  Pregnant women,  13-46 yrs;  All pregnant women at the health centers located in El Yopal, where most births and pregnant women from this department receive medical care, were included | | SES^[[2]](#endnote-2)^  Education | | I  II/III  Primary school incomplete  Primary school completed  High school incomplete  High school completed  University | 4.8% (6/282)  2.1% (33/693)  12.5% (13/104)  5.8% (9/156)  2.8% (9/322)  2.3% (6/256)  0.7% (1/138)  Overall prevalence: 4% | | 2.1 (1.0-5.5), p=0.04  1 (ref)  19.6 (2.5-152.2)  8.4 (1.0-67.0)  3.9 (0.6-24.2)  3.3 (0.7-27.4)  1 (ref), p<0.01 | | | SES was not included  10.2 (1.6-82.7)  5.5 (0.7-45.8)  3.3 (0.4-27.9)  2.3 (0.2-20.9)  1 (ref)  (Age, residence urban/rural and knowledge of vector) | |

NR: Not Reported; ELISA: enzymelinked immunosorbent assay; IFAT: immunofluorescent assay; IHA: indirect hemagglutination assay; inf: infected.

1. Vinchuca: triatomine bug, vector of Chagas disease. [↑](#endnote-ref-1)
2. SES based on Sistema de Identificación de Potenciales Beneficiarios de Programas Sociales, Colombia 2010. [↑](#endnote-ref-2)
