# Supplementary material for: Socioeconomic Inequalities in Neglected Tropical Diseases: A Systematic Review
Source: PLoS Negl Trop Dis. 2016 May 12;10(5):e0004546. doi: 10.1371/journal.pntd.0004546 (PMC4865383; doi:10.1371/journal.pntd.0004546)
Supplement: S6 Table — (DOCX) [file pntd.0004546.s008.docx]

**S6 Table: Summary of the literature on socioeconomic inequalities in leprosy, 2004-2013.**

| **Top 20 GBD 2010;**  **Author, Year** | | **Aim of study** | **Outcome,**  **detection method** | **Study design, statistical method, sample size** | **Study sample (period, area, population, age, randomization)** | **Measure of SEP** | **Strata** | **Prevalence**  %  (N inf/total N) | **Univariate association**  OR (95% CI) | **Multivariate association**  OR (95% CI)  **(Adjusted for…)** |
| --- | --- | --- | --- | --- | --- | --- | --- | --- | --- | --- |
| #2, Brazil;  Imbiriba ENB *et* *al.*, 2009 | To analyze the epidemiology of leprosy according to spatial  distribution and living conditions of the population | | Leprosy detection rate (annual mean # of leprosy cases between  1998-2004 per 10,000  population)  National Information System for Notifiable Diseases (SINAN) | Ecological design;  Logistic regression;  N= 9,919,029 citizens, number of cases=4,104 | 1998-2004;  Manaus City, Northern Brazil;  Citizens of Manaus;  All ages;  Residents of 1,536 census tracts | Living conditions at census tract level^[[1]](#endnote-1)^ | Low  Average/low  Average  High | Mean detection rate: 4.21 per 10,000 | 4.43 (3.14-6.24), p<0.001  3.05 (2.15-4.32), p<0.001  1.67 (1.14-2.44), p=0.01  1 (ref) | NR |
| #2, Brazil;  Kerr-Pontes LRS *et al.*, 2006 | | To identify socioeconomic, environmental, and behavioral factors associated  with leprosy in patients with no known leprosy contacts | Clinical diagnosis of leprosy;  Typical skin lesion with  loss of sensitivity and/or enlargement of one of the major nerves  with loss of sensitivity and/or positive skin smear for *M. leprae* | Case-control design;  Logistic regression, cluster effect (municipality) taken into account;  N = 226 cases,  857 controls | 2002;  4 municipalities  in Ceará state (one of poorest states in north-eastern Brazil);  Patients from Primary Health Care Centers;  >18 yrs;  4 out of 19 municipalities with the highest detection rate, reflecting geographical and socio-economic diversity of the State.  Cases: leprosy patients diagnosed in the previous 2 years who returned for routine monitoring  Controls: patients from same clinic and municipality coming for reasons other than skin problems | Schooling^[[2]](#endnote-2)^  Food shortage at any time  Access to safe drinking water 10 yrs previously  Sewage disposal 10 yrs previously  Sand/mud floor 10 yrs previously | Low  Middle  High  Ever  Never  No  Yes  No  Yes  Yes  No | Cases/controls  130/387  56/226  40/244  63/163  161/687  91/298  133/546  41/111  180/741  44/119  182/737 | 2.05 (1.29-3.27)  1.51 (0.93-2.47)  1 (ref)  1.65 (1.11-2.42)  1 (ref)  1.17 (0.96-1.43)  1 (ref)  1.44 (0.95-2.80)  1 (ref)  1.46 (1.04-2.06)  1 (ref) | 1.87 (1.29-2.74)  1.50 (0.91-2.50)  1 (ref)  1.54 (1.45-1.63)  1 (ref)  (Age, gender, weekly regular bath in open water, low frequency of changing bed linen, BCG scar) |
| #2, Brazil;  Murto C *et al.,* 2013 | | To examine (i) migration as a risk factor for leprosy and (ii) social and behavioral risk factors for leprosy among  past 5-year migrants | Clinical diagnosis of leprosy;  National Information System for Notifiable Diseases (SINAN) | Case-control design;  Logistic regression and Fishers exact test;  N=80 cases  55 controls (matched) | 2009-2010;  Maranhão state, Brazil (4 leprosy endemic municipalities);  Cases from SINAN database, controls from Program for Family Health;  ≥15 yrs; past 5 year migrants  Controls were randomly selected by age and sex. | Education:  family illiteracy^b^  Income (US$)^[[3]](#endnote-3)^  Public waste service | Yes  No  ≤297 US$  >297 US$  No  Yes | (cases/controls)  (32/12)  (39/39)  (38/17)  (39/37)  (22/6)  (58/49) | 2.67 (1.13-6.51), p=0.02  1 (ref)  2.12 (0.97-4.71), p=0.049  1 (ref)  3.1 (1.1–10.02), p=0.03  1 (ref) | NR |
| #2, Brazil;  Sales AM *et al.*, 2011 | | To evaluate risk factors associated with developing leprosy among the contacts of newly diagnosed leprosy patients | Chance that contacts of leprosy patients get leprosy;  Examined by specialized dermatologists and  neurologists | Cohort design;  Two-level logistic regression  (1^st^ level: contacts, 2^nd^ level: index cases);  N= 1,201 newly diagnosed patients and  6,158 contacts (319 co-prevalent cases, identified at first examination after diagnosis of index case;  133 incident cases that were apparently leprosy free at time of diagnosis of index but who developed leprosy during follow-up) | 1987-2007;  Leprosy Outpatient Clinic, Rio de Janeiro, Brazil;  Contacts of newly diagnosed leprosy patients;  All ages;  All contacts who returned to the clinic for examination were  eligible | Education level (yrs)  *Of index cases*  *Of contacts*  Income level  (number of minimum wages)  *Of index cases*  *Of contacts* | <4 yrs  4-10 yrs  >10 yrs  <4 yrs  4-10 yrs  >10 yrs  <4 yrs  4-10 yrs  >10 yrs  <4 yrs  4-10 yrs  >10 yrs  <2  2-3  >3  <2  2-3  >3  <2  2-3  >3  <2  2-3  >3 | (232/3,829)  (70/1,497)  (17/850)  (78/3,829)  (31/1,497)  (24/850)  (255/4,327)  (25/718)  (39/1,112)  (98/4,327)  (7/718)  (28/1,112)  (73/1,254)  (84/1,428)  (40/1,491)  (27/1,254)  (33/1,428)  (24/1,491)  (82/1,162)  (150/2,435)  (87/2,561)  (25/1,162)  (69/2,435)  (39/2,561)  Total prevalence among contacts: 7.3 | Co-prevalent  3.31 (1.87–5.58)  2.53 (1.37–4.64)  1 (ref)  Incident  0.70 (0.40-1.21)  0.70 (0.37-1.31)  1 (ref)  Co-prevalent  1.50 (1.03-2.19)  0.93 (0.53-1.65)  1 (ref)  Incident  0.91 (0.56-1.47)  0.38 (0.15-0.94)  1 (ref)  Co-prevalent  2.17 (1.34–3.52)  2.31 (1.44–3.70)  1 (ref)  Incident  1.36 (0.70-2.63)  1.48 (0.78-2.78)  1 (ref)  Co-prevalent cases  2.18 (1.50-3.17)  1.85 (1.35–2.54)  1 (ref)  Incident  1.47 (0.84-2.60)  1.90 (1.21-2.96)  1 (ref) | Co-prevalent  2.72 (1.54-4.79)  2.40 (1.30-4.42)  1 (ref)  Incident  0.60 (0.34-1.06)  0.70 (0.37-1.32)  1 (ref)  Co-prevalent  1.43 (0.96 -2.15)  1.08 (0.61-1.94)  1 (ref)  Incident  0.82 (0.49-1.36)  0.40 (0.16-1.01)  1 (ref)  (Index cases: gender and bacillary index; Contacts: gender, age, blood relationship, type of close association, BCG scar and BCG vaccine) |
| #2, Brazil;  Schmitt JV *et al.*, 2010 | | To compare armadillo meat consumption among leprosy patients with that among controls | Leprosy;  NR | Case-control design;  Logistic regression;  N=121 patients and 242 controls  (matched) | 2005-2009;  Reference dermatological center in Curitiba, state of Paraná (PR), Brazil;  Leprosy patients and controls;  >15 yrs;  All patients >15 yrs with leprosy were eligible, controls were selected among patients with other skin diseases | Family income (minimum wages) | <1  1-5  >5 | (cases/controls)    (41/24)  (74/186)  (6/32) | OR for having a particular income level, cases vs. controls  4.66 (2.65-8.20), p<0.01  0.47 (0.30-0.76), p<0.01  0.34 (0.14-0.84), p=0.02 | OR for leprosy  7.03 (2.27-21.76), p<0.01  1.63 (0.61-4.38), p=0.34  1 (ref)  (Gender, age, hometown population size, current residence, family size, contact with leprosy patients, wild animal food intake and armadillo meat intake) |
| #2, Brazil;  Silva DRX *et al.*, 2010 | | To analyze the association between social and environmental indicators and the Hansen's disease new case detection rate (HNCDR) in the Brazilian Amazon | Leprosy detection rate (number of new cases per 10,000 population);  National Information System for Notifiable Diseases (SINAN) | Ecological design;  Pearson correlation;  N=NR | 2006;  Amazon, Brazil;  Entire population;  All ages;  Population-based | Human development index (HDI) |  | Average and median detection rate for total population:  7.78; 7.38 (Bayesian correction)^[[4]](#endnote-4)^  7.81; 7.54 (not corrected)^[[5]](#endnote-5)^ | Pearson correlation coefficient. The higher the HDI, the lower the detection rate:  r = -0.36, p < 0.001^[[6]](#endnote-6)^ | NR |
| #7, Bangladesh;  Feenstra SG *et al.*, 2011 | | To study the association between different socio-economic factors and the risk of acquiring clinical signs of leprosy | Clinical signs of leprosy;  NR | Case-control design;  Logistic regression;  N = 90 cases and 199 controls | 2009;  Northwest Bangladesh (2 leprosy endemic districts)  Mainly rural area;  > 5 yrs;  Recently (2009) diagnosed leprosy patients and controls from random cluster sample (only including 1 patient per household to avoid clustering). Excluding cases in which the economic situation had changed due to the disease. | Wealth quintiles^[[7]](#endnote-7)^  Income level (Bangladeshi Taka)  Highest education in household  (yrs)  Food shortage last year | 1 (poorest)  2  3  4  5 (least poor)  Continuous variable  ≤5 yrs  >5 yrs  Yes  No | (cases/controls) (25/40)  (20/40)  (16/40)  (17/40)  (12/39)    (41/86)  (49/113)  (47/128)  (43/71) | 1 (ref)  0.80 (0.38-1.67)  0.64 (0.30-1.38)  0.68 (0.32-1.45)  0.49 (0.22-1.12)  Linear trend:  OR = 0.85 (0.71–1.02)  p-value trend: 0.08  1.00, 1.00-1.00, p=0.15  1.10 (0.67-1.81) ,p=0.71  1 (ref)  1.65 (1.00-2.74), p=0.05  1 (ref) | 1.79 (1.06-3.02), p=0.03  1 (ref)  (Age, gender) |

NR: Not Reported; inf: infected.

1. Living condition based on: number of people/household, household head with no schooling or <1 year of education and household head with no income (source: Brazilian Institute for Geography and Statistics, IBGE). [↑](#endnote-ref-1)
2. Details on how this was defined are not available in the paper. [↑](#endnote-ref-2)
3. The paper reported: ≤R$ 510 and >R$ 510 (time period of income is not reported); currency rate used: 1 Brazilian Real=0.5814 US$ (given in the article). [↑](#endnote-ref-3)
4. Corrected (empirical Bayesian method) leprosy detection coefficient (number of new cases divided by the population of its area multiplied by 10,000 inhabitants) in 2006. [↑](#endnote-ref-4)
5. Non-corrected leprosy detection coefficient in 2006. [↑](#endnote-ref-5)
6. From abstract but does not correspond with Table 2 in the paper. [↑](#endnote-ref-6)
7. Wealth quintiles based on house structure (floor, wall and roof), electricity, ownership of radio, television, computer, mobile phone, refrigerator, fan, air conditioner, wardrobe, table, chair, watch/clock, bicycle, motorcycle/scooter, tractor/motorized farm equipment, local rice husking equipment, owns livestock, owns house, owns land house, owns farmland, toilet facilities. [↑](#endnote-ref-7)
