# Supplementary material for: Socioeconomic Inequalities in Neglected Tropical Diseases: A Systematic Review
Source: PLoS Negl Trop Dis. 2016 May 12;10(5):e0004546. doi: 10.1371/journal.pntd.0004546 (PMC4865383; doi:10.1371/journal.pntd.0004546)
Supplement: S7 Table — (DOCX) [file pntd.0004546.s009.docx]

**S7 Table: Summary of the literature on socioeconomic inequalities in visceral leishmaniasis, 2004-2013.**

| **Top 20 GBD 2010;**  **Author, Year** | **Aim of study** | **Outcome,**  **detection method** | **Study design, statistical method, sample size** | **Study sample (period, area, population, age, randomization)** | **Measure of SEP** | **Strata** | **Prevalence**  %  (N inf/total N) | **Univariate association**  OR (95% CI) | **Multivariate association**  OR (95% CI)  **(Adjusted for…)** |
| --- | --- | --- | --- | --- | --- | --- | --- | --- | --- |
| #1, India;  Boelaert M *et al.*, 2009 | To study socio-economic household profiles in the context of a community intervention trial on the effect of long-lasting impregnated bed nets in the prevention of VL | VL incidence;  VL cases were obtained from the public health system, private and charitable hospitals | Ecological design;  Student’s t-test;  N=2,013 households in high endemic area, 2,997 households in Bihar state | 2003-2006;  Muzaffarpur district, northern Bihar state, India;  Households;  All ages;  35 high endemic clusters were identified based on the number of VL cases followed by a house to house survey to evaluate the VL incidence rate during the preceding 3 yrs; 16 of these clusters were included^[[1]](#endnote-1)^ | Type of house  Electricity  Bicycle  Motorcycle/ scooter  Car/tractor  Radio  TV  Education household head | Kachcha (mud)  Semi-pucca  Pucca (stone/bricks)  Yes  Yes  Yes  Yes  Yes  Yes  Illiterate | (N high endemic clusters/ N Bihar state)  (1,418/794)  (477/1,248)  (118/955)  (193/1,212)  (839/1,646)  (46/338)  (10/96)  (197/983)  (136/864)  (1,354/1,588)  Overall prevalence: NR | % in high endemic cluster *vs.* % in general population of Bihar state  71% vs. 27%, p<0.001  24% vs. 42%, p<0.001  6% vs. 32%, p<0.001  9.6% vs. 40%, p<0.001  42% vs. 55%, p<0.001  2.3% vs. 11%, p<0.001  0.5% vs. 3.2%, p<0.001  9.8% vs. 33%, p<0.001  6.8% vs. 29%, p<0.001  67% vs. 53%, p<0.001 | NR |
| #1, India;  Bhunia GS *et al.*, 2012 | To develop a geo-environ-mental risk map for VL using environmental and demographic parameters | VL incidence;  Incidence data from the District Health Offices and  Bihar State Health Society | Ecological design;  Pearson’s correlation coefficient;  N=  2,146,065 | 2007 – VL incidence, 1990-2001 – SEP data;  Vaishali district, Bihar state, India;  Villages;  All ages;  Population-based | Illiteracy rate  Percentage unemployed |  | Overall prevalence: NR | Correlation at village level  r=0.43, p<0.03  r=0.32, p<0.009  Villages with illiteracy rates > 60% and percentage unemployed > 70% were marked as having a high risk of disease transmission | NR |
| #1, India;  Hasker E *et al.*, 2012 | To study factors associated with VL adjusted for confounding by socioeconomic status | VL incidence;  clinical VL history (fever of >2 weeks’  duration, lack of response to antimalarial drug treatment), positive rK39 rapid diagnostic test and  good response to specific VL treatment | Cohort study;  Binomial multilevel model with hamlets as random effect;  N=81,210 from 13,416 households | 2008-2010;  Muzaffarpur district, Bihar State, India;  Household members;  All ages;  All households in the study area were included | Wealth quintile^[[2]](#endnote-2)^  Mushahar caste | 1 (poorest)  2  3  4  5 (least poor) | (70/16,515)  (58/16,094)  (31/16,124)  (35/16,256)  (13/16,221)  32/1,980  Average annual incidence: 72.8 per 100,000 | NR | 1 (ref)  0.9 (0.6-1.3)  0.7 (0.5-1.1)  0.9 (0.6-1.4)  0.5 (0.3-1.0)  2.9 (1.3–6.8)  (Mushahar caste (who are the poorest of the poor), wealth quintile, age group, ownership of goats, bamboo tree at < 10 m, type of walls) |
| #1, India;  Ranjan A *et al.*, 2005 | To determine whether socioeconomic, behavioral, and household characteristics and lack of proper vector control are risk factors for the occurrence of VL | VL infection;  Cases: clinically and parasitologically confirmed, Controls: no clinical symptoms | Case-control design;  Logistic regression;  N=134 cases, 406 controls (matched by age and gender) | 2001-2003;  Patna, Vaishali, Muzaffarpur, Samastipur, and Nalanda districts (typical disease-endemic regions), Bihar state, India;  Citizens;  All ages;  Cases: VL patients admitted to Rajendra Memorial Research Institute  of Medical Sciences in Patna,  Controls: Households without VL cases in the family^[[3]](#endnote-3)^ | Household asset index^[[4]](#endnote-4)^  Education | Low  Medium  High  Illiterate  Literate | (cases/controls)  (114/350)  (18/52)  (2/4)  (65/147)  (69/259) | % in cases vs. controls  85% vs. 86%  13.5% vs. 13%  1.5% vs. 1%  p=0.86  1.66 (1.10-2.51), p=0.01  1 (ref) |  |
| #1, India;  Saha S *et al.*, 2012 | To estimate VL incidence and identify risk factors for infection one year after one round of DDT spraying and a round of mass screening and treating infected people | VL incidence;  Direct agglutination  test | Retrospective cohort design;  Logistic regression;  N=751 | 2004-2005;  Chatrakhali village, West Bengal, India  Villagers;  All ages;  Entire village population, excluding people that did not participate in the  2004 survey which was conducted at time of mass screening, and excluding persons who were unwilling to participate or who migrated out/in after 2004 | Illiteracy  Below poverty line | Yes  No  Yes  No | 20% (71/353)  20% (79/398)  22% (123/557)  14% (27/194)  Overall prevalence: 20% | Relative Risk (95%CI)  1.0(0.8-1.3)  1 (ref)  1.6 (2.2-14)^[[5]](#endnote-5)^  1 (ref) |  |
| #1, India;  Singh SP *et al.*, 2010 | To investigate the association between VL and keeping domestic animals inside the house at night | VL infection;  Parasitologically confirmed or positive rK39 result and successfully treated | Case-control design;  Conditional logistic regression;  N= 141 cases, 282 controls  (matched on neighborhood, age and sex) | 2007-2008;  Muzaffarpur district, Bihar state, India;  Villagers, mainly farmers and daily wages earners;  >=2 yrs;  Cases: identified during an initial household survey covering the entire study area as well as from records of government primary health care and private medical facilities,  Controls: selected starting from the 2 houses nearest to the house of the case | Household asset index^[[6]](#endnote-6)^ | 4 (poorest)  3  2  1 (least poor)  Continuous | (cases/controls)  (46/77)  (42/75)  (27/53)  (26/77)  Overall incidence in the study region: 107 per 100,000 | 2.00 (1.05-3.83)  1.75 (0.95-3.19)  1.48 (0.80-2.77)  1 (ref)  1.26 (1.02-1.54) |  |
| #3, Bangladesh;  Bern C *et al.*, 2005 | To study spatial patterns and risk factors for kala-azar in a highly affected community in the sub-district with highest VL incidence in Bangladesh | Cumulative VL incidence;  rK39 ELISA method | Cross-sectional design;  Logistic regression adjusted for within-household correlation;  N=2,439 people from 506 households | 2002-2004;  Fulbaria sub-district,  Mymensingh district, Bangladesh;  Household members;  All ages;  Population based: all residents living in the study area for >6 months in the 3  years before the 2002 survey were included | Household head can read  Monthly income/ person  Owning bicycle  Owning radio | No  Yes  <$10  ≥$10  No  Yes  No  Yes | 6.4% (100/1,560)  7.4% (48/652)  7.3% (81/1,112)  6.1% (67/1,096)  6.8% (138/2,026)  5.4% (10/186)  7.2% (124/1,715)  4.8% (24/497)  Overall prevalence: 9% (VL or a history of VL) | 1 (ref)  1.2 (0.78-1.83), p=0.41  1 (ref)  0.82 (0.55-1.22), p=0.33  1 (ref)  0.71 (0.36-1.42), p=0.33;  1 (ref)  0.65 (0.39-1.08), p=0.10 | NR |
| #3, Bangladesh,  Ferdousi F *et al.*, 2012 | To provide information on the VL burden in endemic communities with active community-based VL surveillance to identify risk factors associated with VL in these areas | Clinical VL;  Positive rapid rK39 dipstick test + confirmed by govt. hospital | Prospective cohort design;  Poisson regression analysis;  N= 6,761 people from 1,550 households | 2006-2008;  8 villages in Trishal sub-district, Mymensingh district, Bangladesh;  Citizens living in mud-wall houses;  ≥ 3 yrs;  2 high endemic unions of Trishal sub-district were chosen and 8 villages were selected by multi-cluster random sampling. All inhabitants living in mud-wall houses were included in the study | Having electricity in the house (N=1,550)  Education household head (yrs, N=1,550) | No  Yes  0  1-5  >5 | (239/1,382)  (9/168)  (total N)  (1,129)  (232)  (189)  Overall incidence:  2006: 141.9/ 10,000  2007: 196.7/10,000  2008: 28.1/10,000 | 3.40 (1.76-6.59), p<0.001  1 (ref)  p>0.05 | 2.99 (1.56-5.75), p<0.01  1 (ref)  (Age, gender, use of mosquito-control measures at night and use of bed-net at night) |
| #5, Ethiopia,  Argaw D *et al.*, 2013 | To identify factors associated with VL risk in residents and  migrants | VL infection;  past VL case: at least 2 weeks fever + weight loss and/or splenomegaly, diagnosed based on DAT+ or rK39 dipstick +  treated with clinical resolution of  symptoms or a patient with *Leishmania* amastigotes;  Current VL case: at least 2 weeks fever+ weight loss  and/or splenomegaly, *Leishmania* amastigotes and/or DAT+ or rK39 dipstick + | Case-control design;  Conditional logistic regression;  N=151 cases, 302 controls (matched by age and gender) | 2009;  Kafta-Humera district, Tigray regional state, Ethiopia  Citizens of Kafta-Humera district;  All ages  Cases: identified both retrospectively  from hospital records and prospectively for patients  diagnosed during the recruitment period  Controls: selected from the two neighboring  houses nearest to the case | Owns land  Owns no land or <4 hectare land  Monthly expenditure <8.89 US$ /person^[[7]](#endnote-7)^  Household head can write his name  Household head can read  Household head had *no* formal schooling  Household head left school < class 5  Household head works as laborer | No  Yes  Yes  No  Yes  No  No  Yes  No  Yes  Yes  No  Yes  No  Yes  No | (cases/controls)  (65/125)  (86/177)  (129/264)  (22/38)  (40/36)  (111/266)  (59/93)  (89/208)  (62/95)  (88/207)  (61/97)  (89/205)  (124/209)  (26/93)  (41/45)  (109/257) | 1 (ref)  0.92 (0.59-1.43), p=0.71  0.84 (0.48-1.49), p=0.56  1 (ref)  2.81 (1.66-4.76), p<0.001  1 (ref)  1 (ref)  0.69 (0.46-1.03), p=0.07  1 (ref)  0.66 (0.44-0.98), p=0.04  1.41 (0.95-2.09), p=0.09  1 (ref)  2.91 (1.61-5.28), p<0.001  1 (ref)  2.47 (1.44-4.25), p=0.001  1 (ref) | 3.22 (1.42-7.33), p=0.01  1 (ref)  2.78 (1.17-6.59), p=0.02  1 (ref)  (Always slept under net in rainy season, Slept under acacia at night, usually slept on ground, walls of thatched grass on wood frame) |
|  |  |  | N=157 cases, 314 controls  (mainly males, matched by age and gender) | 2009-2011;  Migrants;  All ages;  Cases: Retrospectively collected from hospital, with recruitment concentrated in the months with  higher migrant influx  Controls: recruited among  migrants presenting to the hospital with any condition other than VL or malaria;, recruited within a week of the corresponding  case | Household head had no  formal schooling  Household head left school < class 5 | Yes  No  Yes  No | (113/177)  (46/140)  (83/84)  (76/233) | 3.50 (2.2-5.4), p<0.001  1 (ref)  2.12 (1.36-3.31), p=0.001  1 (ref) | 5.02 (2.59-9.74), p<0.001  1 (ref)  (HIV infection, ever slept under net, slept under acacia at night, slept near dogs, staple food is porridge) |
| #11 and #14, Uganda and Kenia;  Kolaczinski JH *et al.*, 2008 | To identify individual and household level risk factors for VL in eastern Uganda/north-western Kenya | VL infection;  Suspected clinical VL confirmed by rK39 antigen-based  dipstick | Case-control design;  Conditional logistic regression;  N=93 cases, 226 controls | 2006;  West Pokot district, north-western Kenya, and Pokot county in Nakapiripirit district, eastern Uganda;  Citizens of Pokot area (mainly Pokot pastoralists);  All ages;  Cases: (Kenyan & Ugandan) patients from out-patients department of Amudat hospital in Uganda.;  Controls: randomly selected from same communities as cases and matched by sex, age and village | Wealth quintiles^[[8]](#endnote-8)^ | 1 (poorest)  2  3  4  5 (least poor) | (cases/controls) (32/31)  (43/20)  (45/17)  (46/14)  (59/7) | 1 (ref)  0.40 (0.18-0.90)  0.26 (0.11-0.62)  0.19 (0.08-0.46)  0.08 (0.03-0.23) | 1 (ref)  0.36 (0.14-0.94)  0.19 (0.06-0.58)  0.12 (0.04-0.39)  0.17 (0.05-0.65)  (Sleeps near animals, household applies insecticide to livestock, knows VL symptoms, has mosquito net) |
| #19, Brazil;  de Almeida AS *et al.*, 2011 | To identify areas at greatest risk of VL in urban areas | Smoothed VL incidence;  Brazilian Reportable Diseases  Information System (SINAN) | Ecological design;  Spatial analysis;  N=756 cases, 779,939 inhabitants | 2001-2006;  Teresina, the capital of Piaui state, Brazil;  Citizens of Teresina;  All ages;  Population-based | Illiteracy rate  Mean income of household head |  | Overall prevalence: NR | Bivariate global Moran’s I index ( if >0: positive correlation between indicator and VL incidence)  0.34, p<0.01  -0.22, p<0.01; | NR |
| #19, Brazil;  De Araujo VEM *et al.*, 2013 | To identify the risk areas for human VL and the risk factors involved in transmission | VL incidence;  Brazilian Reportable Diseases  Information System (SINAN) | Ecological design;  Spatial analysis;  N=412 cases, 2,375,151 inhabitants | 2007-2009;  Belo Horizonte, capital of Minas Gerais state, Brazil;  Citizens of Belo Horizonte;  All ages;  Population-based | Education  Income | Illiteracy rate (%)  % of household heads with < 4 yrs of education  % of household heads with <2 minimum wages  Average income^f^ (inverted) of household head | Overall incidence: 2007: 4.9 per 100,000  2008: 7.2 per 100,000  2009: 6.6 per 100,000 | Coefficient (95% CI)  3.2 (0.2-6.1)  1.8 (0.8-2.8)  1.2 (0.4-1.9)  2.0 (1.1-3.0) | Best model from 4 models  1.7 (0.8-2.7)  (Number of infected dogs per inhabitants) |
| #19, Brazil;  Borges BKA *et al.*, 2008 | To evaluate knowledge and attitudes concerning VL prevention | VL infection;  Brazilian Reportable Diseases  Information System (SINAN) | Case-control design;  Logistic regression;  N=82 cases, 164 controls | 2006  Belo Horizonte, capital of Minas Gerais state, Brazil;  Citizens of Belo Horizonte;  All ages;  Population-based | Education^[[9]](#endnote-9)^ | No education  Primary  1^st^ grade not completed  1^st^ grade completed  2^nd^ grade not completed  2^nd^ grade completed  3^rd^ grade not completed  3^rd^ grade completed | (cases/ controls)  (22/7)  (34/34)  (4/36)  (3/26)  (8/23)  (7/28)  (2/5)  (2/5) | 8.22 (NR), p<0.001  0.31 (NR), p=0.02  0.03 (NR), p<0.001  0.03 (NR), p<0.001  0.11 (NR), p<0.001  0.07 (NR), p<0.001  0.12 (NR), p=0.03  0.12 (NR), p=0.03 | 0.64 (0.53-0.77), p<0.001^[[10]](#endnote-10)^  (Age, gender, knowledge about VL) |
| #19 Brazil,  Gouvea MV *et al.*, 2007 | To identify socioeconomic and environmental  factors associated with Montenegro skin test  positivity in an urban area with a high force of transmission | Prevalence of *L.Chagasi* infection;  Montenegro skin test (MST) | Cross-sectional design;  Poisson  regression model with robust variance;  N=1,106 | 2004;  Teresina, the capital of Piaui state, Brazil;  All citizens of the included neighborhoods;  All ages;  10 areas in 7 out of 105 neighborhoods were selected; neighborhoods were selected in order to cover a variety of urban contexts and a range of transmission patterns.^[[11]](#endnote-11)^ | Household head education  (N=1,091) | Only elementary  >elementary | (total N)  48.3% (236)  33.6% (855)  Overall prevalence: 36.7% | Prevalence ratio (PR)  1 (ref)  0.69 (0.59-0.82), p<0.001 | PR  1 (ref)  0.80 (0.67-0.96), p=0.01  (Gender, age, number of people in household, time of dog ownership) |
| #19, Brazil,  Karagiannis-Voules D-A *et al.*, 2013 | To estimate the number of CL and VL infected people at the state level to generate incidence maps | VL incidence;  Information system for notifiable diseases (SINAN) | Ecological design;  Bayesian  geostatis-tical negative binomial model with spatially structured  random effects at municipality level;  N=2,176 municipalities | 2001-2010;  2,176 municipalities of Brazil;  Citizens of the municipalities;  All ages;  Municipalities  with reported cases  for at least one year between 2001 and 2010 were selected | % people that own their house  % people with sanitation at home | <81.5%  81.5-87.2%  87.2-90.8%  >90.8%  <2%  2-25%  >25% | Total prevalence: NR | NR | Bayesian geostatistical model  Incidence Rate Ratios  1 (ref)  0.88 (0.77-1.00)  0.88 (0.77-1.01)  0.71 (0.61-0.83)  1 (ref)  0.91 (0.82-1.02)  0.62 (0.54-0.73)  (Altitude, mean diurnal range, annual precipitation (mm), precipitation in warmest and coldest quarter (mm), period (2001-2005/2006-2010) |
| #19, Brazil,  Lima ID *et al.*, 2012 | To determine the extent of VL infection among people residing in an endemic area | VL infection: based on joint result of two diagnostic tests for VL (skin test,LST and blood test, SLA);  Leishmania skin test (LST) and anti-leishmanial antibodies in blood samples  using rK39 antigen and soluble lysate of *L.i.*  *chagasi* (SLA), an isolate from a patient with VL in Natal  that was typed in a World Health Organization Reference  Laboratory | Cross-sectional design;  Multinomial logistic analysis;  N=345 | 1990-2010;  Parnamirim, Natal, state of Rio Grande do Norte, Brazil;  Household members of urban, peri-urban, and rural areas;  All ages;  268 households were selected through  a random point pattern generated without prior knowledge of the houses in the vicinity. The closest  household to each point was selected using a GPS | Income  (monthly minimal wages, approx. 290 US$/month^[[12]](#endnote-12)^, N=319) | Diagnosis groups  LST-/SLA-  LST-/SLA+  LST+/SLA-  LST+/SLA+ | (N diagn.group / total N)  Overall prevalence: 39.4%  (LST+ 38.6%, SLA+ 24.6%) | Mean monthly minimal wages ± SD  2.0 ± 1.1  2.2 ± 1.1  2.1 ± 1.1  2.0 ± 1.0  p=0.47 | NR |
| #19, Brazil,  de Oliveira ALL *et al.*, 2008 | To verify the prevalence of asymptomatic VL infection in household contacts | VL infection;  Clinical manifestations of VL  associated with observation of amasti-gote forms in Giemsa-stained bone marrow smears or  serology using indirect immune-fluorescence (IIF) | Cross-sectional design;  Chi-square test with Yates correction;  N=220 contacts from 46 families | 2002;  Três Lagoas, Mato Grosso do Sul state, Brazil;  Asymptomatic contacts  of visceral leishmaniasis cases;  All ages;  46 out of 60 families with patients with clinical manifestations of VL confirmed during the study period  by the Três Lagoas Municipal Health Department | Monthly income (number of minimum wages)  Education of household head  Household assets | 1-2  3-5  >5  Unknown  Incomplete primary  Completed primary and incomplete secondary  Secondary and university  Not reported  Stove, refrigerator or TV  Stove, TV, refrigerator  Stove, TV, refrigerator, stereo | Distribution of positive contacts (%) (N all contacts, N families)  30.2% (111, 23)  37.7% (45, 11)  28.0% (50, 10)  4.1% (14, 4)  35.0% (92, 21)  37.5% (55, 9)  15.0% (27, 5)  0% (46, 11)  17.5% (36, 7)  76.3% (162, 36)  6.2% (22, 3)  Total prevalence: 36.4% | p=0.43  p=0.08  p<0.001 | NR |

NR: Not Reported; VL: Visceral Leishmaniasis; CL: Cutaneous Leishmaniasis; DAT: antigen from the Royal Tropical Institute, Amsterdam, The Netherlands; ELISA: two enzyme-linked immunosorbent assays; inf: infected.

1. 16 clusters were selected based on 5 characteristics: (1) reporting VL cases in each year of the 3-year-period; (2) average of at least 0.8% VL incidence rate over the past 3 years; (3) highest incidence rates; (4) population >350 and <1500; (5) minimum distance of 1 km between clusters. [↑](#endnote-ref-1)
2. Based on information about assets owned other than domestic animals. [↑](#endnote-ref-2)
3. Households without VL cases in the family were identified and a list of healthy controls matched for age and gender was made: 3 controls for each case were randomly selected from the list. [↑](#endnote-ref-3)
4. Based on house type, availability of electricity, fuel used for cooking, presence of a lavatory facility, and possession of household consumer items including car, scooter/motorcycle, television, radio, sewing machine, electric fan, and bicycle. [↑](#endnote-ref-4)
5. Confidence interval as reported in paper appears to be incorrect (it does not embrace the point estimate). [↑](#endnote-ref-5)
6. Asset index based on: ownership of land, motorcycle(s), bicycle(s), television set(s), radio(s), mobile phone(s), watch(es), fan(s), mattress(es) and bed(s). [↑](#endnote-ref-6)
7. The paper reported: monthly expenditure <100 Ethiopian birr/person; currency rate used: 1 Ethiopian birr=0.0889 US$, June 1, 2009 (mid of study period, www.xe.com). [↑](#endnote-ref-7)
8. Based on information about ownership of household assets. [↑](#endnote-ref-8)
9. In Brazil 1st grade stands for primary education, 2nd grade for secondary education (high school) and 3rd grade for university. [↑](#endnote-ref-9)
10. It remains unclear how education was entered in the final model. [↑](#endnote-ref-10)
11. Each area was divided into blocks encompassing 25 residences on average, using topographic maps. Four blocks per area were selected using simple random sampling, leading to a sample of 40 blocks. In each block, all residences were visited and one person with no VL history among all household residents was selected using simple random sampling. [↑](#endnote-ref-11)
12. The paper reported: monthly minimal wages, approx. R$ 540/month; currency rate used: 1 Brazilian Real=0.5376 US$, January 1, 2000 (mid of study period, [www.xe.com](http://www.xe.com)). [↑](#endnote-ref-12)
